# Highly Conductive Topologically Chiral Molecular Knots as Efficient Spin Filters

**DOI:** 10.1021/jacs.3c08966

**Published:** 2023-11-16

**Authors:** Dan-Yang Zhang, Yutao Sang, Tapan Kumar Das, Zhao Guan, Ni Zhong, Chun-Gang Duan, Wei Wang, Jonas Fransson, Ron Naaman, Hai-Bo Yang

**Affiliations:** † Shanghai Key Laboratory of Green Chemistry and Chemical Processes & Shanghai Frontiers Science Center of Molecule Intelligent Syntheses & Chang-Kung Chuang Institute, School of Chemistry and Molecular Engineering, East China Normal University, Shanghai 200062, China; ‡ Department of Chemical and Biological Physics, Weizmann Institute of Science, Rehovot 7610001, Israel; § Key Laboratory of Polar Materials and Devices (MOE) and State Key Laboratory of Precision Spectroscopy, East China Normal University, 500 Dongchuan Rd., Shanghai 200241, China; ∥ Collaborative Innovation Center of Extreme Optics, Shanxi University, Taiyuan 237016 Shanxi, China; ⊥ Institute of Eco-Chongming, Shanghai 202162, China; # Department of Physics and Astronomy, Uppsala University, Uppsala 75236, Sweden; ¶ State Key Laboratory of Molecular Engineering of Polymers, Department of Macromolecular Science, Fudan University, Shanghai 200438, China

## Abstract

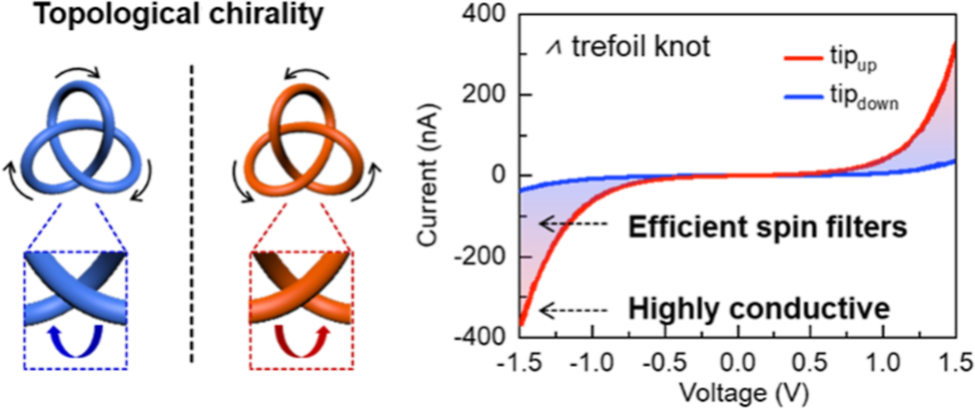

Knot-like structures were found to have interesting magnetic properties in condensed matter physics. Herein, we report on topologically chiral molecular knots as efficient spintronic chiral material. The discovery of the chiral-induced spin selectivity (CISS) effect opens the possibility of manipulating the spin orientation with soft materials at room temperature and eliminating the need for a ferromagnetic electrode. In the chiral molecular trefoil knot, there are no stereogenic carbon atoms, and chirality results from the spatial arrangements of crossings in the trefoil knot structures. The molecules show a very high spin polarization of nearly 90%, a conductivity that is higher by about 2 orders of magnitude compared with that of other chiral small molecules, and enhanced thermal stability. A plausible explanation for these special properties is provided, combined with model calculations, that supports the role of electron–electron interaction in these systems.

## Introduction

In the last decades, there has been an increasing interest in the properties of topological materials, namely, materials with properties that are invariant under topological transformations. Specifically, in physics, there has been an increasing interest in the electro-magnetic characteristics of materials, in which space inversion symmetry is broken, and particularly in knot-like structures.^1,2^ These materials were shown to have unconventional ferromagnetism or unconventional antiferromagnetism. Chemists succeeded to synthesize diverse knotted structures;^3,4^ however, their electronic or magnetic properties have never been investigated yet.

**Figure 1:**
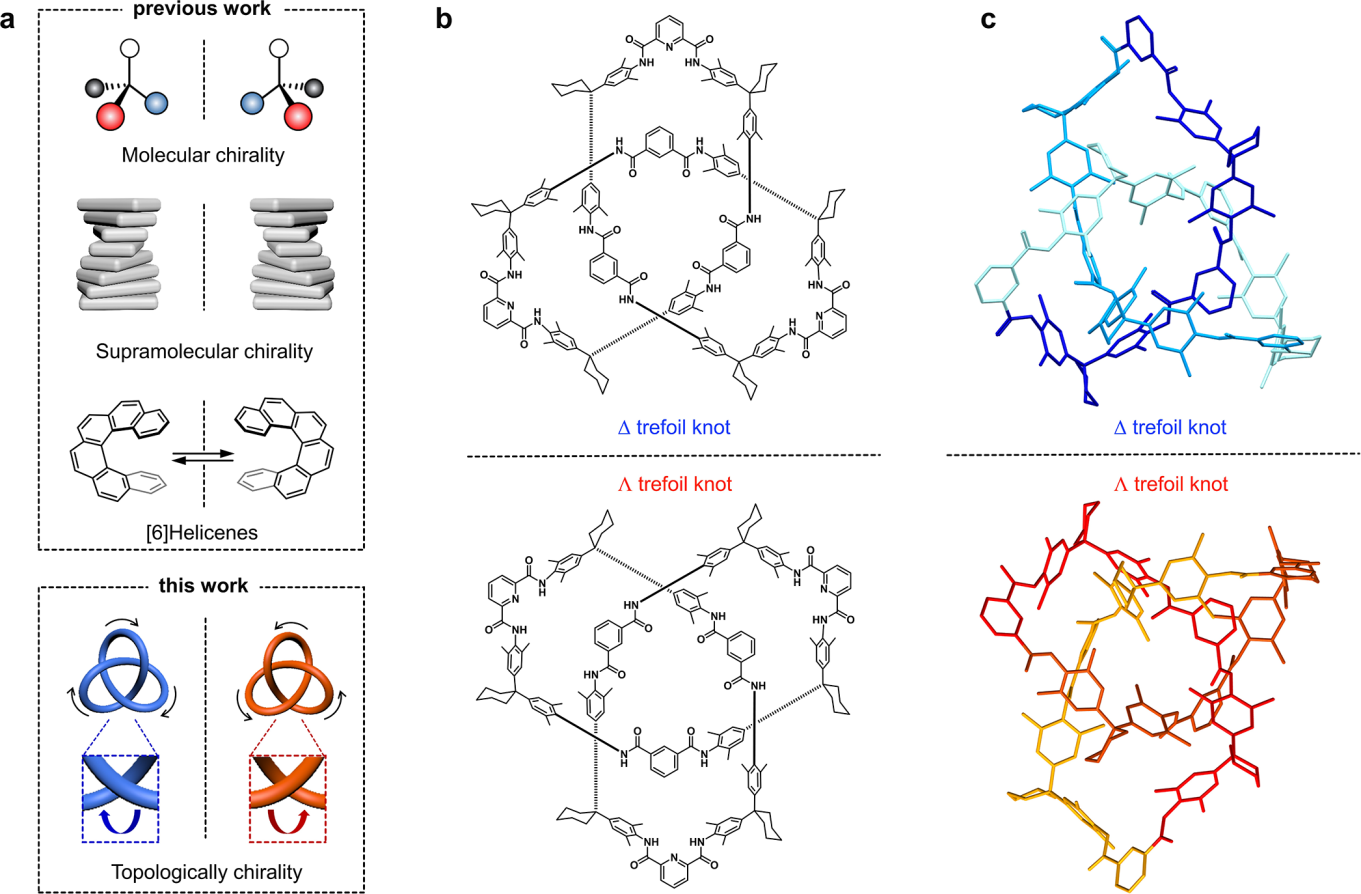
Schematic description of the chiral materials used in spin control and chiral knots. (a) Comparison of different types of chirality used in the CISS-related studies. Molecular chirality: a carbon center with four different attached substituents. Supramolecular chirality: chiral packing of chiral or achiral monomers. (b,c) Chemical (b) and single crystal (c) structures of topologically chiral molecular knots used in this work. There are no classical stereogenic units within the closed loop; however, the entanglements endow the resulting trefoil knot molecules with topological chirality depending on the spatial arrangements of crossings.

The chiral-induced spin selectivity (CISS) effect has been extensively studied during the past 2 decades.^5,6^ It was shown that the transport of electrons through chiral objects, such as chiral molecules, supramolecules, crystals, and films, depends on the electrons’ spin. Therefore, each spin state correlates with efficient transport through a system of specific handedness. Recent reports have demonstrated a correlation between the chiral optical activity and spin polarization.^7−9^ Understanding this structure–spin selectivity relation requires exploring many different structures with various chiral-induced elements. Indeed, during the last 2 decades, several studies have been performed on molecules containing asymmetric carbon, molecules that have in addition, a chiral secondary structure such as DNA and oligopeptides,^10^ polymers, and helicenes,^11−15^ which lack asymmetric carbon but have a chiral secondary structure, and various chiral oxide films in which the chirality results from the chiral induction by small chiral molecules while the bulk crystal structure of oxides is usually achiral (see Figure 1a).^16−19^ The CISS effect should also appear in the knot-like molecules since they are chiral, despite the fact that they do not contain classical stereogenic units. The entanglements endow the resulting trefoil knot molecules with topological chirality, depending on the spatial arrangements of crossings (Figure 1b).

Herein, we present an investigation of the spin-selective transport in a molecular trefoil knot. Indeed, helicenes are ortho-condensed polycyclic aromatic compounds without any stereogenic carbons; their benzene rings or other aromatics are angularly annulated, resulting in a helically shaped skeleton. However, helicenes are generally stable under mild conditions but can be transformed into the opposite handedness once the temperature reaches the enantiomerization barrier. Note that helicenes with more than six benzene rings have a much higher barrier. In contrast to helicene, the only way to transfer a topologically chiral molecule into its mirror form would be to break at least one covalent bond and reconnect it in the opposite direction. Moreover, compared to molecular and supramolecular chirality, topological chirality refers to a higher-level spatial organization, thus avoiding the orientation issue. This property is especially important when considering molecules assembled on surfaces.

## Results and Discussion

**Figure 2:**
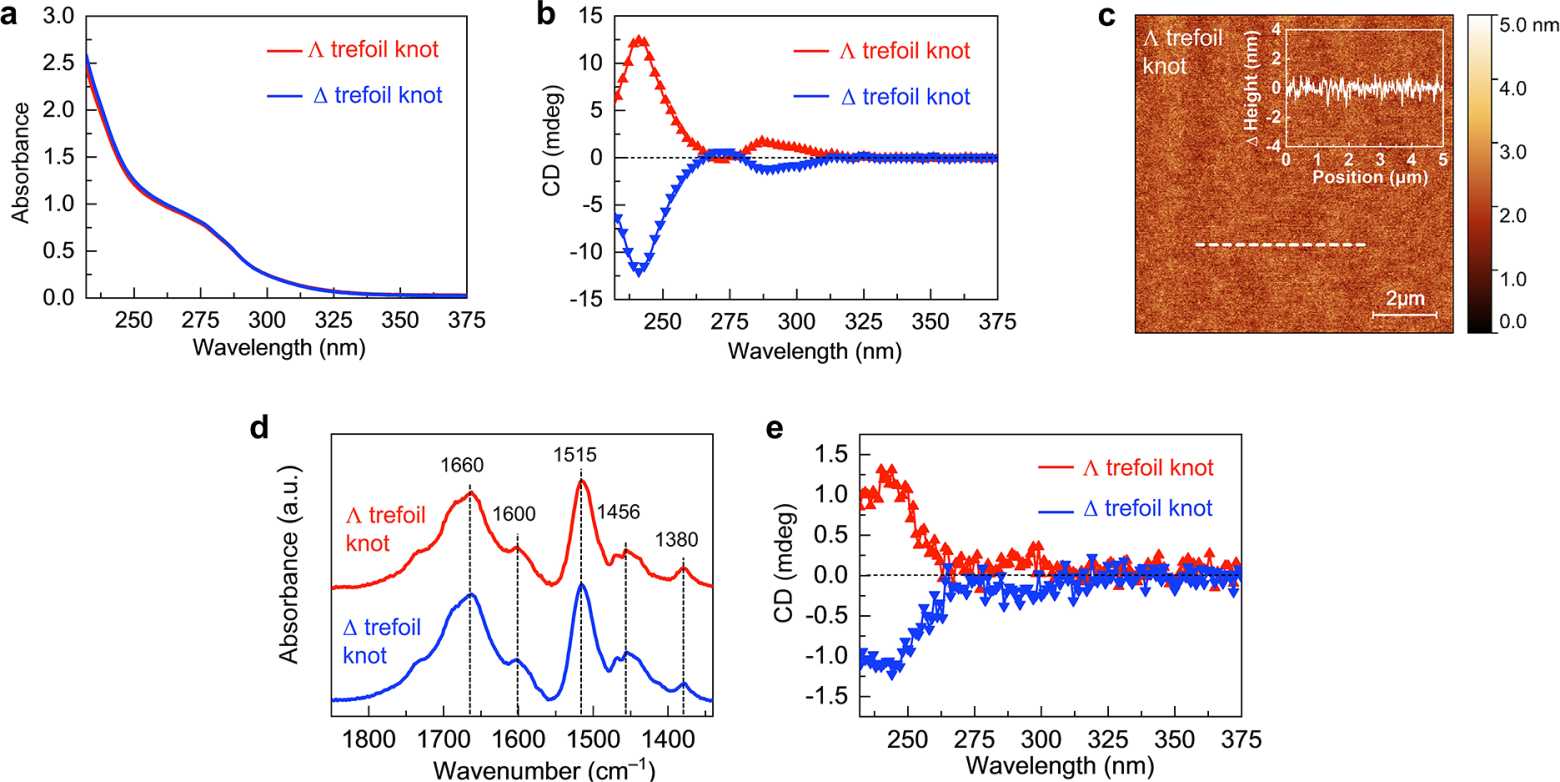
Spectroscopic and morphological characterizations. (a,b) Absorbance (a) and electronic CD spectra (b) of solution-state ∧ and Δ molecular trefoil knots (10 μM in CH_2_Cl_2_). (c) AFM topography images of the ∧ molecular trefoil molecular knot thin films. Insets: AFM height profile of the white line drawn in the AFM image. (d,e) PM-IRRAS (d) and CD spectra (e) of the trefoil knot thin films. For AFM characterization, thin films were prepared on HOPG, which is consistent with the following mCP-AFM measurements: thin films were prepared on a Au surface and quartz for PM-IRRAS and CD measurements, respectively.

The original synthesis of the trefoil knots was reported by Vögtle et al.^3^ Typically, the molecular knots were synthesized as a racemic mixture and then separated by high-pressure liquid chromatography (HPLC) on a Chiralpak-IF-type column (Figures S1–S4). Syntheses and characterization of these compositions are detailed in the Supporting Information. The topological chirality of molecular trefoil knots can be directly observed from circular dichroism (CD) spectra (Figure 2b) and single-crystal X-ray structures obtained from racemic mixture (Figures S5 and S6 and Table S1). For instance, ∧ and Δ trefoil knots show mirror symmetrical CD signals with three maxima at 240, 272, and 293 nm, which is consistent with the literature reports.^3^ With enantiopure molecular trefoil knots in hand, we used spin coating to prepare thin films of a thickness of about 3.0 nm on different substrates due to the need to use different substrates for different characterization methods. The films had low roughness. For example, for highly oriented pyrolytic graphite (HOPG) substrates, the root mean square average of the thin films is ±1 nm, suggesting a uniform morphology (Figures 2c and S7).

The thin films were further characterized using polarization modulation–infrared reflectance–absorption spectrometry (PM-IRRAS, Figure 2d). The strong vibrational band at around 1660 cm^–1^ is attributed to the N–H bending (amide-I); the peaks at around 1600, 1515, and 1456 cm^–1^ are characteristic of the skeleton vibration of the benzene ring and pyridine. The symmetry bending vibration of the methyl groups (−CH_3_) at 1380 cm^–1^ was also observed. We also investigated the solid-state (thin films) properties of the molecular trefoil knots. Here, the enantiopure molecular knots were spin coated on clean quartz substrates and then measured by CD. Figure 2e shows that the thin film is also CD active, displaying the mirror image spectra of the two enantiomers. The CD spectra were identical when measured from different angles, thus eliminating the contamination of linear dichroism (Figure S8). Note that the sign of the CD signals and the characteristic peaks in the thin films are consistent with the spectra obtained in solution (Figure 2b), indicating that the topological chirality is well maintained in the solid-state thin films.

**Figure 3:**
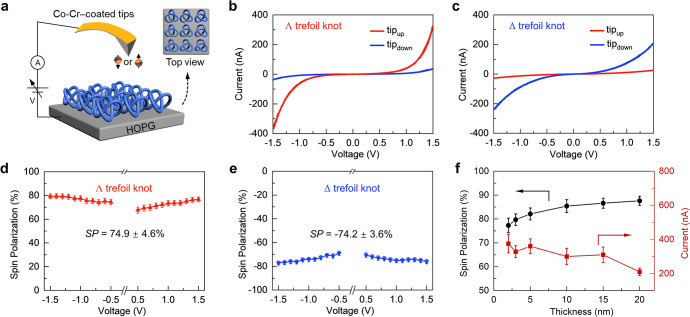
mCP-AFM measurements. (a) Schematic illustration of an mCP-AFM setup. (b,c) Current–voltage curves of the ∧ (b) and Δ (c) molecular trefoil knot thin films measured by mCP-AFM at room temperature. The current–voltage curves were measured 50 times at different spots on the substrate. The lines represent the average results. The raw data and the absolute direction of current and magnetization are presented in the Supporting Information (Figure S9). Tip_up_, AFM tip magnetized up and Tip_down_, AFM tip magnetized down. (d,e) Spin polarization as a function of the applied bias for ∧ (d) and Δ (e) molecular trefoil knots. Spin polarization was calculated based on the results shown in (b,c). (f) Thickness-dependent absolute spin polarization and absolute current intensities at ±1.5 V.

There are numerous methods for monitoring the spin-polarized charge transport through molecules. Here, we relied mainly on magnetic conductive probe atomic force microscopy (mCP-AFM), a well-established method for evaluating the ability of spin filtering in chiral materials (Figure 3a).^15^ Ferromagnetic Co–Cr–coated tips were first magnetized by an external magnet (∼0.5 T) with the opposite magnetization directions, tip_up_ or tip_down_, when the “north pole” of the magnet pointed either away or toward the substrate (HOPG), respectively. Immediately after the magnetization, the tips were used in the mCP-AFM measurement. During the measurement process, an electric bias potential was applied with respect to the substrate (see more details in Figure S9). Figure 3b,c shows the typical current–voltage (*I*–*V*) characteristics of the molecular trefoil knot thin film in the mCP-AFM measurement at room temperature. The ∧ trefoil knot showed a much higher current when the tip was magnetized up than when it was magnetized down. In contrast, an opposite trend was observed for the Δ trefoil knot; the measured current was higher when the tip was magnetized down than when it was magnetized up. We also checked the racemic mixture samples containing 50% ∧ and 50% Δ trefoil knots as a controlled experiment. We found no difference between tip_up_ and tip_down_ (Figure S10) in this case.

The spin polarization, as a function of the electric potential applied, SP(V), is defined as
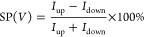
where *I*_up_ and *I*_down_ are the currents measured at a given potential, *V*, when the tip magnetic field is pointing up or down, respectively. Based on the results shown in Figures 3b,c, the calculated spin polarizations are 74.9 ± 4.6% and −74.2 ± 3.6% for the ∧ and the Δ molecular trefoil knot thin films, respectively (Figure 3d,e). The spin polarization is almost constant for a potential above 0.5 V. Such spin polarization is significantly higher than that reported for most of the chiral self-assembled monolayer (SAM) systems (typically in the range of 30 to 50%);^20−22^ this spin polarization is comparable with that obtained with organic–inorganic hybrid systems^23−25^ and with supramolecular wires.^7,8^ Past results suggest almost a linear correlation between the spin polarization values and the length/thickness of the chiral materials.^6,10^ However, in this study, we found that the spin polarization only slightly increased (from 77 to 88%) with varying thickness from 2 to 20 nm (Figure 3f, Table S2). The weak dependence on film thickness may indicate that the electron was efficiently polarized by the trefoil knot molecules; thus, the competition between spin polarization and spin depolarization reached the equilibrium within a thickness range from 10 to 15 nm.

However, one should realize that the spin polarization does not decay according to thickness, meaning that any randomization of the spin due to scattering is “corrected” by the chiral potential. It is interesting to note that the currents, shown in Figure 3b,c, are very high compared with those obtained in the past for chiral molecules. For example, at a potential of 1.5 V, it is higher by 2 orders of magnitude compared to the current measured for DNA, oligopeptides,^10^ or helicenes^12,13^ at the same potential. The high conductivity may result from the interaction of the conducted electrons with a large number of conjugated components in the structure. It was realized before that the motion of an electron through a helical potential requires exchanging momentum with the system. For saturated hydrocarbons and for systems with low frequency vibrational modes that have angular momentum, the exchange of momentum occurs via electron–vibration interaction, namely, breaking the Born–Oppenheimer approximation.^26−28^ For topological systems that are more “rigid” and “compact”, as the present molecules, these low frequency modes are missing, and the momentum must be transferred between the conducted unbound electron and the bound electrons that are delocalized in the molecule; hence, a more efficient conduction process is expected because of the better match between the masses of the interacting species. This mechanism should result in a weak temperature dependence of the conduction, as was indeed observed, as shown below. However, detailed calculations are required to verify the proposed mechanism.

**Figure 4:**
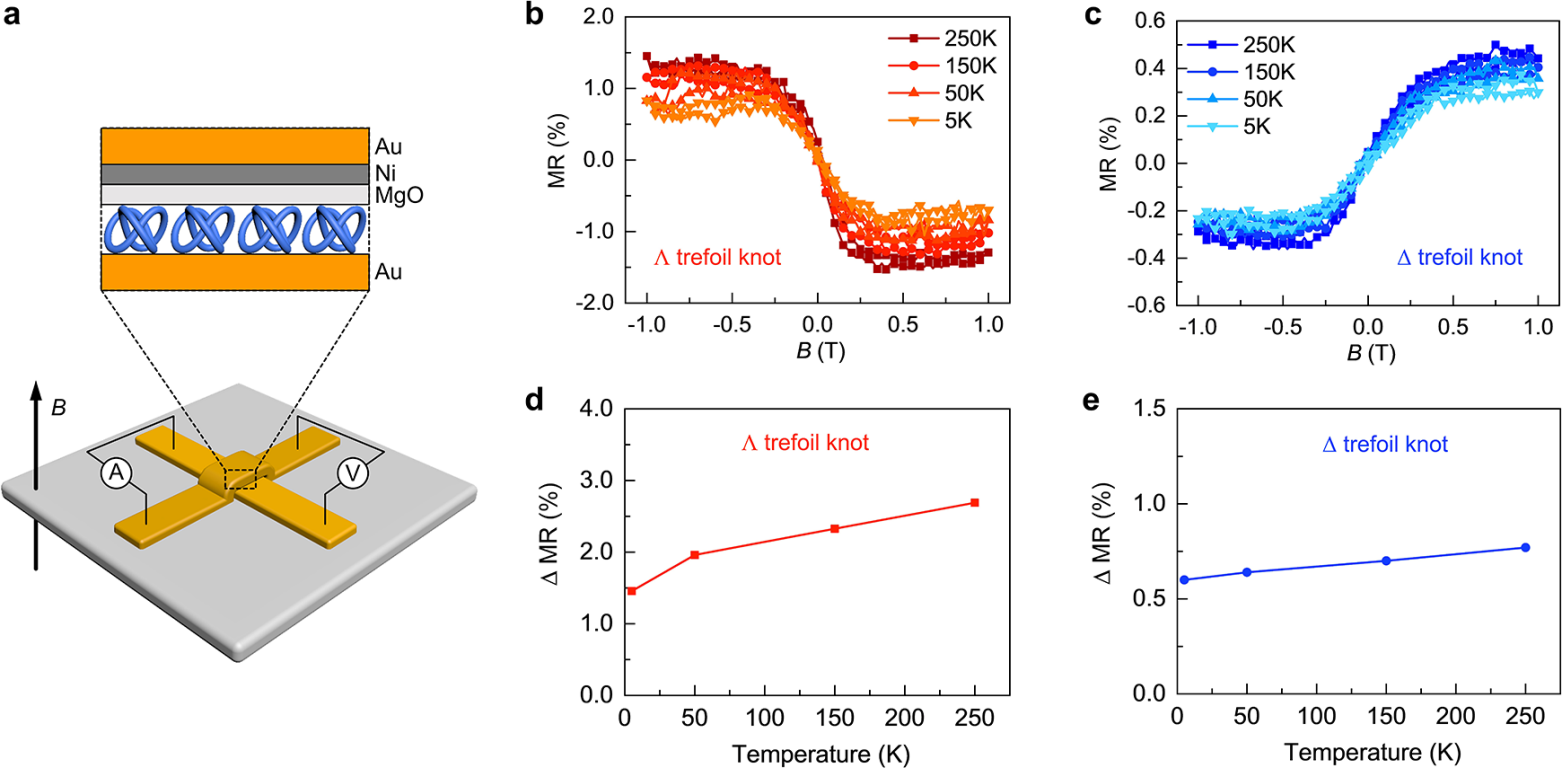
MR response of spintronic devices based on topological molecular knots. (a) Schematic illustration of the cross-bar tunnel junction device. (b,c) MR curves for ∧ and Δ trefoil knot thin films, as a function of the magnetic field between −1.0 and 1.0 T at different temperatures. The measurements were performed at a constant current of 50 μA. (d,e) ΔMR values as a function of temperature, where ΔMR (%) = |MR (%)|_–1.0T_ + |MR (%)|_+1.0T_.

The spin-selective transport process was also studied in spin-valve devices, using a single ferromagnetic electrode instead of two ferromagnetic electrodes as in more traditional spin valves. The ferromagnetic electrode (Ni, 40 nm) and the nonmagnetic electrode (Au, 50 nm) are separated by a trefoil knot thin film and a thin layer of MgO (1.5 nm, as the buffer layer), as schematically illustrated in Figure 4a (see more details in the Methods section). The device resistance was measured under an out-of-plane external magnetic field ranging from −1.0 to 1.0 T. The resistance is plotted as a function of the magnetic field, and the magnetoresistance (MR) of the devices, based on ∧ or Δ molecular trefoil knot thin film, is shown in Figure 4b. The MR is defined as
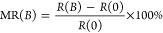
where *R*(0) and *R*(B) are the resistance at zero field and a specific magnetic field, *B*, respectively. The MR response exhibits a typical asymmetric nature as a function of the magnetic field, as observed in previous studies of chiral molecular systems.^29^ The ∧ and Δ trefoil knot–based devices show an opposite MR response due to their opposite chirality (Figure 4b,c), demonstrating that electron transport through the trefoil knot thin film is spin polarized. Compared with the mCP-AFM measurements, the relatively low MR response as well as the slightly different MR intensities for these two enantiomers can be explained by the large area of the electrodes (100 μm^2^); these results in collecting electrons that were either passed through pin holes in the chiral layer or were scattered.

However, these problems do not exist in the mCP-AFM configuration, where conduction at the nanoscale is probed; hence, much higher spin selectivity was observed.^30^

The MR devices can provide insights into temperature-dependent spin transport through topologically chiral structures. As shown in Figure 4b,c, the MR increases slightly with increased temperature. In several former studies, the increase in the spin polarization with temperature is more pronounced.^30^ The weak temperature dependence of MR here may result from the rigidity of the knot structure and the lack of very low frequency vibrations compared with oligopeptides and DNA. In addition, electron conduction may be enabled by momentum transfer to the delocalized electrons on the knot, as discussed above. It is important to understand that the small increase in the MR with temperature is substantially different from that observed in traditional spin-valve devices, in which MR typically decreases with increased temperature.^31^ Below, we present calculations that indicate the importance of the electron–electron interaction in this system.

**Figure 5:**
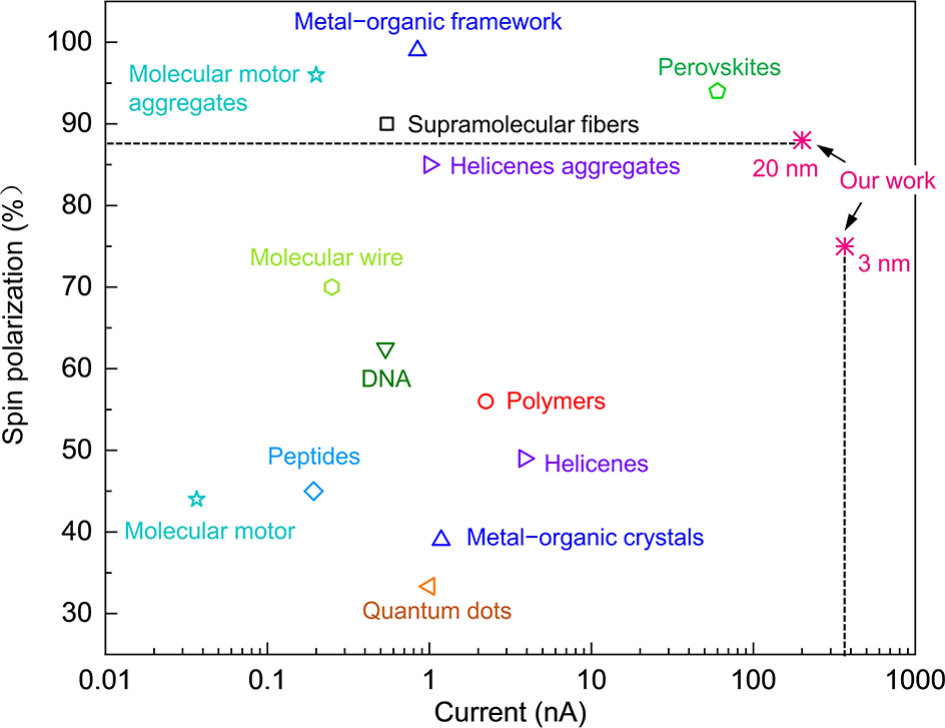
Summary of spin polarization as a function of current in various chiral systems. Spin polarization (%) and the corresponding current (nA, at the bias voltage of 1.5 V) are extracted only from representative chiral systems measured by mCP-AFM. Two points with different thicknesses (3 and 20 nm) from our work are presented here. Whereas the spin polarization increases with the thickness, the current decreases. Only the highest spin polarization and their corresponding current are presented for each system. More information is provided in the Supporting Information (Figure S11 and Table S3). Data extracted for the molecular motor are from ref (32); for molecular motor aggregates, see ref (33); for peptides and DNA, see ref (10); for a molecular wire, see ref (34); for supramolecular fibers, see ref (7); for a metal–organic framework, see ref (35); for helicenes, see ref (12); for helicenes aggregates, see ref (13); for quantum dots, see ref (23); for metal–organic crystals, see ref (36); for polymers, see ref (37); and for perovskites, see ref (38).

In previous studies, the evaluation of chiral systems as spin injectors focused predominantly on the value of spin polarization, and indeed, chiral systems were found with spin polarization values that approached 100%.^35^ However, in order to obtain an efficient spin injector, the conductivity is also important. This parameter is usually not discussed. Therefore, herein, we present the data obtained so far in a two-dimensional presentation in which the scales are the spin polarization and the current (Figure 5). Namely, the figure of merit, FoM, for chiral systems as the spin injector is the product of spin polarization and current, FoM (*V*) = SP(*V*)·*I*(*V*), where SP is the spin polarization and *I* is the current. Figure 5 summarizes the spin polarizations of recently reported chiral materials as a function of the current based on measurements performed with mCP-AFM measurements. The trefoil knots have the highest spin polarization (up to 88%) among all the small molecules, and only a few supramolecular structures have a higher spin polarization value. Moreover, the trefoil knots exhibit about 400 nA at the bias voltage of 1.5 V with an FoM (1.5 V) of 275 nA, which, to the best of our knowledge, is the highest value obtained for a chiral system (Figures 5 and S11 and Table S3). In addition, the molecular knots show spin selectivity with negligible degradation even after heating at 350 °C for 2 h in the air (Figures S12 and S13), hence, the molecular trefoil knots act as a stable spin filter.

The weak temperature dependence of the CISS effect suggests that the electronic correlations that govern the strong asymmetry between the two configurations are mainly of electron–electron interaction character. This can be contrasted by the strongly temperature-dependent CISS effect that can be effectively modeled in terms of vibrationally assisted electron correlations.^30^ The topological aspect of the molecules means that there are no low frequency vibrational modes, in the knot-like molecules, that have angular momentum. Hence, the question is whether there is another way in which the electrons can exchange momentum with the molecular system. Therefore, here, we employ the model for a helical chain of sites, which was introduced in ref (30), though modifying the interactions by also including nearest neighbor exchange (*J*) interactions. These interactions can be represented by terms like *J****s***_m_·**s**_m±1_, in the model Hamiltonian, where *s*_m_ = ψ_m_^†^**σ**ψ_m_/2 denote the total charge and spin associated with the site m in terms of the electron spinor ψ_m_ = (ψ_m↑_ψ_m↓_)^*t*^ and vector of Pauli spin matrices **σ**.

The molecule is mounted in the junction between a ferromagnetic metallic lead and a nonmagnetic metallic lead, parametrized by the couplings **Γ**^L^ = Γ_0_ (σ^0^ + *p*_L_ σ^z^)/2 and **Γ**^R^ = Γ_0_ σ^0^/2 (σ^0^ is the two 2 × 2 unit matrix), and the nonequilibrium electronic structure is calculated through the self-consistent procedure outlined in ref (39).

In order to show the effect of the introduced spin–spin interaction, we use a four-site long helical chain, representing the smallest possible chiral configuration. The molecule is assumed to be strongly coupled to the leads (Γ_0_ = 2 eV), and the spin polarization of the injected electrons is *p*_L_ = 0.2. The on-site energy level ε_0_ = −1 eV and Coulomb repulsion *U* = 10 meV, the nearest neighbor exchange interaction *J* = −0.2 eV and hopping rate *t* = 0.2 eV, and the next-nearest neighbor spin–orbit interaction λ = 1 meV characterize the properties of the molecule.

**Figure 6:**
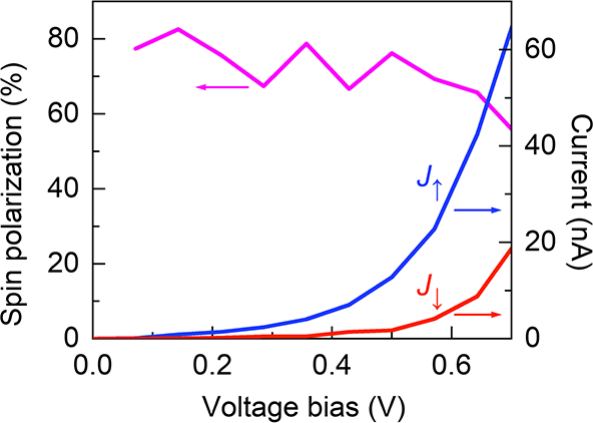
Results from the calculations. The spin polarization (left *y*-axis, pink) is shown as a function of the bias voltage as well as the current (right *y*-axis) for the magnet pointing up (*J*_↑_, blue) and down (*J*_↓_, red).

A typical result of the simulations is shown in Figure 6, showing the current–voltage characteristics for the two configurations signified by *J*_σ_ (σ = ↑,↓), where σ refers to the spin polarization of the charge current injected from the ferromagnetic lead. The currents for the two configurations are conspicuously distinct, something which is also corroborated by the large spin polarization, Figure 6. The results of the calculations are consistent with the data observed and indicate the possible contribution of electron–electron scattering in the spin transport in this system.

We comment that although the four-site long chain is not formed as a knot, it manifests the weak temperature dependence of the chiral molecule, which is the main purpose here. This simplification is justified by the universal properties of the CISS effect that has been reported in an abundance of different molecules, though where the one physical distinctive property of the molecules is chirality. In this sense, our model should be equally applicable to other types of chiral molecules, e.g., oligopeptides, helicene, and DNA.

## Conclusions

Topologically chiral knot molecules are a new class of chiral-induced spin filtering materials. They are unique in showing a combination of properties, high spin filtering, high conductivity, and high thermal stability. The intrinsic chirality was demonstrated by CD spectra, both in solution and as a thin film. The spin filtering measurements were performed with mCP-AFM, which showed a spin polarization of up to 88%, and the MR studies showed a stable signal as a function of temperature.

Utilizing the topologically chiral structures allows us to introduce conjugated elements in the knot, which may cause the large conductivity and large spin polarization and introduce the possibility of electron–electron interactions and as a result the thermal stability. The calculations indicate that indeed this electron–electron interaction can be responsible for high spin selectivity and high conductivity. Hence, the chiral knot molecules, because of their structure, can serve as a new class of spintronics elements.

## Supporting Information Available

Synthetic procedures, characterization data for the compound, and extended experiment (PDF)
Supplementary crystallographic data (ZIP)

## Accession Codes

CCDC 2234794 contains the supplementary crystallographic data for this paper. These data can be obtained free of charge via www.ccdc.cam.ac.uk/data_request/cif, or by emailing data_request@ccdc.cam.ac.uk, or by contacting The Cambridge Crystallographic Data Centre, 12 Union Road, Cambridge CB2 1EZ, UK; fax: +44 1223 336033.

## Notes

Details are provided in the Supporting Information and are also available from the authors upon reasonable request.
