# Supplementary material for: Highly Conductive Topologically Chiral Molecular Knots as Efficient Spin Filters
Source: J Am Chem Soc. 2023 Nov 16;145(49):26791–8. doi: 10.1021/jacs.3c08966 (PMC10722505; doi:10.1021/jacs.3c08966)
Supplement: Supplementary file 1 — ja3c08966_si_001.pdf [file ja3c08966_si_001.pdf]

Supporting information for

# Highly conductive topologically chiral molecular knots as efficient spin filters

Dan-Yang Zhang, Yutao Sang*, Tapan Kumar Das, Zhao Guan, Ni Zhong, Chun-Gang Duan, Wei Wang*, Jonas Fransson*, Ron Naaman*, and Hai-Bo Yang*

**Corresponding Authors**

* hbyang@chem.ecnu.edu.cn (H.-B. Yang)
* ron.naaman@weizmann.ac.il (R. Naaman)
* jonas.fransson@physics.uu.se (J. Fransson)
* wwang@chem.ecnu.edu.cn (W. Wang)
* sangyt@fudan.edu.cn (Y. Sang)

## Materials and General Methods

All reagents were commercially available and used as supplied without further purification. Deuterated solvents were purchased from Cambridge Isotope Laboratory (Andover, MA). All solvents were dried according to standard procedures and all of them were degassed under $N_2$ for 30 minutes before use. All air-sensitive reactions were carried out under an inert $N_2$ atmosphere.

The synthesis of the molecular trefoil knots was adapted from literature reports.[1-2] $^1$H NMR spectra were recorded on a Bruker 500 MHz Spectrometer at 298 K. The $^1$H chemical shifts are reported relative to the residual solvent signals. The MALDI MS experiments were carried out on a Shimadzu Axima Performance MALDI TOF/TOF Mass Spectrometer equipped with a 337 nm nitrogen laser. The instrument was operated in positive ion reflection mode and the accelerating voltage was 20 kV. Trans-2-[3-(4-tert-Butylphenyl)-2-methyl-2-propenylidene]malononitrile (DCTB) matrix, dissolved in $CH_2Cl_2$ at 20 mg/mL, was used as the matrix. The samples were dissolved in $CH_2Cl_2$ at 5−10 mg/mL. Chiral separation was performed using a Shimadzu HPLC System (LC-20AR) equipped with a CHIRALPAK IF® column (250 mm L × 10 mm I. D. for separation); the samples were eluted with DCM/hexane/isopropanol (10/15/2, V/V/V) at flow rates of 3.0 mL/min.

### Circular dichroism (CD) measurements

Circular dichroism (CD) spectra were measured on a Chirascan Series Spectrometer (Applied Photo Physics Ltd, UK) at room temperature. A quartz cuvette with an optical pathway of 2 mm was used for the solution-state samples. For the solid-state measurements, thin films were prepared by spin coating the molecules on a quartz plate (1 mm thick). They were placed perpendicular to the light path of the CD spectrometer and rotated within the cuvette plane to rule out the possibility of the birefringency phenomenon and to eliminate the possible angle dependence of the CD signal. The measurement conditions for all spectra were at a scan range of 200 to 400 nm, 0.5 s time per point, 1-nm step size, and a 1-nm bandwidth.

### Polarization-modulation infrared reflection absorption spectroscopy (PM-IRRAS) measurements

PM-IRRAS was used to characterize the trefoil knot thin films on the electrodes. They were performed at room temperature under positive nitrogen pressure in a reflection-absorption cell (Harrick, Inc.) with a PM-FTIR spectrometer (PMA-50 coupled to

Vertex 70, Bruker). Two thousand scans were performed for each spectrum with a resolution of 4 $cm^{-1}$ using a mercury-cadmium-telluride (MCT) detector. All the samples were mounted at a Brewster angle of 80°.

**AFM measurements**

The AFM topography images were obtained on a Dimension FastScan, using ScanAsyst mode under ambient conditions. A silicon cantilever (spring constant, ~42 N/m) at a resonant frequency of 200 to 400 kHz was used for all the morphology characterizations.

**mCP-AFM measurements**

Conductive-AFM results were performed using the PF-TUNA mode (Bruker) under ambient conditions. Here, trefoil knot films were prepared on freshly cleaved HOPG substrates by spin coating the molecules at different concentrations. The thin films were annealed under 60°C for several hours before AFM measurement. Current-voltage (*I-V*) spectroscopy measurements were recorded by performing voltage ramps with the tip in contact with the surface. Co-Cr–coated tips (MESP-V2, Bruker, with spring constant 3 $Nm^{-1}$) were pre-magnetized by a strong permanent magnet (~0.5 T) for >30 min and then used for the scan immediately. If the measurement time was longer than 60 min, the tip was placed back to the same pole of the magnetic field and magnetized for 30 min again. *I-V* curves were acquired by ramping the voltage from −1.5 to +1.5 V, with a frequency of 0.5 Hz. At least 50 *I–V* traces were recorded and averaged for each location and magnetic field orientation. During the measurement, the gain value was 10. All the recorded traces were provided in the supporting information. For each *I–V* measurement, the tip was placed in a new location, and movement between points was done with the tip lifted from the surface to prevent damage to the sample. At least three locations were measured for each sample.

**Device fabrication and magnetoresistance (MR) measurements**

MR measurements were performed in a crossbar geometry on a $SiO_2$ substrate. The bottom electrode had a 2 μm width, consisting of 8 nm titanium (Ti) as an adhesive layer and 40 nm gold (Au), respectively, fabricated by optical lithography. Prior to preparing the chiral thin films, the devices were cleaned in boiling acetone and boiling ethanol for 10 min each. Afterwards, they were kept in a UV ozone plasma cleaner for 15 min to remove all organic residues. The trefoil knot thin film was spin-coated on the

pre-treated devices and dried overnight in a vacuum. On top of the thin molecular film, insulating buffer layers of 1.5 nm magnesium oxide (MgO) were grown by e-beam evaporation and then the final top electrode, composed of Ni and Au with a thickness of 40 and 20 nm, respectively, was evaporated using a shadow mask with a line width of 50 μm. All electrical MR measurements were carried out within the cryogenic system made by Cryogenics, Ltd. A magnetic field of up to 1.0 T was applied perpendicular to the sample plane and the resistance of the device was measured using the standard four-probe method. A constant current of 0.05 mA was applied using a Keithley current source (model 2400) and the voltage across the junction was measured using a Keithley nanovoltmeter (model 2182A).

**Stability measurements**

We first performed the thermal gravimetric analyses (TGA) on the molecular knots. A SHIMADZU DTG-60 thermal analyzer was employed to obtain the TGA traces heated at a rate of 10 °C $min^{-1}$ to 700 °C under airflow. Based on the TGA result, we chose 350 °C as the temperature to check the stability. The molecular knot thin film on HOPG was heated at 350 °C for 2 hours in the air. The mCP-AFM measurements of the same sample were performed before and after the heating process.

## Synthesis and Characterization of the molecular trefoil knots

**Scheme S1.** Synthetic route of the molecular trefoil knots.

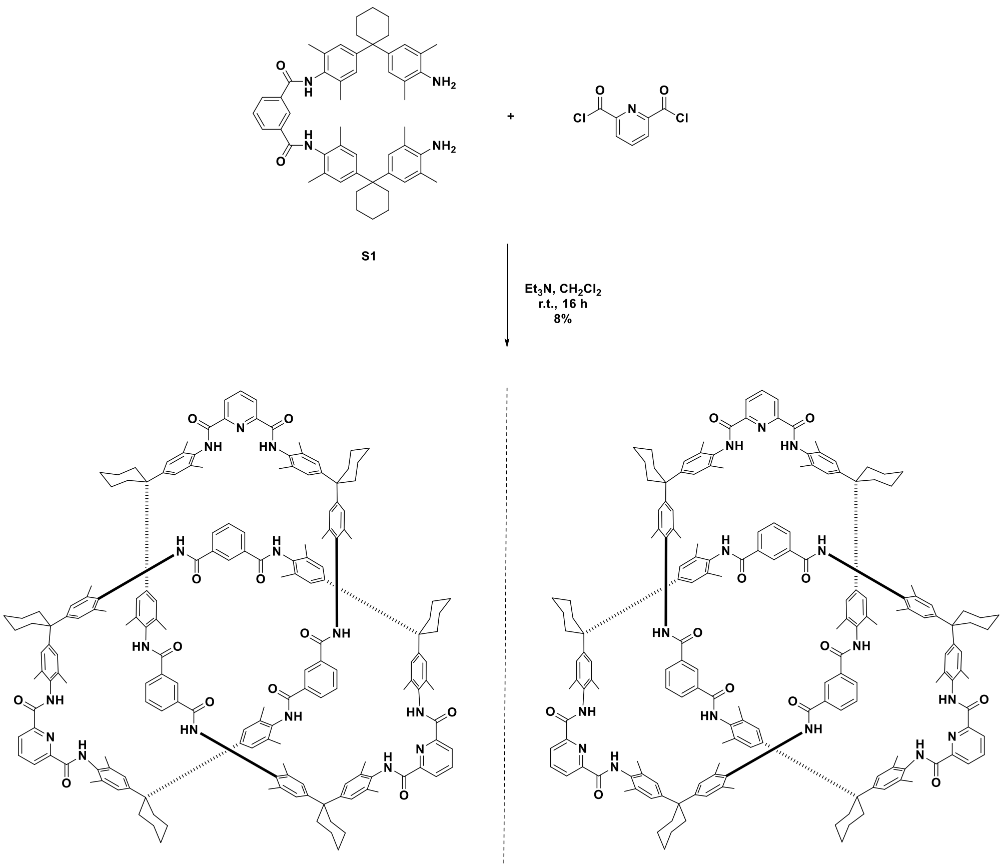


The trefoil knots were synthesized as a racemic mixture and then separated by a high-pressure liquid chromatography (HPLC) column (see below).[1, 2] Typically, a solution of 2,6-pyridinedicarbonyl chloride (112 mg, 0.5 mmol) in dry dichloromethane (50 mL) and a solution of amine **S1** (388 mg, 0.5 mmol) in dry dichloromethane (200 mL) with $Et_3N$ (0.2 mL) were added simultaneously (12.5 mL/h) into a stirred flask containing dry dichloromethane (500 mL). After 4 h, the addition was complete and the reaction mixture was stirred for another 12 h. The solvent was removed under reduced pressure and the crude products were purified by chromatography on silica gel with dichloromethane/methanol (200:3, v/v). Crude products were then purified by gel permeation chromatography (GPC). A pale-yellow solid (35 mg, 8% yield) was obtained. Only well-resolved signals are referenced below. $^1$H NMR ($d_6$-DMSO, 500 MHz, 298 K): $\delta$ 11.01 (s, 1H), 10.97 (s, 1H), 10.56 (s, 1H), 10.51 (s, 1H), 10.17 (s, 1H), 9.78 (s, 1H), 9.73 (s, 1H), 9.49 (s, 1H), 9.32 (s, 1H), 9.12 (s, 1H), 8.99 (s, 1H), 8.58 (s, 1H), 8.27-6.33 (m, 45H), 2.24-1.14 (m, 132H). LRMS (MALDI-TOF): Calculated for $[C_{177}H_{189}N_{15}O_{12}+ H]^+$: 2719.6; found: 2719.2.

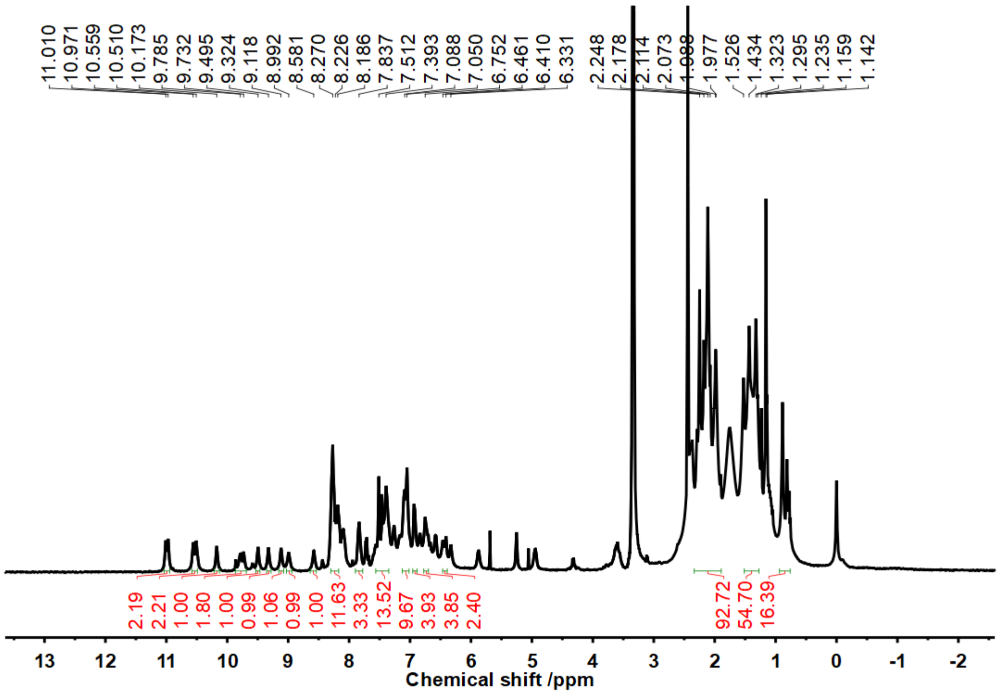


**Figure S1**. $^{1}$H NMR spectrum ($CD_2Cl_2$, 298 K, 500 MHz) of the molecular trefoil knot.

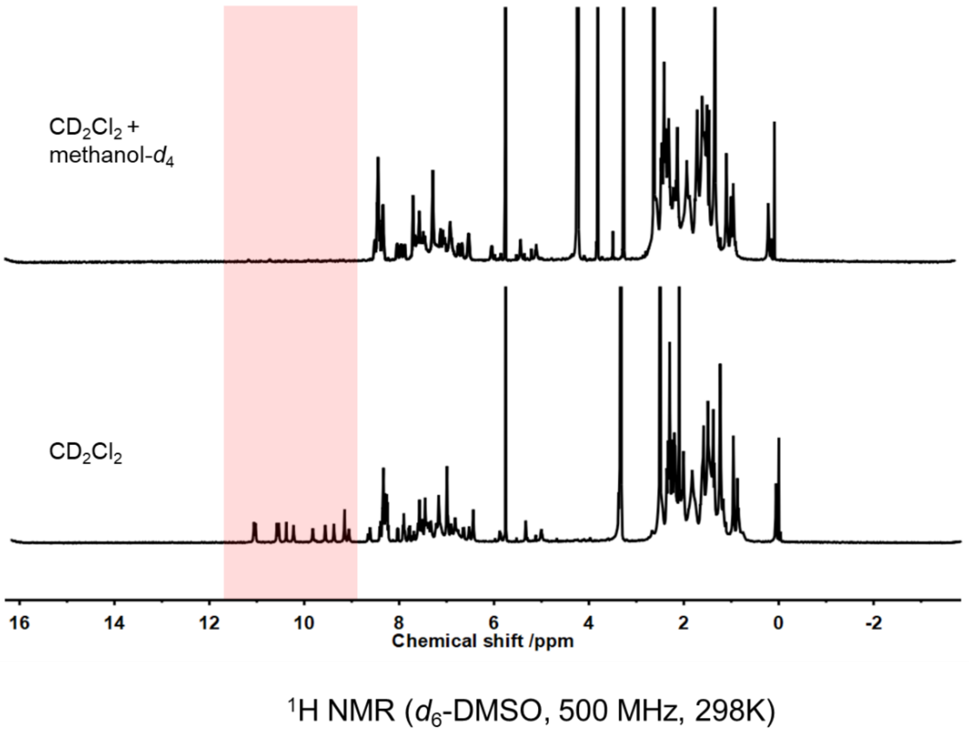


**Figure S2.** $^{1}$H NMR spectra (298 K, 500 MHz) of molecular trefoil knot measured in $CD_2Cl_2$ (*bottom*) and in $CD_2Cl_2$+methanol-$d_4$ (*top*), indicating the H/D exchange of 12 amide protons (the red area) upon the addition of methanol-$d_4$.

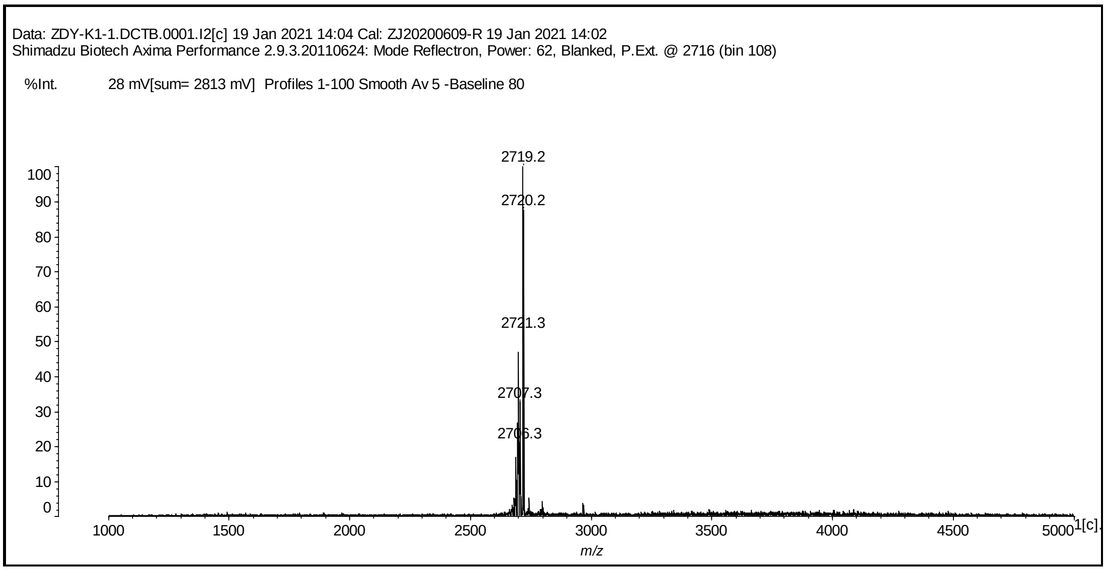


**Figure S3.** MALDI-TOF-MS spectrum of the molecular trefoil knot. The peak of *m/z* = 2719.2 was observed, which was in agreement with the theoretical value of $[M + H]^+$ (*m/z* = 2719.6).

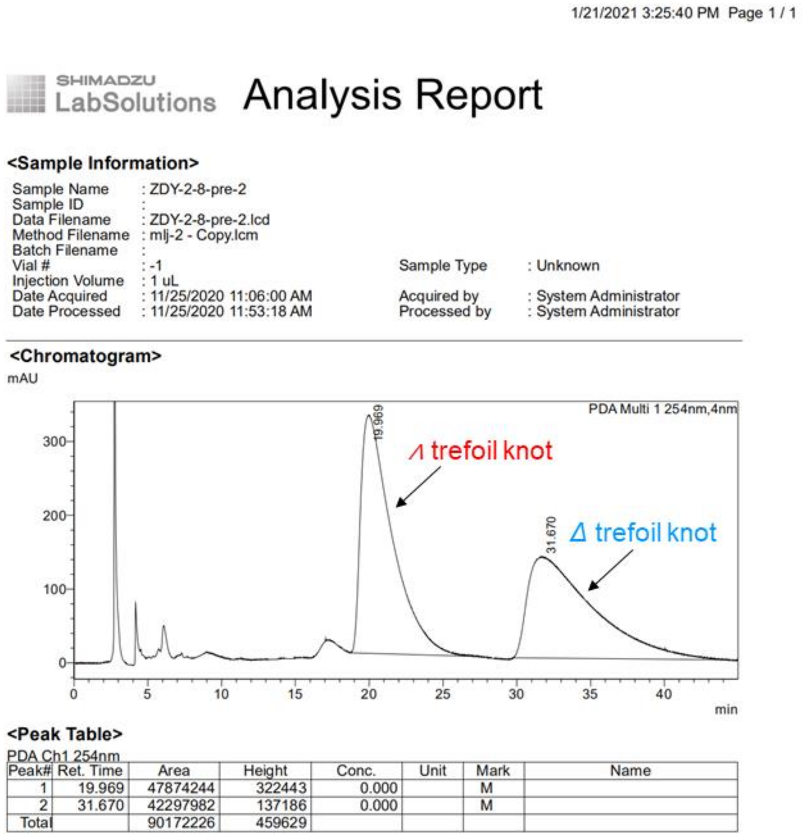
1/21/2021 3:25:40 PM Page 1 / 1

SHIMADZU LabSolutions **Analysis Report**

**<Sample Information>**

Sample Name : ZDY-2-8-pre-2
Sample ID :
Data Filename : ZDY-2-8-pre-2.lcd
Method Filename : mlj-2 - Copy.lcm
Batch Filename :
Vial # : -1
Sample Type : Unknown
Injection Volume : 1 uL
Date Acquired : 11/25/2020 11:06:00 AM
Acquired by : System Administrator
Date Processed : 11/25/2020 11:53:18 AM
Processed by : System Administrator

**<Chromatogram>**



**<Peak Table>**

PDA Ch1 254nm

| Peak# | Ret. Time | Area | Height | Conc. | Unit | Mark | Name |
|---|---|---|---|---|---|---|---|
| 1 | 19.969 | 47874244 | 322443 | 0.000 | | M | |
| 2 | 31.670 | 42297982 | 137186 | 0.000 | | M | |
| Total | | 90172226 | 459629 | | | | |

**Figure S4**. Chiral HPLC preparation [column, CHIRALPAK IF® column (250 mm L × 10 mm I. D.); eluent, 8% diethylamine in DCM/hexane (2:3); flow rate, 3 mL/ min; detection, 254 nm] of the racemic mixture of molecular knots.

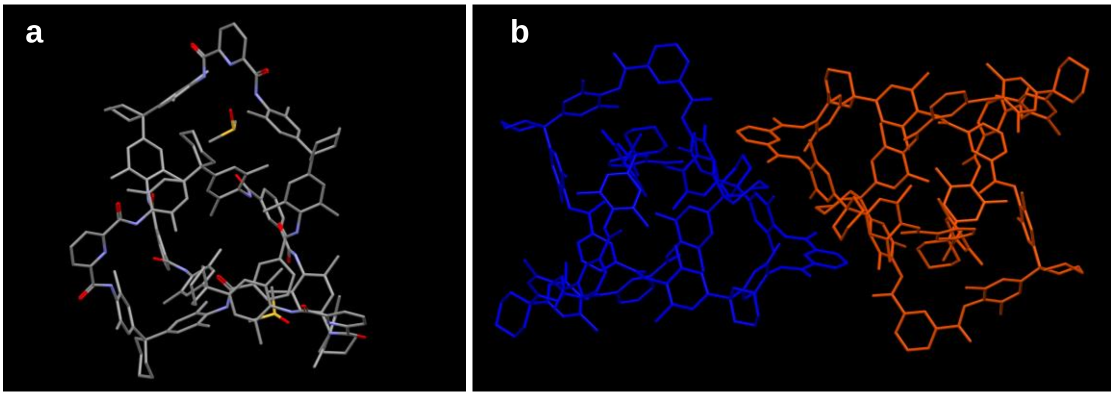


**Figure S5.** (a) Monomer and (b) enantiomers of the crystal structure of the molecular trefoil knots obtained from the racemic mixture in DMSO/$CH_3OH$ (blue: *Δ* trefoil knot; orange: *Λ* trefoil knot). C gray, O red, N purple, S yellow. All the hydrogen atom were omitted for clarity. The X-ray crystallographic coordinates for structure have been deposited at the Cambridge Crystallographic Data Centre (CCDC), under deposition numbers CCDC 2234794.

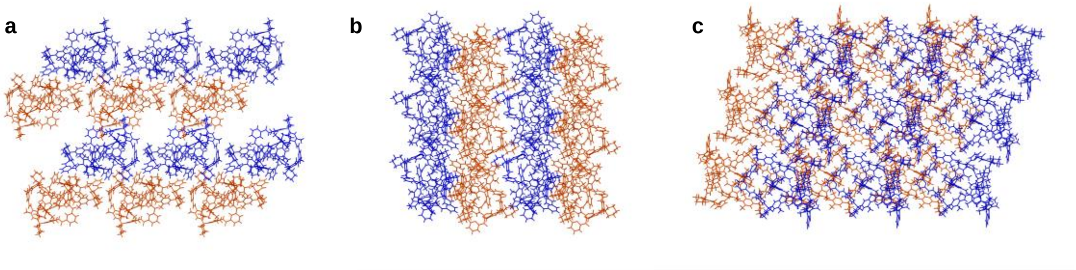


**Figure S6.** Crystal packing of enantiomers (a) from *a* axis; (b) from *b* axis and (c) from *c* axis. The blue and red structure represented *Δ* trefoil knot and *Λ* trefoil knot respectively and all the hydrogen atoms were omitted for clarity.

It should be noted that the single crystals were obtained in DMSO/$CH_3OH$; thus, the absolute configuration and crystal structures differed from the reported data obtained in $CHCl_3$/$CH_3OH$ (ref 2). Table S1 compares the crystallographic structures obtained in these two mixed solvents (see below).

**Table S1.** Crystallographic data obtained in different solvent.

| Name | Our crystal obtained in DMSO/$CH_3OH$ | Vögtle' crystal obtained in $CHCl_3$/$CH_3OH$ (ref. 2) |
|---|---|---|
| Empirical formula | $C_{193}H_{237}N_{15}O_{20}S_8$ | $C_{44.25}H_{47.25}N_{3.75}O_3$ |
| Formula weight | 3343.44 | 3900.1 |
| Temperature/K | 236(90) | 123(2) |
| Crystal system | triclinic | monoclinic |
| Space group | P-1 | $P2_1/n$ |
| a/Å | 18.2626(3) | 20.504(1) |
| b/Å | 24.9409(4) | 39.813(1) |
| c/Å | 28.3395(3) | 25.155(1) |
| α/° | 78.2060(10) | 90 |
| β/° | 86.9020(10) | 94.47(1) |
| γ/° | 71.0290(10) | 90 |
| Volume/$Å^3$ | 11948.3(3) | 20472.2(14) |
| Z | 2 | 4 |
| $\rho_{calc}g/cm^3$ | 0.929 | 1.265 |
| μ/$mm^{-1}$ | 1.104 | 0.273 |
| F(000) | 3576 | 8280 |
| Crystal size/$mm^3$ | 0.39 × 0.32 × 0.26 | 0.70 × 0.60 × 0.30 |
| Radiation | CuKα (λ = 1.54184) | MoKα (λ = 0.71073) |
| 2Θ range for data collection/° | 7.716 to 134.158 | 5.86 to 50 |
| Index ranges | -21 ≤ h ≤ 21, -29 ≤ k ≤ 29, -33 ≤ l ≤ 23 | -24 ≤ h ≤ 20, -46 ≤ k ≤ 47, -29 ≤ l ≤ 29 |
| Reflections collected | 131446 | 115403 |
| Independent reflections | 42367 [$R_{int}$ = 0.1155, $R_{sigma}$ = 0.0927] | 35794 [$R_{int}$ = 0.1088, $R_{sigma}$ = /] |
| Data/restraints/parameters | 42367/261/2174 | 35794/1058/2359 |
| Goodness-of-fit on $F^2$ | 0.845 | 0.989 |
| Final R indexes [I>=2σ (I)] | $R_1$ = 0.1218, $wR_2$ = 0.2796 | $R_1$ = 0.1767, $wR_2$ = 0.4572 |
| Final R indexes [all data] | $R_1$ = 0.1460, $wR_2$ = 0.2957 | $R_1$ = 0.2102, $wR_2$ = 0.4844 |
| Largest diff. peak/hole / e $Å^{-3}$ | 1.39/-0.85 | 1.58/-1.51 |

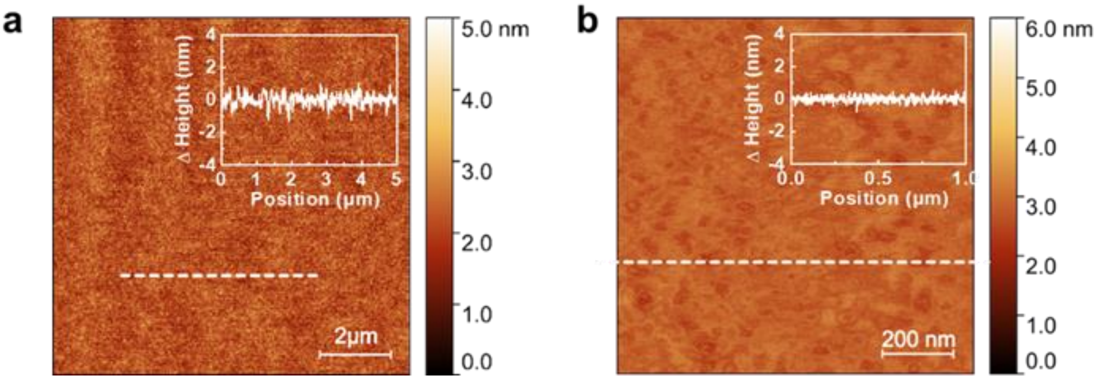


**Figure S7.** AFM topography images of the (a) ⊿ and (b) ⩘ molecular trefoil thin film. Insets: AFM height profile of the white line drawn in the AFM image. Note that the scanning area is different for (a) and (b).

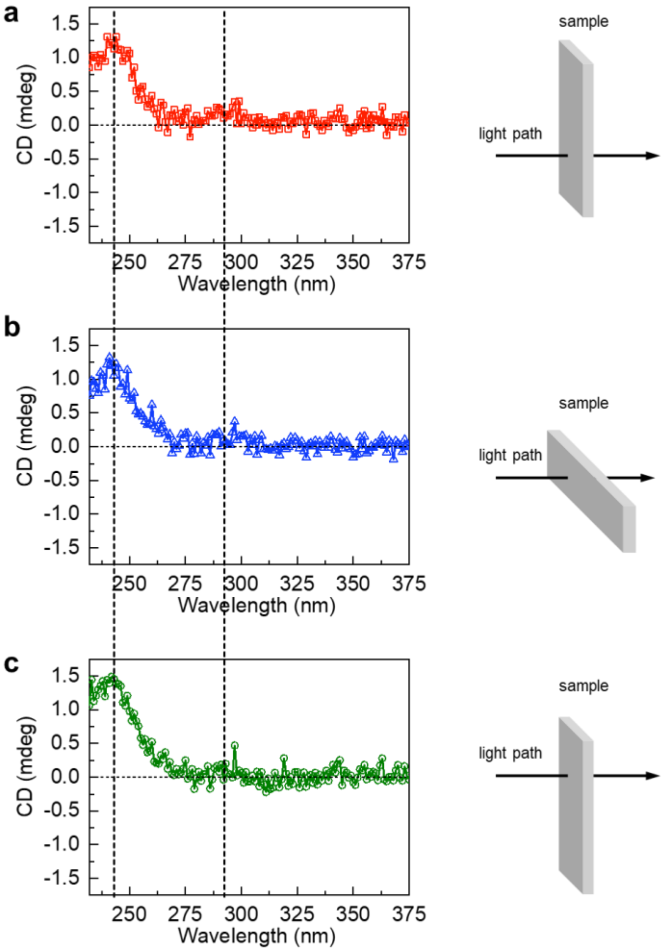


**Figure S8.** CD spectra of ⩘ trefoil knot thin films and corresponding sample placement schematic. Thin films were prepared on a quartz plate (1 mm thick). Then they were placed perpendicular to the light path of a CD spectrometer and measured at different

angles, 0 degree (a), 90 degrees (b) and 180 degrees (c) within the cuvette plane. The CD curves of these thin films were identical when measured from different angles, thus eliminating contamination from linear dichroism.

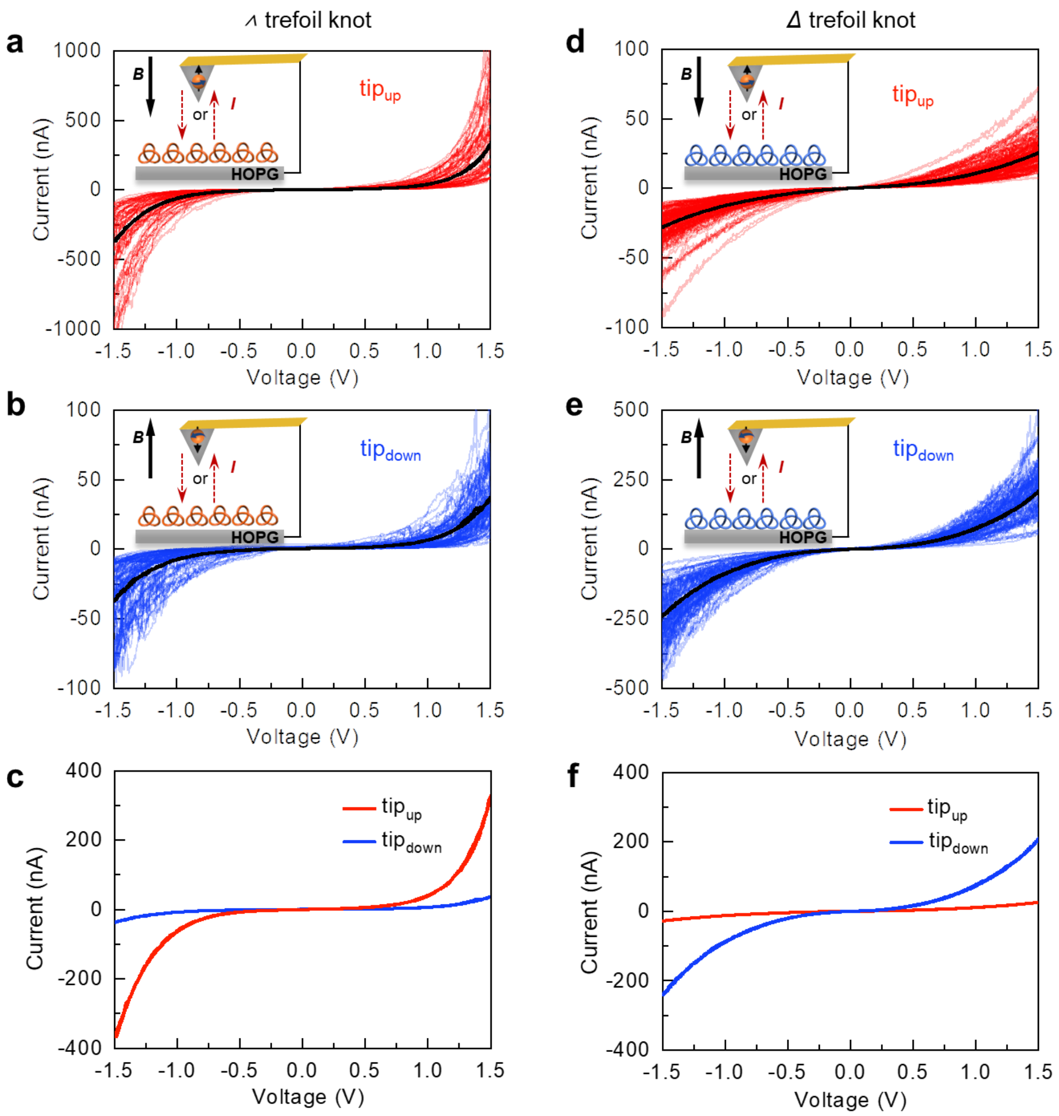


**Figure S9.** Spin-dependent conduction through the *Λ* trefoil knot (**a**-**c**) and *Δ* trefoil knot (**d**-**f**) thin films. Measurements were performed by magnetic-conductive-probe AFM (mCP-AFM) with a ferromagnetic (Co-Cr-coated) tip. The tip can be magnetized by a permanent magnet with different magnetization directions (field-up or field-down with respect to the substrate), and the magnetized tip is subsequently used in the measurements (see the schematic therein). The panels highlight the density of the *I*-*V* characteristics acquired over a -1.5 to +1.5 V potentiometric window. Each line (red and blue) represents one measurement, and the solid black lines represent the average

of *I*-*V* curves. Note the ordinate scales are different in the individual figures to suit each *I*-*V* curves.

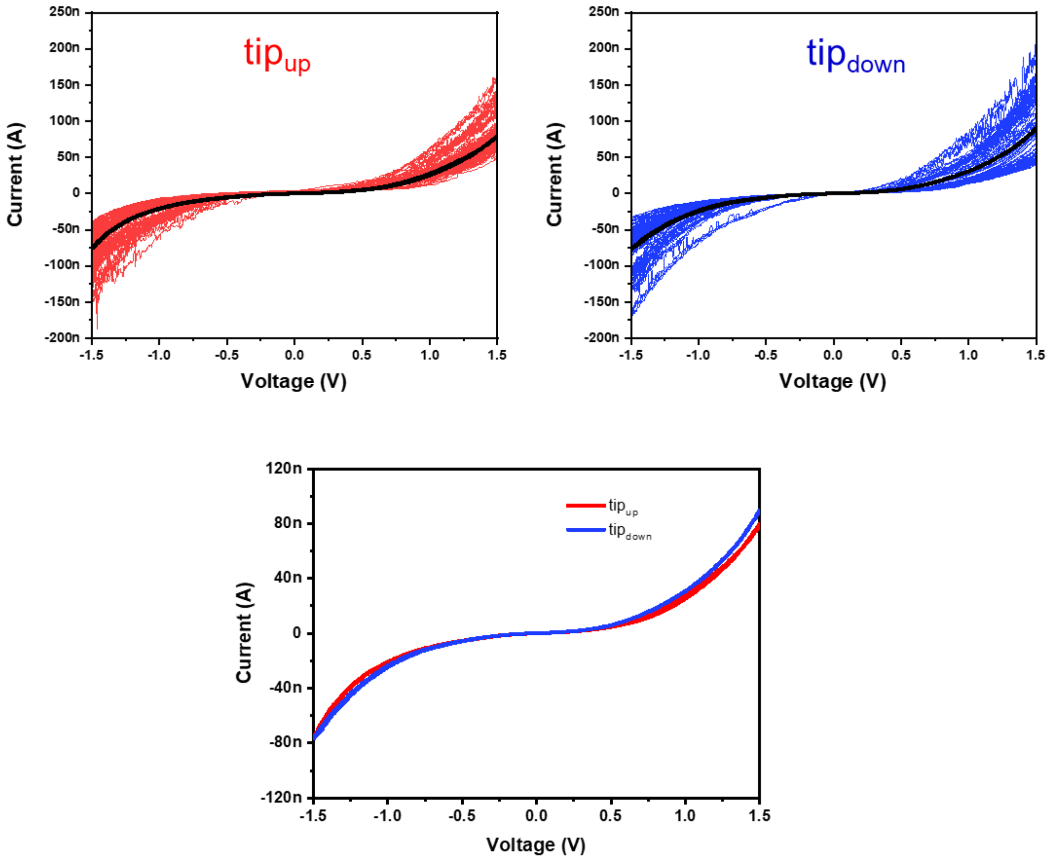


**Figure S10.** Spin-dependent conduction through the racemic trefoil knot thin films. Measurements were performed by magnetic-conductive-probe AFM (mCP-AFM) with a ferromagnetic (Co-Cr-coated) tip. The tip can be magnetized by a permanent magnet with different magnetization directions (field-up or field-down with respect to the substrate), and the magnetized tip is subsequently used in the measurements. The panels highlight the density of the *I-V* characteristics acquired over a -1.5 to +1.5 V potentiometric window. Each line (red and blue) represents one measurement, and the solid black lines represent the average of *I-V* curves.

**Table S2** Film-thickness dependence of spin polarization measured by mCP-AFM

| Thickness (nm) | Spin polarization (%) |
|---|---|
| 2 | 77.3 ± 3.1 |
| 3 | 79.7 ± 2.4 |
| 5 | 82.1 ± 2.5 |
| 10 | 85.4 ± 2.7 |
| 15 | 86.6 ± 2.1 |
| 20 | 87.6 ± 1.9 |

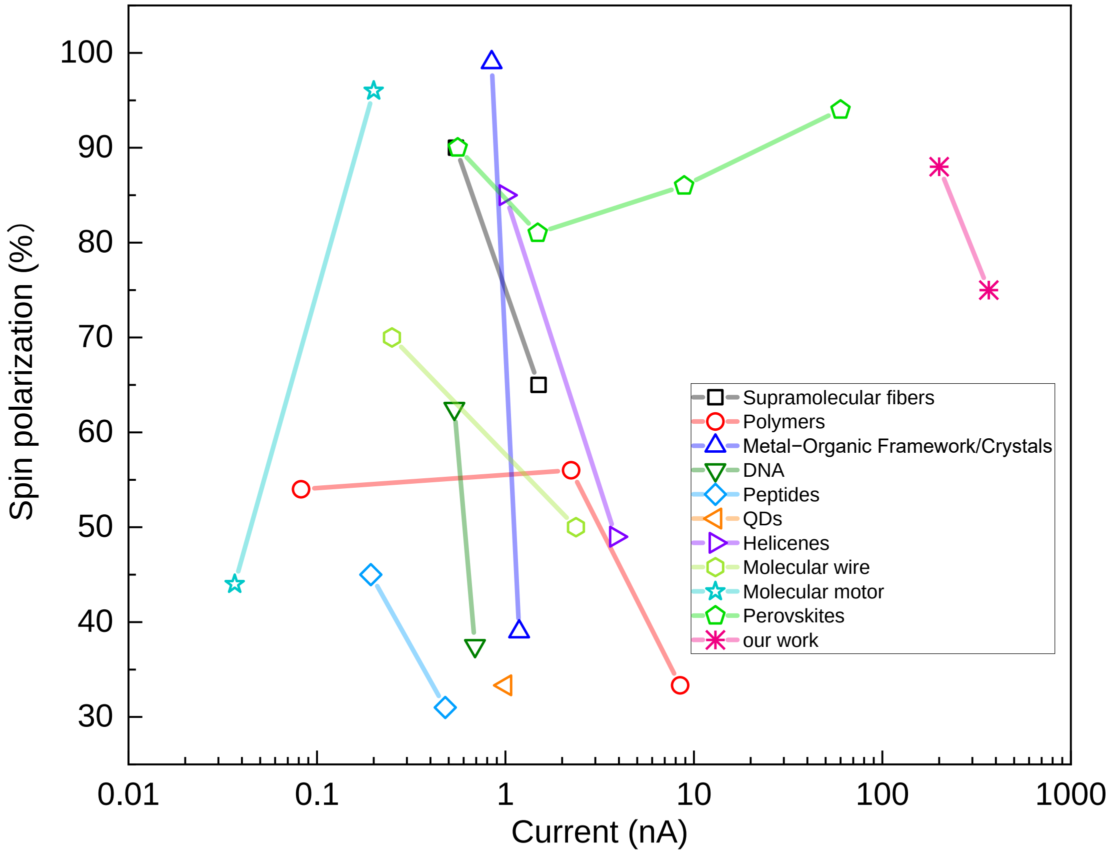


**Figure S11.** Summary of the spin polarization as a function of current in various chiral systems. Spin polarization (%) and the corresponding current (nA, at the bias voltage of 1.5 V) are extracted only from representative chiral systems measured only by mCP-AFM. Note that most kinds of materials have more than one example (points), and they are marked with same colour and are connected by a line. See the corresponding values from **Table S2**. Data were extracted for a molecular motor from refs. [3, 4]; for peptide and DNA from ref. [5]; for a molecular wire from refs. [6, 7]; for supramolecular fibers from refs. [8, 9]; for a metal–organic framework or crystals from refs. [10, 11]; for helicenes from refs. [12, 13]; for quantum dots from ref. [14]; for polymers from refs. [15, 16, 17]; and for perovskites from refs. [18, 19, 20, 21].

**Table S3.** Summary of the spin polarization as a function of current in various chiral systems.

| No. | System investigated | Current (nA) at the bias voltage of 1.5 V | Spin polarization (%) | Ref. |
|---|---|---|---|---|
| 1 | **Molecular motor** | 0.0366 | 44 | [3] |
| 2 | **Molecular motor-formed aggregates** | 0.2 | 96 | [4] |
| 3 | **Oligopeptides**<br>Ala4 | 0.4797 | 31 | [5] |
| 4 | **Oligopeptides**<br>Ala7 | 0.193 | 45 | [5] |
| 5 | **Double-stranded DNA**<br>20bp | 0.69 | 37.5 | [5] |
| 6 | **Double-stranded DNA**<br>50bp | 0.5363 | 62.5 | [5] |
| 7 | **Molecular wire**<br>Conjugated zinc porphyrin + chiral ligands | 0.25 | 70 | [6] |
| 8 | **Molecular wire**<br>Polypeptide + (porphinato)zinc | 2.369 | 50 | [7] |
| 9 | **Supramolecular fibres**<br>Assembled from a tetra-amidated porphyrin with 3,5-dialkoxyphenyl wedges | 0.547 | 90 | [8] |
| 10 | **Supramolecular fibres**<br>Assembled from triphenylene-2,6,10-tricarboxamide with aliphatic side chains | 1.5 | 65 | [9] |
| 11 | **Metal–organic framework** | 0.8455 | 99 | [10] |
| 12 | **Metal–organic crystals** | 1.183 | 39 | [11] |
| 13 | **Helicenes** | 3.855 | 49 | [12] |
| 14 | **Helicene-formed aggregates** | 1 | 85 | [13] |
| 15 | **Quantum dots** | 1 | 33.33 | [14] |
| 16 | **Polymers**<br>poly(phenylacetylene)s | 2.2334 | 56 | [15] |
| 17 | **Polymers**<br>poly(fluorene-co-thiophene) | 8.454 | 33.33 | [16] |
| 18 | **Polymers**<br>poly(2-vinyl pyridine) | 0.0824 | 54 | [17] |
| 19 | **Perovskites**<br>2D tin iodide | 60 | 94 | [18] |
| 20 | **Perovskites**<br>1D Chiral Hybrid Lead-Bromide | 0.558 | 90 | [19] |
| 21 | **Perovskites**<br>2D-layered Pb-iodide | 8.88 | 86 | [20] |
| 22 | **Perovskites**<br>2D-layered lead iodide | 1.4844 | 81 | [21] |

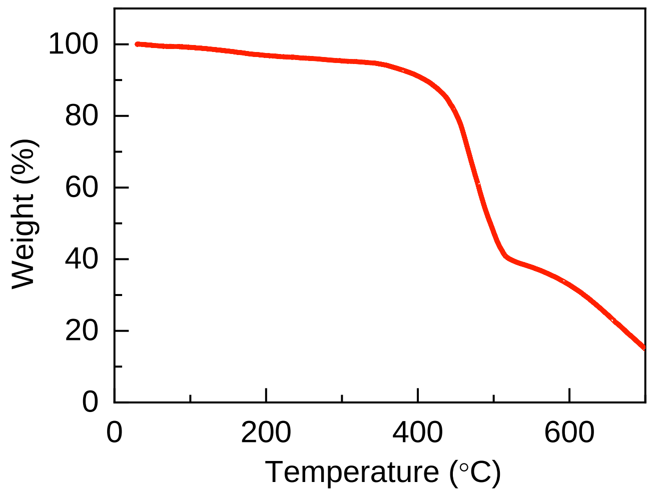


**Figure S12.** Thermal gravimetric analysis (TGA) profile of the molecular trefoil knot.

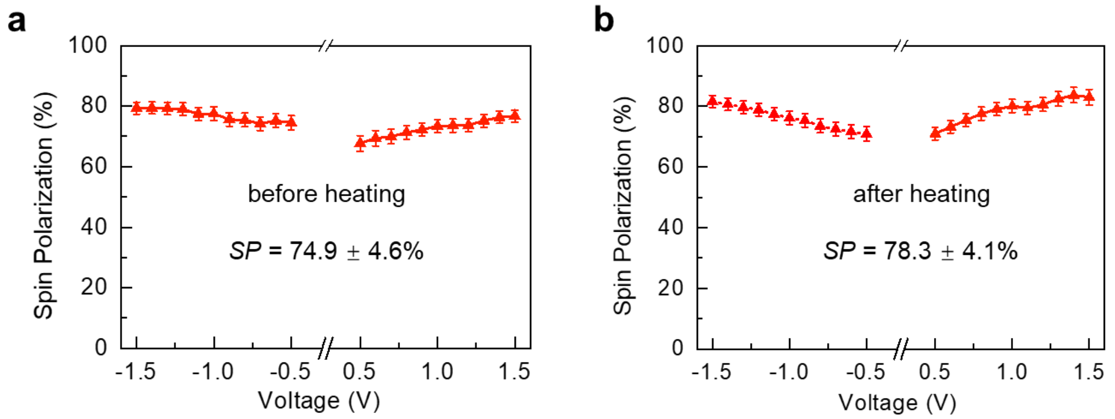


**Figure S13.** Spin polarization as a function of applied bias for $\Lambda$ molecular trefoil knots before (**a**) and after (**b**) heating at 350 °C for 2 hours in the air.

## Theoretical modeling and calculations of the current

The goal with the modeling is to capture how the chiral induced spin selectivity effect may have a weak temperature dependence. In contrast to the effect by electron-phonon interactions, which gives a strong temperature dependence, we address the current observations using electron-electron interactions.

The model of the full set-up comprises metallic leads between which a chiral molecule is mounted. Injection of spin-polarized electrons in the system is provided by assuming that one of the leads is ferromagnetic. A Hamiltonian model for the set-up may be written

$$\mathcal{H} = \mathcal{H}_L + \mathcal{H}_R + \mathcal{H}_T + \mathcal{H}_{mol},$$

where $\mathcal{H}_\chi = \sum_{\boldsymbol{k}\in\chi} \psi_{\boldsymbol{k}}^\dagger \boldsymbol{E}_{\boldsymbol{k}} \psi_{\boldsymbol{k}}$, $\chi = L, R$, describes the electronic structure of the left ($\chi = L$) and right ($\chi = R$) lead in terms of the spinor $\psi_{\boldsymbol{k}} = (\psi_{\boldsymbol{k}\uparrow}\psi_{\boldsymbol{k}\downarrow})^t$ and the electron energy band defined by the $2 \times 2$ matrix $\boldsymbol{E}_{\boldsymbol{k}}$. The tunneling between the leads and the molecule is captured by $\mathcal{H}_T = \sum_{\boldsymbol{k}\in L} \psi_{\boldsymbol{k}}^\dagger \boldsymbol{L}_{\boldsymbol{k}} \psi_1 + \sum_{\boldsymbol{k}\in R} \psi_{\boldsymbol{k}}^\dagger \boldsymbol{R}_{\boldsymbol{k}} \psi_{\mathbb{M}} + H.c.$, where $\boldsymbol{L}_{\boldsymbol{k}}$ ($\boldsymbol{R}_{\boldsymbol{k}}$) denote the spin-dependent tunneling matrix elements between the left (right) lead and the first (last) site in the molecule. The molecule, finally, is modeled by

$$\mathcal{H}_{mol} = \sum_{m=1}^{\mathbb{M}} \left(\epsilon_m \psi_m^\dagger \psi_m + U n_{m\uparrow} n_{m\downarrow}\right) - t \sum_{m=1}^{\mathbb{M}-1} \left(\psi_m^\dagger \psi_{m+1} + H.c.\right) + \lambda \sum_{m=1}^{\mathbb{M}-2} \left(i \psi_m^\dagger \boldsymbol{v}_m^{(+)} \cdot \boldsymbol{\sigma} \psi_{m+2} + H.c.\right)$$
$$+ \sum_{m=1}^{\mathbb{M}-1} ((U' - J/2) n_m n_{m+1} - 2J \boldsymbol{s}_m \cdot \boldsymbol{s}_{m+1})$$

Here, the first term defines the energy at each molecules site $m \in \{1,2, \ldots, \mathbb{M}\}$ in terms of the spin-degenerate states with energy $\epsilon_m$ and on-site Coulomb repulsion $U$. The electrons are represented by the spinor $\psi_m = (\psi_{m\uparrow}\psi_{m\downarrow})^t$, the number operator $n_m = n_{m\uparrow} + n_{m\downarrow}$, where $n_{m\sigma} = \psi_{m\sigma}^\dagger \psi_{m\sigma}$, $\sigma =\uparrow, \downarrow$, and the spin operator $\boldsymbol{s}_m = \psi_m^\dagger \boldsymbol{\sigma} \psi_m / 2$, where $\boldsymbol{\sigma}$ is the vector of Pauli matrices.

Nearest neighbor hopping ($t$) is captured by the second term in $\mathcal{H}_{mol}$ while chirality ($\boldsymbol{v}_m^{(s)}$, $s = \pm$) and spin-orbit interaction ($\lambda$) is represented by the third term through the next-nearest neighbor hopping. The chirality vector $\boldsymbol{v}_m^{(s)} = \boldsymbol{d}_m \times \boldsymbol{d}_{m+1}$, where the unit vector $\boldsymbol{d}_m = (\boldsymbol{r}_m - \boldsymbol{r}_{m+1})/|\boldsymbol{r}_m - \boldsymbol{r}_{m+1}|$, defines the curvature of the path electrons traverses by hopping between sites $m$ and $m + 2s$ via the intermediate site $m + s$. By coupling the chirality to the Pauli matrices we account for the spin-orbit coupling directly (and indirectly) associated with chirality. In the last term of the model $\mathcal{H}_{mol}$, we also account for nearest neighbor Coulomb and spin interactions, on which the hypothesis for the weak temperature dependence is based.

The coordinate $\boldsymbol{r}_m$ of the (aperiodic) helical molecule is defined by

$$\boldsymbol{r}_m = (a cos\phi_m, a sin\phi_m, c\phi_m/2\pi),$$

where $a$ and $c$ denote the radius of the helix and distance in $z$-direction between two adjacent sites, whereas the angle $\phi_m = 2\pi(m-1)/(\mathbb{M}-1)$, for $m = 1,2, \ldots, \mathbb{M}$.

The charge current is calculated using standard methods and we use the expression

$$J_\sigma = \frac{ie}{h} \int \boldsymbol{\Gamma}^L(\sigma)(f_L(\omega) \boldsymbol{G}_1^>([\boldsymbol{\Gamma}^L(\sigma)]; \omega) + f_L(-\omega) \boldsymbol{G}_1^<([\boldsymbol{\Gamma}^L(\sigma)]; \omega)) d\omega \quad (1)$$

where $\boldsymbol{G}_1^{</>}([\boldsymbol{\Gamma}^L(\sigma)]; \omega)$ indicates that the electronic properties depend on the spin-polarization of the injected electrons from the left lead through the coupling parameter $\boldsymbol{\Gamma}^L(\sigma)$. The coupling parameter $\boldsymbol{\Gamma}^\chi = \Gamma^\chi(\sigma^0 + p_\chi \sigma^z)/2$ defines the effective spin-resolved tunneling rate between the lead $\chi$ and the adjacent molecular site, where $p_\chi \in$

$[-1,1]$ denotes the spin-polarization of the tunneling rate, whereas $\Gamma^{\chi} = \pi \sum_{\boldsymbol{k}\in\chi} |\boldsymbol{\chi}_{\boldsymbol{k}}|^2 \rho_{\chi}(\epsilon_{\boldsymbol{k}})$ accounts for the tunneling rate $\boldsymbol{\chi}_{\boldsymbol{k}} = \boldsymbol{L}_{\boldsymbol{k}}, \boldsymbol{R}_{\boldsymbol{k}}$, between the lead and the molecule and the density of electron states $\rho_{\chi}(\epsilon_{\boldsymbol{k}})$ in the corresponding lead. Moreover, the lesser/greater single electron Green function $\boldsymbol{G}_1^{</>}([\boldsymbol{\Gamma}^L(\sigma)];\omega)$ is used to describe the non-equilibrium densities of occupied (<) and unoccupied (>) electron states in the molecule.

The single electron Green function $\boldsymbol{G} = \{\boldsymbol{G}_{mn}\}_{mn}$ is a matrix comprising the $\mathbb{M}$ sites of the molecule, where each component $\boldsymbol{G}_{mn} = \langle\langle \psi_m | \psi_n^\dagger \rangle\rangle$ in itself is a $2 \times 2$-matrix in spin 1/2 space. Because of the electron-electron interactions, it is preferable to expand the spinors into many-body operators by setting $\psi_{m\sigma} = X_m^{0\sigma} + \eta_\sigma X_m^{\bar{\sigma}2}$, where $X_m^{pq} \equiv |mp\rangle\langle mq|$ described the transition between the states $q$ and $p$ at site $m$. This expansion of the Hilbert space at each site allows us to include the effects of electronic interactions without limitations for the interaction parameters $U$, $U^{'}$, and $J$, as well as correctly accounting for the non-equilibrium conditions. The pertinent Green function is determined by the equation of motion in the mean-field approximation (Hubbard-I-approximation) under the self-consistent condition that the on-site occupation numbers satisfy

$$N_{m0} + N_{m\sigma} = \frac{i}{2\pi} \int \left( \sum_{\sigma'} (\boldsymbol{G}_m^{>}(\omega))_{0\sigma'} - (\boldsymbol{G}_m^{<}(\omega))_{0\sigma} \right) d\omega, \quad \text{(2a)}$$

$$N_{m\sigma} + N_{m2} = \frac{i}{2\pi} \int \left( (\boldsymbol{G}_m^{>}(\omega))_{\sigma 2} - \sum_{\sigma'} (\boldsymbol{G}_m^{<}(\omega))_{\sigma' 2} \right) d\omega, \quad \text{(2b)}$$

where the subscripts on the parentheses indicate which transitions are involved in the determination of the occupation numbers $N_{mp}$, $p = 0, \uparrow, \downarrow, 2$, for the empty, singly occupied and doubly occupied states. For more details of the implementation of many-body operators in Green function as well as the calculations of the electronic structure and transport properties, we refer to Refs. 22-25.

## References


1. Safarowsky, O., Nieger, M., Fröhlich, R. & Vögtle, F. A Molecular Knot with Twelve Amide Groups—One-Step Synthesis, Crystal Structure, Chirality. *Angew. Chem. Int. Ed.* **39**, 1616-1618 (2000).
2. Vögtle, F. et al. Novel Amide-Based Molecular Knots: Complete Enantiomeric Separation, Chiroptical Properties, and Absolute Configuration. *Angew. Chem. Int. Ed.* **40**, 2468-2471 (2001).
3. Suda, M. et al. Light-driven molecular switch for reconfigurable spin filters. *Nat. Commun.* **10**, 2455 (2019).
4. Zhu, Q. et al. Multistate Switching of Spin Selectivity in Electron Transport through Light-Driven Molecular Motors. *Adv. Sci.* **8**, 2101773 (2021).
5. Mishra, S. et al. Length-Dependent Electron Spin Polarization in Oligopeptides and DNA. *J. Phys. Chem. C* **124**, 10776-10782 (2020).
6. Ko, C. H. et al. Twisted molecular wires polarize spin currents at room temperature. *Proc. Natl. Acad. Sci. U.S.A.* **119**, 2116180119 (2022).
7. Bullard, G. et al. Low-Resistance Molecular Wires Propagate Spin-Polarized Currents. *J Am Chem Soc* **141**, 14707-14711 (2019).
8. Kulkarni, C. et al. Highly Efficient and Tunable Filtering of Electrons' Spin by Supramolecular Chirality of Nanofiber-Based Materials. *Adv. Mater.* **32**, 1904965 (2020).
9. Mondal, A. K. et al. Spin Filtering in Supramolecular Polymers Assembled from Achiral Monomers Mediated by Chiral Solvents. *J. Am. Chem. Soc.* **143**, 7189-7195 (2021).
10. Huizi-Rayo, U. et al. An Ideal Spin Filter: Long-Range, High-Spin Selectivity in Chiral Helicoidal 3-Dimensional Metal Organic Frameworks. *Nano Lett* **20**, 8476-8482 (2020).
11. Mondal, A. K. et al. Long-Range Spin-Selective Transport in Chiral Metal-Organic Crystals with Temperature-Activated Magnetization. *ACS nano* **14**, 16624-16633 (2020).
12. Kiran, V., Mathew, S. P., Cohen, S. R., Hernandez Delgado, I., Lacour, J. & Naaman, R. Helicenes--A New Class of Organic Spin Filter. *Adv. Mater.* **28**, 1957-1962 (2016).
13. Rodriguez, R. et al. Mutual Monomer Orientation To Bias the Supramolecular Polymerization of [6]Helicenes and the Resulting Circularly Polarized Light and Spin Filtering Properties. *J. Am. Chem. Soc.* **144**, 7709-7719 (2022).
14. Bloom, B. P., Kiran, V., Varade, V., Naaman, R. & Waldeck, D. H. Spin Selective Charge Transport through Cysteine Capped CdSe Quantum Dots. *Nano Lett* **16**, 4583-4589 (2016).
15. Mishra, S. et al. Spin Filtering Along Chiral Polymers. *Angew.Chem. Int. Ed.* **59**, 14671-14676 (2020).
16. Tassinari, F., Banerjee-Ghosh, K., Parenti, F., Kiran, V., Mucci, A. & Naaman, R. Enhanced Hydrogen Production with Chiral Conductive Polymer-Based Electrodes. *The Journal of Physical Chemistry C* **121**, 15777-15783 (2017).
17. Bhowmick, D. K. et al. Spin-induced asymmetry reaction—The formation of asymmetric carbon by electropolymerization. *Sci. Adv.* **8**, eabq2727 (2022).

18. Lu, H. et al. Highly Distorted Chiral Two-Dimensional Tin Iodide Perovskites for Spin Polarized Charge Transport. *J. Am. Chem. Soc.* **142**, 13030-13040 (2020).
19. Lu, Y. et al. Spin-Dependent Charge Transport in 1D Chiral Hybrid Lead-Bromide Perovskite with High Stability. *Adv. Funct. Mater.* **31**, (2021).
20. Lu, H. P. et al. Spin-dependent charge transport through 2D chiral hybrid lead-iodide perovskites. *Sci. Adv.* **5**, eaay0571 (2019).
21. Kim, Y.-H. et al. Chiral-induced spin selectivity enables a room-temperature spin light-emitting diode. *Science* **371**, 1129-1133 (2021).
22. J. Fransson, Chirality-Induced Spin Selectivity: The Role of Electron Correlations, *J. Phys. Chem. Lett.* **2019**, *10*, 7126-7132.
23. J. Fransson, Non-equilibrium theory for a quantum dot with arbitrary on-site correlation strength coupled to leads, *Phys. Rev. B*, **2005**, *72*, 075314.
24. J. Fransson, M. Råsander, Pauli spin blockade in weakly coupling double quantum dots, *Phys. Rev. B*, **2006**, *73*, 205333.
25. J. Fransson, Non-Equilibrium Nano-Physics, Springer-Verlag, **2010**.
