# Supplementary material for: Highly Conductive Topologically Chiral Molecular Knots as Efficient Spin Filters
Source: J Am Chem Soc. 2023 Nov 16;145(49):26791–8. doi: 10.1021/jacs.3c08966 (PMC10722505; doi:10.1021/jacs.3c08966)

checkCIF/PLATON report

Structure factors have been supplied for datablock(s) exp_2788

THIS REPORT IS FOR GUIDANCE ONLY. IF USED AS PART OF A REVIEW PROCEDURE FOR PUBLICATION, IT SHOULD NOT REPLACE THE EXPERTISE OF AN EXPERIENCED CRYSTALLOGRAPHIC REFEREE.

No syntax errors found.   CIF dictionary   Interpreting this report

Datablock: exp_2788

Bond precision:	C-C = 0.0061 A		Wavelength=1.54184
Cell:	a=18.2626(3)	b=24.9409(4)	c=28.3395(3)
	alpha=78.206(1)	beta=86.902(1)	gamma=71.029(1)
Temperature:	236 K		

	Calculated	Reported
Volume	11948.3(3)	11948.3(3)
Space group	P -1	P -1
Hall group	-P 1	-P 1
Moiety formula	C177 H188 N15 O12, 8(C2 H6 O S) [+ solvent]	C177 H188 N15 O12, 8(C2 H6 O S), 2[C2H6SO]
Sum formula	C193 H236 N15 O20 S8 [+ solvent]	C197 H248 N15 O22 S10
Mr	3342.46	3498.69
Dx,g cm-3	0.929	0.972
Z	2	2
Mu (mm-1)	1.104	1.286
F000	3574.0	3742.0
F000'	3588.72	
h,k,lmax	21,29,33	21,29,33
Nref	42699	42378
Tmin,Tmax	0.641,0.716	0.555,1.000
Tmin'	0.577	

Correction method= # Reported T Limits: Tmin=0.555 Tmax=1.000
AbsCorr = MULTI-SCAN

Data completeness= 0.992   Theta(max)= 67.079

```
R(reflections)= 0.1061( 27169)            wR2(reflections)= 0.2727( 42378)
S = 0.832                  Npar= 2259
```

The following ALERTS were generated. Each ALERT has the format
test-name_ALERT_alert-type_alert-level.
Click on the hyperlinks for more details of the test.

Alert level B

```
PLAT910_ALERT_3_B Missing # of FCF Reflection(s) Below Theta(Min).         16 Note
```

Alert level C

```
PLAT082_ALERT_2_C High R1 Value ..................................       0.11 Report
PLAT084_ALERT_3_C High wR2 Value (i.e. > 0.25) ...................       0.27 Report
PLAT213_ALERT_2_C Atom O2              has ADP max/min Ratio .....        4.0 prolat
PLAT213_ALERT_2_C Atom N2              has ADP max/min Ratio .....        3.1 prolat
PLAT213_ALERT_2_C Atom C128            has ADP max/min Ratio .....        3.2 prolat
PLAT213_ALERT_2_C Atom C129            has ADP max/min Ratio .....        3.1 prolat
PLAT213_ALERT_2_C Atom C130            has ADP max/min Ratio .....        3.4 prolat
PLAT213_ALERT_2_C Atom C144            has ADP max/min Ratio .....        3.1 prolat
PLAT213_ALERT_2_C Atom C146            has ADP max/min Ratio .....        3.4 prolat
PLAT213_ALERT_2_C Atom C147            has ADP max/min Ratio .....        3.1 prolat
PLAT220_ALERT_2_C NonSolvent   Resd 1  C   Ueq(max)/Ueq(min) Range        4.8 Ratio
PLAT220_ALERT_2_C NonSolvent   Resd 1  N   Ueq(max)/Ueq(min) Range        3.1 Ratio
PLAT220_ALERT_2_C NonSolvent   Resd 1  O   Ueq(max)/Ueq(min) Range        4.0 Ratio
PLAT222_ALERT_3_C NonSolvent Resd 1  H   Uiso(max)/Uiso(min) Range        5.7 Ratio
PLAT230_ALERT_2_C Hirshfeld Test Diff for    C16      --C17      .        6.6 s.u.
PLAT230_ALERT_2_C Hirshfeld Test Diff for    C17      --C18      .        5.9 s.u.
PLAT230_ALERT_2_C Hirshfeld Test Diff for    C24      --C25      .        6.3 s.u.
PLAT230_ALERT_2_C Hirshfeld Test Diff for    C114     --C115     .        5.3 s.u.
PLAT230_ALERT_2_C Hirshfeld Test Diff for    C135     --C139     .        6.2 s.u.
PLAT234_ALERT_4_C Large Hirshfeld Difference C19      --C20      .       0.16 Ang.
PLAT234_ALERT_4_C Large Hirshfeld Difference C43      --C37A     .       0.17 Ang.
PLAT241_ALERT_2_C High   'MainMol' Ueq as Compared to Neighbors of        C18 Check
PLAT241_ALERT_2_C High   'MainMol' Ueq as Compared to Neighbors of       C130 Check
PLAT241_ALERT_2_C High   'MainMol' Ueq as Compared to Neighbors of       C173 Check
PLAT242_ALERT_2_C Low    'MainMol' Ueq as Compared to Neighbors of       C148 Check
PLAT242_ALERT_2_C Low    'MainMol' Ueq as Compared to Neighbors of       C153 Check
PLAT244_ALERT_4_C Low    'Solvent' Ueq as Compared to Neighbors of       S007 Check
PLAT244_ALERT_4_C Low    'Solvent' Ueq as Compared to Neighbors of        S50 Check
PLAT244_ALERT_4_C Low    'Solvent' Ueq as Compared to Neighbors of       S300 Check
PLAT244_ALERT_4_C Low    'Solvent' Ueq as Compared to Neighbors of       S500 Check
PLAT244_ALERT_4_C Low    'Solvent' Ueq as Compared to Neighbors of       S600 Check
PLAT244_ALERT_4_C Low    'Solvent' Ueq as Compared to Neighbors of       S700 Check
PLAT250_ALERT_2_C Large U3/U1 Ratio for Average U(i,j) Tensor ....        2.1 Note
PLAT260_ALERT_2_C Large Average Ueq of Residue Including      S007      0.171 Check
PLAT260_ALERT_2_C Large Average Ueq of Residue Including       S50      0.207 Check
PLAT260_ALERT_2_C Large Average Ueq of Residue Including      S200      0.180 Check
PLAT260_ALERT_2_C Large Average Ueq of Residue Including      S300      0.139 Check
PLAT260_ALERT_2_C Large Average Ueq of Residue Including      S500      0.155 Check
PLAT260_ALERT_2_C Large Average Ueq of Residue Including      S700      0.164 Check
PLAT334_ALERT_2_C Small <C-C> Benzene Dist.   C148     -C153     .       1.36 Ang.
PLAT340_ALERT_3_C Low Bond Precision on  C-C Bonds ..............    0.00607 Ang.
```

```
PLAT410_ALERT_2_C Short Intra H...H Contact  H13H     ..H138     .       1.99 Ang.
                                                      x,y,z  =      1_555 Check
PLAT420_ALERT_2_C D-H Bond Without Acceptor  N7       --H7       .     Please Check
PLAT420_ALERT_2_C D-H Bond Without Acceptor  N11      --H11      .     Please Check
PLAT420_ALERT_2_C D-H Bond Without Acceptor  N14      --H14      .     Please Check
PLAT906_ALERT_3_C Large K Value in the Analysis of Variance ......      3.217 Check
PLAT911_ALERT_3_C Missing FCF Refl Between Thmin & STh/L=    0.597        306 Report
PLAT913_ALERT_3_C Missing # of Very Strong Reflections in FCF ....          4 Note
PLAT918_ALERT_3_C Reflection(s) with I(obs) much Smaller I(calc) .          5 Check
PLAT975_ALERT_2_C Check Calcd Resid. Dens.  0.52Ang From O05I    .       0.49 eA-3
PLAT976_ALERT_2_C Check Calcd Resid. Dens.  0.98Ang From O50A    .      -0.41 eA-3
```

Alert level G

```
FORMU01_ALERT_2_G  There is a discrepancy between the atom counts in the
            _chemical_formula_sum and the formula from the _atom_site* data.
            Atom count from _chemical_formula_sum:C197 H248 N15 O22 S10
            Atom count from the _atom_site data:  C193.0049 H236.0049 N15 O20 S8
CELLZ01_ALERT_1_G Difference between formula and atom_site contents detected.
CELLZ01_ALERT_1_G ALERT: Large difference may be due to a
            symmetry error - see SYMMG tests
           From the CIF: _cell_formula_units_Z    2
           From the CIF: _chemical_formula_sum  C197 H248 N15 O22 S10
           TEST: Compare cell contents of formula and atom_site data

           atom    Z*formula  cif sites diff
           C        394.00    386.00    8.00
           H        496.00    472.00   24.00
           N         30.00     30.00    0.00
           O         44.00     40.00    4.00
           S         20.00     16.00    4.00
PLAT002_ALERT_2_G Number of Distance or Angle Restraints on AtSite         26 Note
PLAT003_ALERT_2_G Number of Uiso or Uij Restrained non-H Atoms ...         42 Report
PLAT007_ALERT_5_G Number of Unrefined Donor-H Atoms .............          12 Report
PLAT041_ALERT_1_G Calc. and Reported SumFormula    Strings  Differ     Please Check
PLAT051_ALERT_1_G Mu(calc) and Mu(CIF) Ratio Differs from 1.0 by .      14.17 %
PLAT072_ALERT_2_G SHELXL First  Parameter in WGHT  Unusually Large       0.13 Report
PLAT083_ALERT_2_G SHELXL Second Parameter in WGHT  Unusually Large      25.66 Why ?
PLAT154_ALERT_1_G The s.u.'s on the Cell Angles are Equal ..(Note)      0.001 Degree
PLAT172_ALERT_4_G The CIF-Embedded .res File Contains DFIX Records          8 Report
PLAT176_ALERT_4_G The CIF-Embedded .res File Contains SADI Records         25 Report
PLAT177_ALERT_4_G The CIF-Embedded .res File Contains DELU Records         15 Report
PLAT178_ALERT_4_G The CIF-Embedded .res File Contains SIMU Records         12 Report
PLAT188_ALERT_3_G A Non-default SIMU Restraint Value has been used     0.0100 Report
PLAT188_ALERT_3_G A Non-default SIMU Restraint Value has been used     0.0100 Report
PLAT188_ALERT_3_G A Non-default SIMU Restraint Value has been used     0.0100 Report
PLAT188_ALERT_3_G A Non-default SIMU Restraint Value has been used     0.0100 Report
PLAT188_ALERT_3_G A Non-default SIMU Restraint Value has been used     0.0100 Report
PLAT188_ALERT_3_G A Non-default SIMU Restraint Value has been used     0.0100 Report
PLAT188_ALERT_3_G A Non-default SIMU Restraint Value has been used     0.0100 Report
PLAT188_ALERT_3_G A Non-default SIMU Restraint Value has been used     0.0100 Report
PLAT188_ALERT_3_G A Non-default SIMU Restraint Value has been used     0.0100 Report
PLAT188_ALERT_3_G A Non-default SIMU Restraint Value has been used     0.0100 Report
PLAT188_ALERT_3_G A Non-default SIMU Restraint Value has been used     0.0100 Report
PLAT188_ALERT_3_G A Non-default SIMU Restraint Value has been used     0.0100 Report
PLAT191_ALERT_3_G A Non-default SADI Restraint Value has been used     0.0400 Report
PLAT191_ALERT_3_G A Non-default SADI Restraint Value has been used     0.0400 Report
```

```
PLAT191_ALERT_3_G A Non-default SADI Restraint Value has been used     0.0400 Report
PLAT191_ALERT_3_G A Non-default SADI Restraint Value has been used     0.0400 Report
PLAT191_ALERT_3_G A Non-default SADI Restraint Value has been used     0.0400 Report
PLAT191_ALERT_3_G A Non-default SADI Restraint Value has been used     0.0400 Report
PLAT191_ALERT_3_G A Non-default SADI Restraint Value has been used     0.0400 Report
PLAT191_ALERT_3_G A Non-default SADI Restraint Value has been used     0.0400 Report
PLAT191_ALERT_3_G A Non-default SADI Restraint Value has been used     0.0400 Report
PLAT191_ALERT_3_G A Non-default SADI Restraint Value has been used     0.0400 Report
PLAT191_ALERT_3_G A Non-default SADI Restraint Value has been used     0.0400 Report
PLAT191_ALERT_3_G A Non-default SADI Restraint Value has been used     0.0400 Report
PLAT191_ALERT_3_G A Non-default SADI Restraint Value has been used     0.0400 Report
PLAT192_ALERT_3_G A Non-default DELU Restraint Value for First Par     0.0010 Report
PLAT192_ALERT_3_G A Non-default DELU Restraint Value for SecondPar     0.0010 Report
PLAT192_ALERT_3_G A Non-default DELU Restraint Value for First Par     0.0010 Report
PLAT192_ALERT_3_G A Non-default DELU Restraint Value for SecondPar     0.0010 Report
PLAT192_ALERT_3_G A Non-default DELU Restraint Value for First Par     0.0010 Report
PLAT192_ALERT_3_G A Non-default DELU Restraint Value for SecondPar     0.0010 Report
PLAT192_ALERT_3_G A Non-default DELU Restraint Value for First Par     0.0010 Report
PLAT192_ALERT_3_G A Non-default DELU Restraint Value for SecondPar     0.0010 Report
PLAT192_ALERT_3_G A Non-default DELU Restraint Value for First Par     0.0010 Report
PLAT192_ALERT_3_G A Non-default DELU Restraint Value for SecondPar     0.0010 Report
PLAT192_ALERT_3_G A Non-default DELU Restraint Value for First Par     0.0010 Report
PLAT192_ALERT_3_G A Non-default DELU Restraint Value for SecondPar     0.0010 Report
PLAT192_ALERT_3_G A Non-default DELU Restraint Value for First Par     0.0010 Report
PLAT192_ALERT_3_G A Non-default DELU Restraint Value for SecondPar     0.0010 Report
PLAT192_ALERT_3_G A Non-default DELU Restraint Value for First Par     0.0010 Report
PLAT192_ALERT_3_G A Non-default DELU Restraint Value for SecondPar     0.0010 Report
PLAT192_ALERT_3_G A Non-default DELU Restraint Value for First Par     0.0010 Report
PLAT192_ALERT_3_G A Non-default DELU Restraint Value for SecondPar     0.0010 Report
PLAT192_ALERT_3_G A Non-default DELU Restraint Value for SecondPar     0.0200 Report
PLAT192_ALERT_3_G A Non-default DELU Restraint Value for SecondPar     0.0200 Report
PLAT192_ALERT_3_G A Non-default DELU Restraint Value for SecondPar     0.0200 Report
PLAT192_ALERT_3_G A Non-default DELU Restraint Value for SecondPar     0.0200 Report
PLAT192_ALERT_3_G A Non-default DELU Restraint Value for SecondPar     0.0200 Report
PLAT192_ALERT_3_G A Non-default DELU Restraint Value for SecondPar     0.0200 Report
PLAT301_ALERT_3_G Main Residue  Disorder .............(Resd  1  )         3% Note
PLAT302_ALERT_4_G Anion/Solvent/Minor-Residue Disorder (Resd  9  )       100% Note
PLAT302_ALERT_4_G Anion/Solvent/Minor-Residue Disorder (Resd 10  )       100% Note
PLAT304_ALERT_4_G Non-Integer Number of Atoms in ..... (Resd  9  )       6.76 Check
PLAT304_ALERT_4_G Non-Integer Number of Atoms in ..... (Resd 10  )       3.24 Check
PLAT343_ALERT_2_G Unusual sp?      Angle Range in Main Residue for       C145 Check
PLAT380_ALERT_4_G Incorrectly? Oriented X(sp2)-Methyl Moiety .....        C14 Check
PLAT410_ALERT_2_G Short Intra H...H Contact  H38A     ..H48      .       2.09 Ang.
                                                       x,y,z  =     1_555 Check
PLAT410_ALERT_2_G Short Intra H...H Contact  H42B     ..H44      .       2.08 Ang.
                                                       x,y,z  =     1_555 Check
PLAT410_ALERT_2_G Short Intra H...H Contact  H115     ..H38C     .       1.98 Ang.
                                                       x,y,z  =     1_555 Check
PLAT410_ALERT_2_G Short Intra H...H Contact  H115     ..H38D     .       2.04 Ang.
                                                       x,y,z  =     1_555 Check
PLAT413_ALERT_2_G Short Inter XH3 .. XHn     H15B     ..H41D     .       1.95 Ang.
                                                     1+x,y,z  =     1_655 Check
PLAT432_ALERT_2_G Short Inter X...Y Contact  O13      ..C501     .       2.68 Ang.
                                                       x,y,z  =     1_555 Check
PLAT432_ALERT_2_G Short Inter X...Y Contact  C154     ..C40A     .       3.07 Ang.
                                                     1+x,y,z  =     1_655 Check
PLAT606_ALERT_4_G Solvent Accessible VOID(S) in Structure .......          ! Info
```

```
PLAT720_ALERT_4_G Number of Unusual/Non-Standard Labels ..........         27 Note
PLAT790_ALERT_4_G Centre of Gravity not Within Unit Cell: Resd.  #          6 Note
              C2 H6 O S
PLAT860_ALERT_3_G Number of Least-Squares Restraints ............         376 Note
PLAT868_ALERT_4_G ALERTS Due to the Use of _smtbx_masks Suppressed          ! Info
PLAT909_ALERT_3_G Percentage of I>2sig(I) Data at Theta(Max) Still        36% Note
PLAT933_ALERT_2_G Number of HKL-OMIT Records in Embedded .res File        117 Note
PLAT941_ALERT_3_G Average HKL Measurement Multiplicity ...........        3.1 Low
PLAT961_ALERT_5_G Dataset Contains no Negative Intensities .......     Please Check
PLAT978_ALERT_2_G Number C-C Bonds with Positive Residual Density.          1 Info
```

```
   0 ALERT level A = Most likely a serious problem - resolve or explain
   1 ALERT level B = A potentially serious problem, consider carefully
  51 ALERT level C = Check. Ensure it is not caused by an omission or oversight
  88 ALERT level G = General information/check it is not something unexpected

   5 ALERT type 1 CIF construction/syntax error, inconsistent or missing data
  51 ALERT type 2 Indicator that the structure model may be wrong or deficient
  61 ALERT type 3 Indicator that the structure quality may be low
  21 ALERT type 4 Improvement, methodology, query or suggestion
   2 ALERT type 5 Informative message, check
```

It is advisable to attempt to resolve as many as possible of the alerts in all categories. Often the minor alerts point to easily fixed oversights, errors and omissions in your CIF or refinement strategy, so attention to these fine details can be worthwhile. In order to resolve some of the more serious problems it may be necessary to carry out additional measurements or structure refinements. However, the purpose of your study may justify the reported deviations and the more serious of these should normally be commented upon in the discussion or experimental section of a paper or in the "special_details" fields of the CIF. checkCIF was carefully designed to identify outliers and unusual parameters, but every test has its limitations and alerts that are not important in a particular case may appear. Conversely, the absence of alerts does not guarantee there are no aspects of the results needing attention. It is up to the individual to critically assess their own results and, if necessary, seek expert advice.

Publication of your CIF in IUCr journals

A basic structural check has been run on your CIF. These basic checks will be run on all CIFs submitted for publication in IUCr journals (*Acta Crystallographica*, *Journal of Applied Crystallography*, *Journal of Synchrotron Radiation*); however, if you intend to submit to *Acta Crystallographica Section C* or *E* or *IUCrData*, you should make sure that full publication checks are run on the final version of your CIF prior to submission.

Publication of your CIF in other journals

Please refer to the *Notes for Authors* of the relevant journal for any special instructions relating to CIF submission.

PLATON version of 06/07/2023; check.def file version of 30/06/2023

Datablock exp_2788 - ellipsoid plot

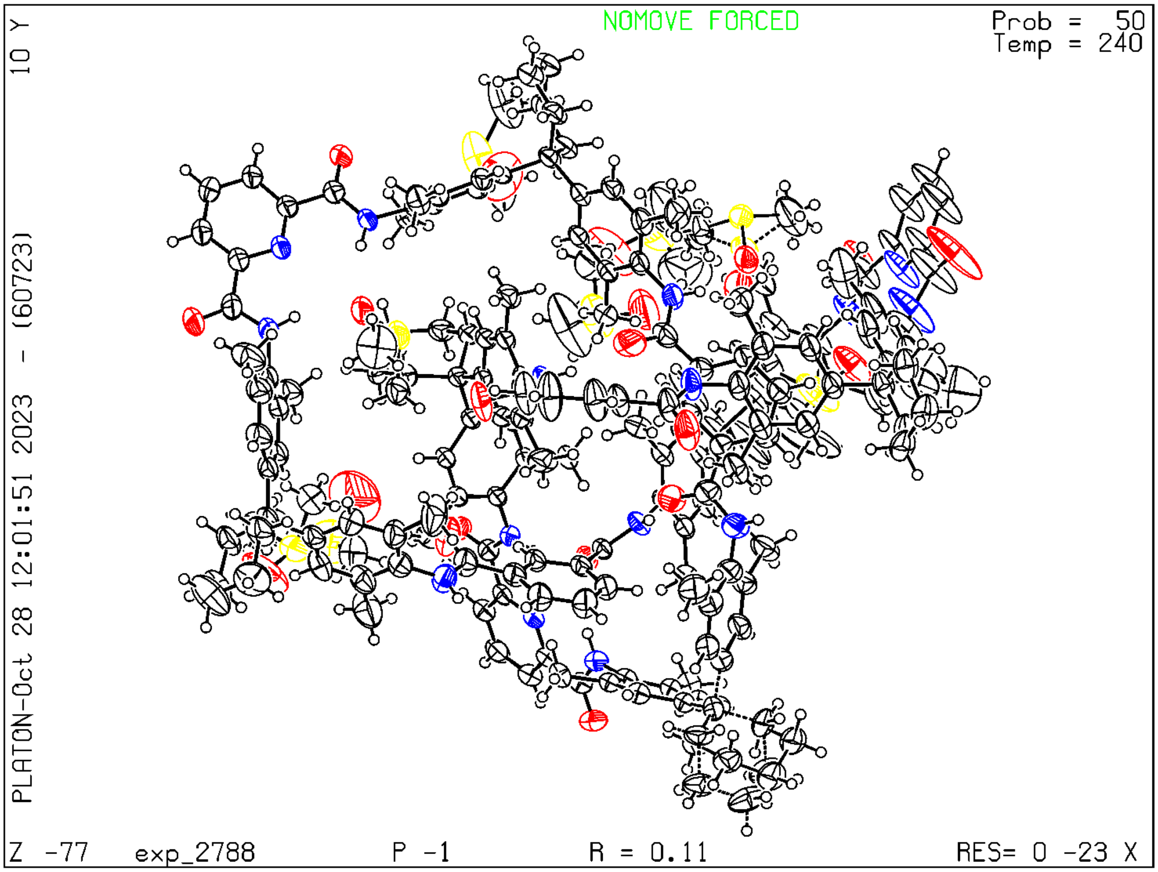

Supplement: Supplementary file 2 — ja3c08966_si_002.zip [file ja3c08966_si_002.zip › CCDC-2234794/exp_2788_cifreport.pdf]
