# Supplementary material for: Highly Conductive Topologically Chiral Molecular Knots as Efficient Spin Filters
Source: J Am Chem Soc. 2023 Nov 16;145(49):26791–8. doi: 10.1021/jacs.3c08966 (PMC10722505; doi:10.1021/jacs.3c08966)
Supplement: Supplementary file 2 — ja3c08966_si_002.zip [file ja3c08966_si_002.zip › CCDC-2234794/exp_2788_tables.html]

exp\_2788


# exp\_2788

Table 1 Crystal data and structure refinement for exp\_2788.

| Identification code | exp\_2788 |
| Empirical formula | C197H248N15O22S10 |
| Formula weight | 3498.69 |
| Temperature/K | 236(90) |
| Crystal system | triclinic |
| Space group | P-1 |
| a/Å | 18.2626(3) |
| b/Å | 24.9409(4) |
| c/Å | 28.3395(3) |
| α/° | 78.2060(10) |
| β/° | 86.9020(10) |
| γ/° | 71.0290(10) |
| Volume/Å3 | 11948.3(3) |
| Z | 2 |
| ρcalcg/cm3 | 0.972 |
| μ/mm‑1 | 1.286 |
| F(000) | 3742.0 |
| Crystal size/mm3 | 0.39 × 0.32 × 0.26 |
| Radiation | CuKα (λ = 1.54184) |
| 2Θ range for data collection/° | 7.716 to 134.158 |
| Index ranges | -21 ≤ h ≤ 21, -29 ≤ k ≤ 29, -33 ≤ l ≤ 23 |
| Reflections collected | 131463 |
| Independent reflections | 42378 [Rint = 0.1143, Rsigma = 0.0920] |
| Data/restraints/parameters | 42378/376/2259 |
| Goodness-of-fit on F2 | 0.832 |
| Final R indexes [I>=2σ (I)] | R1 = 0.1061, wR2 = 0.2567 |
| Final R indexes [all data] | R1 = 0.1269, wR2 = 0.2727 |
| Largest diff. peak/hole / e Å-3 | 0.93/-0.87 |

Table 2 Fractional Atomic Coordinates (×104) and Equivalent Isotropic Displacement Parameters (Å2×103) for exp\_2788. Ueq is defined as 1/3 of the trace of the orthogonalised UIJ tensor.

| Atom | *x* | *y* | *z* | U(eq) |
| --- | --- | --- | --- | --- |
| O1 | 10204.1(16) | -1640.7(15) | 5936.6(11) | 83.9(10) |
| O2 | 11305(2) | -2271(3) | 8370.6(14) | 201(4) |
| O3 | 1002.6(16) | 576.9(13) | 6143.0(12) | 79.7(9) |
| O4 | 2654.0(15) | 2642.1(11) | 4935.9(9) | 61.6(7) |
| O5 | 3696.4(17) | 7144.5(12) | 6215.6(13) | 86.8(10) |
| O6 | 7359.2(15) | 5494.6(11) | 5549.7(11) | 70.6(8) |
| O7 | 5096(2) | 3579.3(13) | 8244.0(11) | 89.6(10) |
| O8 | 6575(2) | 1235.1(14) | 10150.0(11) | 103.9(13) |
| O9 | 7211(2) | 1743.9(15) | 8145.5(18) | 119.0(16) |
| O10 | 5201.4(16) | 677.6(13) | 8009.8(9) | 71.4(8) |
| O11 | 3448.7(13) | 1263.2(10) | 6577.5(8) | 50.2(6) |
| O12 | 2653(2) | 3303.5(13) | 7018.4(12) | 98.6(12) |
| N1 | 10504(2) | -1920(2) | 7197.0(13) | 99.2(16) |
| N2 | 10059(2) | -1933(3) | 8121.1(14) | 144(3) |
| N3 | 9363.8(18) | -1512.8(19) | 6554.1(12) | 82.3(12) |
| N4 | 2007.9(15) | 1563.1(12) | 5672.4(11) | 50.0(7) |
| N5 | 1961.8(17) | 798.2(14) | 6463.7(13) | 63.9(9) |
| N6 | 3052.2(15) | 2128.3(11) | 5681.8(10) | 46.4(6) |
| N7 | 2062(2) | 3408.7(14) | 7723.6(13) | 69.9(9) |
| N8 | 4222.5(18) | 6192.6(13) | 6527.5(12) | 60.0(8) |
| N9 | 5525.8(16) | 6149.8(12) | 6025.8(10) | 51.0(7) |
| N10 | 6679.5(16) | 5102.2(12) | 6133.4(11) | 53.8(7) |
| N11 | 7234(2) | 1139.1(14) | 9464.1(12) | 79.1(12) |
| N12 | 5925.8(17) | 2772.0(13) | 8049.1(10) | 55.1(7) |
| N13 | 8232.9(18) | 1215.3(13) | 7781.3(11) | 58.0(8) |
| N14 | 5380.2(19) | -94.6(15) | 8628.5(11) | 66.4(9) |
| N15 | 4459.3(15) | 808.7(12) | 7104.8(10) | 49.3(7) |
| C1 | 6171.5(19) | 6119.4(15) | 5770.4(12) | 50.3(8) |
| C2 | 6301(2) | 6603.5(15) | 5480.5(14) | 56.0(9) |
| C3 | 5745(2) | 7131.7(16) | 5461.5(14) | 62.6(10) |
| C4 | 5069(2) | 7171.7(16) | 5716.4(14) | 62.1(10) |
| C5 | 4983(2) | 6670.2(15) | 5994.1(13) | 53.1(8) |
| C6 | 4244(2) | 6689.4(15) | 6253.4(15) | 61.2(10) |
| C7 | 3539(2) | 6117.8(14) | 6758.2(13) | 53.5(9) |
| C8 | 3134(2) | 5833.9(14) | 6549.5(12) | 52.4(9) |
| C9 | 2465(2) | 5768.4(15) | 6774.9(12) | 51.9(8) |
| C10 | 2196(2) | 5960.5(15) | 7195.5(13) | 53.7(9) |
| C11 | 2625(2) | 6229.3(18) | 7401.9(15) | 65.4(10) |
| C12 | 3299(2) | 6309.8(18) | 7185.1(16) | 64.4(10) |
| C13 | 3739(3) | 6613(2) | 7414(2) | 91.4(15) |
| C14 | 3417(2) | 5611.2(18) | 6098.3(14) | 63.0(10) |
| C15 | 1444(2) | 5872.5(15) | 7417.7(13) | 55.1(9) |
| C16 | 767(2) | 6237.8(19) | 7067.7(16) | 71.9(12) |
| C17 | -21(3) | 6304(3) | 7269(3) | 111(2) |
| C18 | -145(4) | 6491(4) | 7743(3) | 135(3) |
| C19 | 485(4) | 6075(3) | 8097(2) | 110(2) |
| C20 | 1254(3) | 6058(2) | 7908.7(16) | 73.8(12) |
| C21 | 1571(2) | 5217.4(16) | 7493.2(13) | 54.2(9) |
| C22 | 1174(2) | 4984.4(18) | 7242.2(16) | 69.5(11) |
| C23 | 1320(3) | 4389.5(19) | 7307.7(17) | 71.9(11) |
| C24 | 1897(2) | 4018.7(16) | 7637.7(14) | 59.0(9) |
| C25 | 2331(3) | 4244.2(18) | 7887.6(16) | 70.0(11) |
| C26 | 2148(3) | 4844.0(18) | 7811.0(15) | 66.2(10) |
| C27 | 2978(4) | 3862(2) | 8222(2) | 116(2) |
| C28 | 879(4) | 4162(2) | 7013(3) | 108(2) |
| C29 | 2452(2) | 3090.8(17) | 7409.0(15) | 66.7(11) |
| C30 | 2691(2) | 2437.1(16) | 7566.1(14) | 59.0(10) |
| C31 | 2613(2) | 2154.4(17) | 8031.7(14) | 67.4(11) |
| C32 | 2902(3) | 1564.5(18) | 8148.9(15) | 72.4(12) |
| C33 | 3290(2) | 1244.5(17) | 7817.0(13) | 60.8(10) |
| C34 | 3045.3(19) | 2114.3(14) | 7223.4(13) | 50.3(8) |
| C35 | 3357.4(18) | 1517.1(15) | 7354.0(12) | 49.5(8) |
| C36 | 3750.6(18) | 1190.0(14) | 6973.0(13) | 47.2(8) |
| C37 | 2470(5) | -460(4) | 8414(3) | 66(2) |
| C38 | 1757(5) | -198(4) | 8703(3) | 64(2) |
| C39 | 1831(4) | -540(3) | 9212(2) | 71.8(17) |
| C40 | 1960(4) | -1170(3) | 9237(2) | 73.3(18) |
| C41 | 2649(4) | -1428(3) | 8943(2) | 67.9(17) |
| C42 | 2547(5) | -1101(3) | 8433(3) | 61.0(19) |
| C43 | 2210(2) | -130.8(16) | 7895.4(17) | 66.0(9) |
| C44 | 2319(2) | -411.3(17) | 7516.7(18) | 69.8(10) |
| C45 | 2222(2) | -131.2(17) | 7037.2(17) | 67.9(12) |
| C46 | 1996(2) | 469.7(16) | 6938.1(16) | 62.2(10) |
| C47 | 1830(2) | 782.2(16) | 7317.6(17) | 63.6(11) |
| C48 | 1953(2) | 482.8(17) | 7785.7(17) | 66.6(11) |
| C49 | 1517(2) | 1436.3(17) | 7208.5(18) | 72.6(12) |
| C50 | 2387(3) | -466.3(19) | 6633.1(19) | 79.7(13) |
| C51 | 1465(2) | 849.1(17) | 6114.0(17) | 65.1(11) |
| C52 | 1521.2(19) | 1265.1(15) | 5660.2(15) | 55.0(9) |
| C53 | 1072(2) | 1331.2(17) | 5255.4(16) | 62.7(10) |
| C54 | 1124(2) | 1733.4(17) | 4850.9(15) | 59.1(9) |
| C55 | 1625.4(19) | 2036.9(15) | 4856.5(14) | 53.1(8) |
| C56 | 2058.6(18) | 1947.5(14) | 5274.2(13) | 47.2(8) |
| C57 | 2613.3(19) | 2272.3(14) | 5286.4(13) | 49.9(8) |
| C58 | 3545.6(17) | 2443.1(13) | 5768.4(11) | 43.0(7) |
| C59 | 3226.3(18) | 2996.5(14) | 5858.3(12) | 45.1(7) |
| C60 | 3713.9(17) | 3294.9(14) | 5940.6(12) | 45.5(7) |
| C61 | 4517.3(17) | 3067.1(13) | 5927.3(11) | 42.7(7) |
| C62 | 4818.1(18) | 2500.5(14) | 5847.7(12) | 45.3(7) |
| C63 | 4358.7(18) | 2183.8(13) | 5763.0(11) | 43.5(7) |
| C64 | 4716(2) | 1586.9(14) | 5654.8(14) | 55.3(9) |
| C65 | 2358.3(19) | 3270.9(16) | 5877.3(15) | 58.7(9) |
| C66 | 5052.8(18) | 3391.5(14) | 6031.9(12) | 44.5(7) |
| C67 | 5759.9(19) | 3288.6(15) | 5698.7(12) | 50.7(8) |
| C68 | 5531(2) | 3528.9(17) | 5164.5(13) | 58.3(9) |
| C69 | 5085(2) | 4178.4(17) | 5077.3(13) | 61.9(10) |
| C70 | 4397(2) | 4319.6(15) | 5410.1(13) | 55.1(9) |
| C71 | 4639(2) | 4050.7(14) | 5936.1(12) | 48.8(8) |
| C72 | 5306.8(18) | 3195.1(13) | 6568.6(12) | 45.2(7) |
| C73 | 5933.3(18) | 3319.3(14) | 6734.1(12) | 46.4(8) |
| C74 | 6141.8(18) | 3181.7(14) | 7220.2(12) | 48.6(8) |
| C8AA | 5721.4(19) | 2919.6(14) | 7548.7(12) | 51.0(8) |
| C76 | 5099(2) | 2771.5(16) | 7398.0(13) | 53.8(9) |
| C77 | 4907.2(19) | 2920.5(15) | 6913.7(13) | 52.4(8) |
| C78 | 4668(3) | 2455(2) | 7754.8(15) | 76.5(13) |
| C79 | 6828(2) | 3306.9(17) | 7386.7(13) | 55.8(9) |
| C80 | 6785(2) | 5546.7(16) | 5804.5(14) | 56.1(9) |
| C81 | 7267.2(18) | 4541.9(14) | 6217.6(13) | 48.8(8) |
| C82 | 7694(2) | 4362.7(15) | 6653.9(13) | 51.7(8) |
| C83 | 8303.4(19) | 3847.5(15) | 6714.6(12) | 48.7(8) |
| C84 | 8503.8(17) | 3508.5(14) | 6367.9(11) | 43.6(7) |
| C85 | 8049.7(18) | 3689.3(15) | 5951.1(12) | 46.8(8) |
| C86 | 7431.8(18) | 4209.3(14) | 5874.6(12) | 47.5(8) |
| C87 | 6969(2) | 4390.3(17) | 5412.3(14) | 61.7(10) |
| C88 | 7496(3) | 4717.8(18) | 7040.7(15) | 68.9(11) |
| C89 | 9178.4(18) | 2937.5(15) | 6467.7(12) | 47.8(8) |
| C90 | 9906.2(19) | 3048.1(17) | 6627.4(14) | 55.9(9) |
| C91 | 10242(2) | 3382.1(19) | 6223.8(16) | 67.7(11) |
| C92 | 10484(2) | 3083(2) | 5805.2(16) | 68.7(11) |
| C93 | 9812(2) | 2949.7(18) | 5623.3(15) | 62.4(10) |
| C94 | 9418.5(19) | 2646.6(16) | 6023.4(13) | 53.4(8) |
| C95 | 8910.0(18) | 2515.7(14) | 6848.6(11) | 44.5(7) |
| C96 | 9315(2) | 2214.7(16) | 7275.2(13) | 53.7(8) |
| C97 | 9094(2) | 1800.7(16) | 7592.9(13) | 57.7(9) |
| C98 | 8432(2) | 1682.2(14) | 7482.3(13) | 51.4(8) |
| C99 | 8002.4(18) | 1976.4(14) | 7059.2(13) | 49.7(8) |
| C100 | 8253.1(18) | 2385.6(14) | 6753.3(12) | 47.1(8) |
| C101 | 9567(3) | 1470(2) | 8044.8(15) | 81.2(13) |
| C102 | 7315(2) | 1834.1(17) | 6929.1(15) | 62.3(10) |
| C103 | 7618(2) | 1272.4(18) | 8064.1(15) | 63.8(10) |
| C104 | 7421(2) | 736.1(16) | 8252.4(13) | 56.5(9) |
| C105 | 7991(2) | 207.0(17) | 8419.1(13) | 62.3(10) |
| C106 | 7782(2) | -286.0(19) | 8572.7(14) | 68.1(11) |
| C107 | 7013(2) | -255.2(18) | 8556.3(13) | 64.6(10) |
| C108 | 6446(2) | 262.4(17) | 8384.1(12) | 55.1(9) |
| C109 | 6655(2) | 756.1(17) | 8233.3(13) | 59.4(10) |
| C110 | 5623(2) | 298.6(18) | 8328.4(13) | 61.4(10) |
| C111 | 4642(2) | -164.7(18) | 8576.5(13) | 61.4(10) |
| C112 | 4559(2) | -477.6(18) | 8251.5(14) | 60.6(10) |
| C113 | 3853(2) | -559.9(17) | 8215.0(14) | 61.0(10) |
| C114 | 3228(2) | -343.1(17) | 8501.1(15) | 61.7(10) |
| C115 | 3338(2) | -31.2(18) | 8827.1(16) | 71.3(12) |
| C116 | 4032(3) | 67.4(18) | 8870.4(14) | 66.2(11) |
| C117 | 4131(3) | 425(2) | 9212.7(18) | 87.8(14) |
| C118 | 5219(2) | -736(2) | 7936.6(16) | 72.5(12) |
| C119 | 4961.5(18) | 477.8(14) | 6786.3(11) | 46.5(8) |
| C120 | 5646.7(18) | 584.4(15) | 6643.7(12) | 48.6(8) |
| C121 | 6100.0(19) | 287.6(16) | 6307.0(12) | 53.6(9) |
| C122 | 5877.1(19) | -93.0(17) | 6104.0(13) | 57.2(10) |
| C123 | 5208(2) | -211.9(16) | 6275.4(14) | 59.1(9) |
| C124 | 4740.1(19) | 67.5(15) | 6613.1(13) | 51.8(8) |
| C125 | 4022(2) | -71.4(17) | 6786.2(16) | 64.3(10) |
| C126 | 5885(2) | 1032.4(19) | 6827.2(15) | 62.2(10) |
| C127 | 6345(2) | -344(2) | 5686.1(15) | 79.8(10) |
| C128 | 6295(2) | 155(3) | 5269.1(15) | 95.5(11) |
| C129 | 6667(3) | -47(3) | 4804.2(18) | 128.1(16) |
| C130 | 6351(3) | -489(4) | 4663(2) | 148(2) |
| C131 | 6408(3) | -962(3) | 5066(2) | 132.5(16) |
| C132 | 5982(2) | -769(3) | 5518(2) | 103.7(12) |
| C133 | 7175(2) | -687(2) | 5873.4(14) | 65.5(11) |
| C134 | 7733(2) | -415(2) | 5820.2(14) | 68.8(12) |
| C135 | 8476(2) | -697(2) | 6024.9(14) | 67.9(12) |
| C136 | 8650(2) | -1241(2) | 6284.7(13) | 68.9(12) |
| C137 | 8120(2) | -1544(2) | 6330.3(14) | 75.8(13) |
| C138 | 7384(2) | -1249(2) | 6123.2(15) | 74.8(12) |
| C139 | 9046(3) | -361(3) | 5965(2) | 100.7(18) |
| C140 | 8316(3) | -2156(3) | 6609(2) | 103.2(18) |
| C141 | 10065(2) | -1658(2) | 6365.3(15) | 75.1(13) |
| C143 | 11473(3) | -1944(3) | 6593.2(18) | 100.3(19) |
| C144 | 12044(3) | -2134(3) | 6947(2) | 130(3) |
| C145 | 11853(3) | -2194(4) | 7421(2) | 130(3) |
| C146 | 11063(3) | -2085(3) | 7531.3(18) | 119(3) |
| C147 | 10826(3) | -2130(4) | 8045(2) | 150(4) |
| C148 | 9749(3) | -1797(3) | 8569.6(17) | 108.2(13) |
| C149 | 9399(4) | -2084(3) | 8863(2) | 125.7(15) |
| C150 | 9125(4) | -1952(3) | 9315(2) | 104.7(14) |
| C151 | 9171(2) | -1492(2) | 9447.8(13) | 69.9(9) |
| C152 | 9508(3) | -1150(2) | 9107.7(15) | 91.2(12) |
| C153 | 9763(3) | -1304(3) | 8673.4(16) | 106.3(13) |
| C154 | 10069(5) | -870(3) | 8300(2) | 178(3) |
| C156 | 8889(2) | -1307.4(16) | 9934.3(12) | 56.9(9) |
| C157 | 9625(2) | -1453.9(17) | 10250.0(13) | 62.8(10) |
| C158 | 9437(3) | -1338.6(19) | 10756.6(14) | 73.2(12) |
| C159 | 8919(3) | -1665(2) | 11013.6(14) | 78.5(13) |
| C160 | 8195(3) | -1545.8(18) | 10716.7(13) | 68.6(11) |
| C161 | 8409(3) | -1669.9(17) | 10202.4(13) | 65.2(10) |
| C162 | 8439(2) | -659.5(16) | 9841.5(12) | 59.1(10) |
| C163 | 7632(2) | -450.9(17) | 9782.9(12) | 59.8(10) |
| C164 | 7226(2) | 143.5(17) | 9664.4(12) | 64.2(11) |
| C165 | 7628(2) | 532.4(17) | 9597.1(14) | 69.6(12) |
| C166 | 8430(3) | 344.9(18) | 9647.8(16) | 78.2(14) |
| C167 | 8815(2) | -254.7(17) | 9780.0(15) | 70.4(12) |
| C168 | 8893(3) | 757(2) | 9567(2) | 102.5(19) |
| C169 | 6351(2) | 327(2) | 9611.9(15) | 73.8(12) |
| C170 | 5592(2) | 3104.0(16) | 8363.7(13) | 60.8(10) |
| C171 | 5833(2) | 2876.4(17) | 8880.4(14) | 67.6(11) |
| C172 | 5690(3) | 3253(2) | 9195.2(17) | 96.4(18) |
| C173 | 5888(4) | 3050(2) | 9675.8(18) | 117(2) |
| C174 | 6238(3) | 2468(2) | 9849.4(16) | 95.4(18) |
| C175 | 6389(3) | 2084.8(18) | 9544.4(14) | 71.9(12) |
| C176 | 6201(2) | 2284.8(16) | 9061.3(13) | 62.0(10) |
| C177 | 6760(3) | 1450.7(19) | 9751.2(14) | 80.8(15) |
| S600 | 5310.6(6) | 4629.3(4) | 7244.7(4) | 67.9(3) |
| S200 | 1281(2) | 3984.5(16) | 3714.9(11) | 184.1(10) |
| S800 | 9951.7(8) | 380.6(6) | 7163.5(5) | 59.0(5) |
| S500 | 7979.7(8) | -1042.4(9) | 7660.0(4) | 122.5(7) |
| S007 | 8383.9(14) | 3361.3(9) | 4422.5(9) | 153.1(9) |
| S300 | 1033.6(8) | 4912.0(7) | 5448.7(8) | 124.4(7) |
| S700 | 7099.5(13) | 1612.3(9) | 5641.2(7) | 136.5(7) |
| C200 | 1018(4) | 4739(4) | 3636(3) | 179(4) |
| C301 | 1007(3) | 5327(3) | 4874(3) | 115(2) |
| C701 | 6673(7) | 1809(5) | 5081(4) | 232(6) |
| C1AA | 7858(4) | 1792(3) | 5531.3(18) | 110(2) |
| C501 | 7907(5) | -355(4) | 7304(2) | 178(5) |
| C801 | 9373(7) | 750(5) | 6635(3) | 77(3) |
| C600 | 4453(2) | 4461.4(19) | 7213.8(16) | 73.4(12) |
| C800 | 10357(6) | -291(4) | 6967(3) | 59.1(15) |
| O800 | 9386(3) | 281.9(18) | 7547.0(17) | 71.0(13) |
| C201 | 1345(4) | 3827(3) | 4346(3) | 122(2) |
| O05I | 8320(4) | 3129(3) | 4875(2) | 197(3) |
| C500 | 7185(4) | -1135(6) | 7394(4) | 212(6) |
| C601 | 4992(5) | 5013(3) | 7735(2) | 135(3) |
| O600 | 5334.6(17) | 5070.0(12) | 6801.8(12) | 84.6(10) |
| O200 | 2125(5) | 3771(4) | 3548(3) | 237(4) |
| C0AA | 8137(6) | 2909(3) | 4126(3) | 161(3) |
| O300 | 457(3) | 5303(3) | 5724(2) | 205(3) |
| O700 | 7534(4) | 1011(2) | 5702(3) | 178(3) |
| O500 | 8676.5(19) | -1462.3(19) | 7499.0(11) | 106.2(13) |
| C071 | 9445(5) | 3139(4) | 4327(3) | 174(3) |
| C300 | 1935(3) | 4882(3) | 5632(3) | 112(2) |
| C155 | 9256(7) | -2587(5) | 8709(4) | 204(4) |
| C5AA | 10715(2) | -1864(2) | 6738.0(16) | 86.8(16) |
| S50 | 8765.3(15) | 801.6(14) | 4438.1(11) | 175.3(10) |
| O50A | 8065(4) | 1293(4) | 4385(3) | 216(3) |
| C2AA | 9239(7) | 825(5) | 4917(4) | 206(4) |
| C4AA | 8429(8) | 327(7) | 4694(5) | 234(4) |
| C37A | 2415(7) | -392(6) | 8453(4) | 85(7) |
| C38A | 1929(6) | -165(6) | 8881(4) | 60(4) |
| C39A | 1187(6) | -321(5) | 8889(4) | 73(4) |
| C40A | 1379(8) | -977(5) | 8986(4) | 87(4) |
| C41A | 1933(7) | -1244(6) | 8620(4) | 80(4) |
| C42A | 2638(7) | -1062(5) | 8621(4) | 62(4) |
| S8 | 9457.8(19) | 189.9(15) | 6991.6(12) | 68.8(13) |
| C75 | 9278(16) | 758(11) | 6494(6) | 86(6) |
| C1A | 10378(16) | -295(13) | 7155(6) | 122(9) |
| O13 | 8872(8) | 247(5) | 7348(4) | 99(4) |

Table 3 Anisotropic Displacement Parameters (Å2×103) for exp\_2788. The Anisotropic displacement factor exponent takes the form: -2π2[h2a\*2U11+2hka\*b\*U12+…].

| Atom | U11 | U22 | U33 | U23 | U13 | U12 |
| --- | --- | --- | --- | --- | --- | --- |
| O1 | 44.4(15) | 111(2) | 64.5(18) | -21.8(16) | -8.2(13) | 21.9(15) |
| O2 | 64(2) | 377(9) | 80(2) | -97(4) | -38(2) | 78(4) |
| O3 | 49.1(16) | 76.6(19) | 109(2) | 9.5(17) | 1.6(15) | -31.2(15) |
| O4 | 59.1(15) | 61.3(15) | 59.9(15) | 9.3(12) | -4.2(12) | -25.4(13) |
| O5 | 56.1(17) | 49.9(16) | 124(3) | 0.9(16) | 26.4(17) | 8.6(13) |
| O6 | 44.4(14) | 53.1(15) | 93(2) | 8.8(13) | 20.6(13) | -4.5(11) |
| O7 | 92(2) | 62.2(17) | 66.8(18) | 4.0(14) | -5.8(16) | 30.3(16) |
| O8 | 118(3) | 77(2) | 60.8(18) | 17.3(15) | 31.1(18) | 21.2(19) |
| O9 | 84(2) | 74(2) | 204(4) | -60(3) | 85(3) | -24.4(19) |
| O10 | 62.5(17) | 84.9(19) | 52.8(15) | 13.7(14) | -5.8(12) | -20.5(15) |
| O11 | 37.3(12) | 48.4(13) | 51.1(13) | -1.5(10) | 1.7(10) | -0.3(10) |
| O12 | 141(3) | 51.8(16) | 73(2) | -3.0(14) | 45(2) | -3.8(18) |
| N1 | 41.6(19) | 151(4) | 62(2) | -34(2) | -13.2(16) | 37(2) |
| N2 | 57(2) | 256(7) | 60(2) | -62(3) | -20.3(19) | 52(3) |
| N3 | 40.3(18) | 123(3) | 51.6(18) | -18.4(19) | -9.8(14) | 19.6(18) |
| N4 | 30.2(13) | 43.7(15) | 66.2(18) | -3.1(13) | 8.3(12) | -4.4(11) |
| N5 | 39.3(16) | 56.7(18) | 87(2) | 10.0(16) | 2.1(15) | -18.3(14) |
| N6 | 37.1(14) | 40.0(14) | 53.0(16) | 5.3(12) | 2.4(12) | -9.3(11) |
| N7 | 79(2) | 49.7(18) | 72(2) | -13.7(15) | 33.1(18) | -12.4(16) |
| N8 | 45.6(17) | 45.1(16) | 72(2) | -2.9(14) | 11.6(14) | 1.8(13) |
| N9 | 39.7(15) | 42.3(15) | 57.4(17) | 3.0(12) | 0.8(12) | -2.5(12) |
| N10 | 34.3(14) | 44.4(15) | 63.6(18) | 9.5(13) | 7.3(12) | -0.4(12) |
| N11 | 79(2) | 54.0(19) | 63(2) | 19.4(15) | 27.6(17) | 12.7(17) |
| N12 | 44.8(16) | 52.4(16) | 44.8(15) | 8.4(13) | -2.3(12) | 5.4(13) |
| N13 | 52.3(17) | 46.2(16) | 57.1(17) | 2.2(13) | 14.0(14) | -0.5(13) |
| N14 | 56.4(19) | 81(2) | 50.0(17) | 8.4(15) | 5.7(14) | -19.6(17) |
| N15 | 32.8(14) | 54.3(16) | 46.7(15) | -6.2(12) | 2.4(11) | 2.3(12) |
| C1 | 40.3(18) | 47.4(18) | 53.1(19) | 1.7(15) | 0.7(14) | -7.8(15) |
| C2 | 44.8(19) | 46.3(19) | 63(2) | 7.1(16) | 2.7(16) | -7.3(15) |
| C3 | 61(2) | 47(2) | 68(2) | 9.9(17) | -0.5(18) | -13.5(17) |
| C4 | 55(2) | 46(2) | 69(2) | 5.0(17) | -0.3(18) | -4.4(17) |
| C5 | 45.4(19) | 45.2(18) | 59(2) | -2.2(15) | 2.1(15) | -6.2(15) |
| C6 | 56(2) | 41.2(19) | 72(2) | -2.0(17) | 8.1(18) | -3.6(17) |
| C7 | 42.9(19) | 40.9(17) | 60(2) | -2.4(15) | 12.3(16) | 3.1(15) |
| C8 | 49(2) | 41.0(17) | 50.8(19) | -1.1(14) | 7.7(15) | 1.0(15) |
| C9 | 48.8(19) | 45.1(18) | 51.1(19) | -8.2(15) | 9.6(15) | -2.6(15) |
| C10 | 46.1(19) | 42.7(18) | 57(2) | -6.8(15) | 11.0(16) | 2.5(15) |
| C11 | 58(2) | 66(2) | 65(2) | -23.7(19) | 19.5(19) | -6.4(19) |
| C12 | 55(2) | 60(2) | 76(3) | -23(2) | 9.0(19) | -10.7(18) |
| C13 | 62(3) | 103(4) | 117(4) | -56(3) | 18(3) | -19(3) |
| C14 | 56(2) | 67(2) | 57(2) | -15.3(18) | 13.6(17) | -6.7(18) |
| C15 | 46.8(19) | 49.3(19) | 55(2) | -10.7(15) | 17.5(16) | 0.8(15) |
| C16 | 51(2) | 66(2) | 75(3) | -12(2) | 10.8(19) | 10.5(19) |
| C17 | 72(3) | 87(4) | 145(5) | -23(4) | 6(3) | 12(3) |
| C18 | 86(4) | 156(6) | 141(6) | -79(5) | 22(4) | 20(4) |
| C19 | 109(5) | 124(5) | 98(4) | -43(4) | 43(4) | -31(4) |
| C20 | 65(3) | 76(3) | 68(3) | -26(2) | 26(2) | -2(2) |
| C21 | 42.2(19) | 57(2) | 51.5(19) | -9.9(16) | 15.1(15) | -2.9(16) |
| C22 | 58(2) | 63(2) | 71(3) | -22(2) | -0.5(19) | 8.8(19) |
| C23 | 58(2) | 64(3) | 85(3) | -28(2) | 8(2) | -1(2) |
| C24 | 59(2) | 52(2) | 61(2) | -19.3(17) | 20.5(18) | -10.4(18) |
| C25 | 71(3) | 55(2) | 67(2) | 5.1(19) | 4(2) | -9(2) |
| C26 | 69(3) | 59(2) | 68(2) | -3.9(19) | 0(2) | -20(2) |
| C27 | 140(5) | 72(3) | 116(4) | 19(3) | -60(4) | -20(3) |
| C28 | 92(4) | 80(3) | 150(5) | -41(3) | -37(4) | -7(3) |
| C29 | 69(3) | 54(2) | 61(2) | -9.9(18) | 24.8(19) | -4.4(19) |
| C30 | 49(2) | 53(2) | 63(2) | -5.8(17) | 22.8(17) | -5.9(16) |
| C31 | 70(3) | 58(2) | 57(2) | -9.2(18) | 29.0(19) | -4.1(19) |
| C32 | 83(3) | 60(2) | 55(2) | -0.3(18) | 33(2) | -8(2) |
| C33 | 56(2) | 54(2) | 54(2) | 5.2(16) | 17.9(17) | -4.4(17) |
| C34 | 40.2(18) | 46.6(18) | 51.4(19) | -0.8(15) | 11.7(14) | -4.0(14) |
| C35 | 34.4(16) | 48.9(18) | 53.8(19) | -1.8(15) | 10.8(14) | -4.8(14) |
| C36 | 33.5(16) | 40.9(17) | 55(2) | 0.5(14) | 10.3(14) | -3.0(13) |
| C37 | 54(5) | 58(3) | 76(3) | 1(3) | 23(3) | -17(4) |
| C38 | 48(4) | 71(3) | 63(4) | 1(3) | 14(3) | -17(3) |
| C39 | 65(4) | 83(3) | 62(3) | 1(3) | 12(3) | -27(3) |
| C40 | 81(4) | 82(3) | 50(3) | 8(2) | 2(3) | -30(3) |
| C41 | 72(4) | 70(3) | 54(3) | 10(2) | -4(2) | -25(3) |
| C42 | 62(4) | 59(3) | 54(3) | 7(3) | 3(3) | -20(3) |
| C43 | 34.5(18) | 49(2) | 96(2) | 17.7(16) | 5.7(18) | -8.9(16) |
| C44 | 39.9(19) | 48(2) | 106(3) | 10.2(18) | 12(2) | -10.5(16) |
| C45 | 35.1(19) | 49(2) | 104(3) | 9(2) | 10.0(19) | -8.6(16) |
| C46 | 31.6(17) | 51(2) | 90(3) | 6(2) | 7.8(17) | -8.6(15) |
| C47 | 30.9(17) | 48(2) | 96(3) | 13(2) | 5.1(18) | -8.9(15) |
| C48 | 39.5(19) | 55(2) | 94(3) | 3(2) | 9.9(19) | -12.0(17) |
| C49 | 43(2) | 54(2) | 100(3) | 6(2) | 3(2) | -1.6(17) |
| C50 | 58(3) | 59(2) | 111(4) | -1(2) | 4(2) | -14(2) |
| C51 | 37.1(19) | 53(2) | 92(3) | 4.7(19) | 7.8(19) | -9.7(17) |
| C52 | 28.8(16) | 45.1(18) | 82(3) | -2.5(17) | 7.3(16) | -6.2(14) |
| C53 | 38.8(19) | 57(2) | 87(3) | -9(2) | 3.4(18) | -12.9(16) |
| C54 | 36.4(18) | 60(2) | 72(2) | -14.5(19) | -0.7(16) | -3.8(16) |
| C55 | 37.2(18) | 48.9(19) | 65(2) | -7.0(16) | 5.6(15) | -6.0(15) |
| C56 | 29.6(15) | 39.8(17) | 61(2) | -4.8(14) | 6.7(14) | 0.6(13) |
| C57 | 39.9(18) | 43.7(18) | 57(2) | -0.5(15) | 6.4(15) | -8.3(14) |
| C58 | 30.4(15) | 41.3(16) | 45.6(17) | 4.7(13) | 3.2(12) | -4.1(13) |
| C59 | 31.7(16) | 43.2(17) | 50.8(18) | -0.3(14) | 5.4(13) | -5.3(13) |
| C60 | 31.1(16) | 41.6(17) | 53.2(18) | -1.3(14) | 3.3(13) | -2.6(13) |
| C61 | 29.8(15) | 41.6(16) | 44.6(17) | 4.3(13) | 2.4(12) | -3.2(13) |
| C62 | 31.7(16) | 41.9(17) | 49.8(18) | 3.7(14) | -0.3(13) | -2.6(13) |
| C63 | 34.6(16) | 38.6(16) | 46.2(17) | 2.9(13) | 1.5(13) | -3.7(13) |
| C64 | 38.3(18) | 42.2(18) | 72(2) | -3.0(16) | 2.5(16) | -0.2(14) |
| C65 | 31.1(17) | 55(2) | 83(3) | -14.5(18) | 6.1(16) | -4.0(15) |
| C66 | 32.6(16) | 44.1(17) | 47.8(17) | 5.9(13) | -0.8(13) | -9.2(13) |
| C67 | 37.3(17) | 51.7(19) | 53.5(19) | 5.0(15) | 0.5(14) | -10.8(15) |
| C68 | 47(2) | 72(2) | 52(2) | -1.7(17) | 6.9(16) | -21.9(18) |
| C69 | 64(2) | 67(2) | 50(2) | 18.0(17) | -16.0(17) | -30(2) |
| C70 | 52(2) | 45.0(18) | 59(2) | 11.8(15) | -15.3(16) | -14.2(16) |
| C71 | 40.4(18) | 43.8(18) | 52.1(19) | 4.1(14) | -6.6(14) | -6.7(14) |
| C72 | 33.7(16) | 39.9(16) | 47.8(18) | 3.9(13) | -1.8(13) | 0.1(13) |
| C73 | 32.0(16) | 46.7(18) | 49.1(18) | 3.1(14) | 1.8(13) | -5.3(13) |
| C74 | 34.0(16) | 45.4(18) | 50.8(19) | 3.1(14) | -0.6(13) | 0.7(14) |
| C8AA | 38.8(18) | 45.1(18) | 49.3(18) | 6.5(14) | 1.2(14) | 3.5(14) |
| C76 | 39.9(18) | 57(2) | 50.5(19) | 7.7(16) | 3.7(14) | -8.4(15) |
| C77 | 34.8(17) | 56(2) | 56(2) | 6.7(16) | 0.2(14) | -10.5(15) |
| C78 | 59(2) | 100(3) | 57(2) | 19(2) | 4.5(19) | -29(2) |
| C79 | 42.5(19) | 62(2) | 54(2) | -0.9(16) | -5.9(15) | -9.6(16) |
| C80 | 43.1(19) | 50(2) | 62(2) | 5.1(16) | 5.1(16) | -7.2(15) |
| C81 | 30.3(16) | 40.2(17) | 61(2) | 7.1(15) | 5.8(14) | -2.3(13) |
| C82 | 42.7(18) | 46.8(18) | 55(2) | 0.3(15) | 5.2(15) | -6.6(15) |
| C83 | 37.7(17) | 49.8(19) | 47.1(18) | 7.7(15) | -3.1(14) | -8.7(14) |
| C84 | 28.1(15) | 47.2(18) | 47.8(17) | 2.4(14) | 3.1(13) | -9.2(13) |
| C85 | 32.9(16) | 49.3(18) | 49.6(18) | -1.3(14) | 0.4(13) | -6.8(14) |
| C86 | 33.0(16) | 46.7(18) | 54.0(19) | 4.7(15) | -0.6(14) | -9.5(14) |
| C87 | 40.9(19) | 60(2) | 72(2) | 5.6(18) | -13.5(17) | -7.9(17) |
| C88 | 66(3) | 60(2) | 64(2) | -7.9(19) | 3.5(19) | -1.3(19) |
| C89 | 28.4(15) | 55.3(19) | 50.3(18) | -3.0(15) | -3.5(13) | -4.5(14) |
| C90 | 33.1(17) | 64(2) | 63(2) | -6.8(17) | -7.3(15) | -7.2(16) |
| C91 | 44(2) | 66(2) | 91(3) | -14(2) | 0.9(19) | -14.5(18) |
| C92 | 43(2) | 76(3) | 80(3) | -14(2) | 10.2(19) | -12.2(19) |
| C93 | 47(2) | 64(2) | 68(2) | -15.7(19) | 10.5(17) | -5.8(17) |
| C94 | 32.6(17) | 62(2) | 56(2) | -11.6(16) | 3.8(14) | -4.1(15) |
| C95 | 32.0(16) | 44.6(17) | 45.6(17) | -7.0(13) | -0.6(13) | 1.8(13) |
| C96 | 40.7(18) | 56(2) | 54(2) | -2.9(16) | -6.4(15) | -6.3(16) |
| C97 | 53(2) | 53(2) | 51(2) | 0.0(16) | -7.1(16) | -1.5(17) |
| C98 | 40.9(18) | 41.9(17) | 54.9(19) | -1.4(15) | 10.5(15) | 2.3(14) |
| C99 | 32.1(16) | 45.6(18) | 58(2) | -1.8(15) | 4.0(14) | -0.3(14) |
| C100 | 30.9(16) | 49.4(18) | 47.9(18) | -2.2(14) | -0.8(13) | 0.5(13) |
| C101 | 91(3) | 77(3) | 58(2) | 14(2) | -25(2) | -17(3) |
| C102 | 43(2) | 58(2) | 77(3) | -0.8(19) | 2.0(18) | -12.7(17) |
| C103 | 44(2) | 63(2) | 73(2) | -13.6(19) | 13.5(18) | -2.7(18) |
| C104 | 48(2) | 58(2) | 50.8(19) | -3.1(16) | 15.5(15) | -5.8(16) |
| C105 | 50(2) | 68(2) | 51(2) | 3.4(17) | 12.3(16) | -5.2(18) |
| C106 | 53(2) | 71(3) | 57(2) | 14.7(19) | 8.2(17) | -5.5(19) |
| C107 | 67(3) | 65(2) | 48(2) | 7.8(17) | 11.9(17) | -15(2) |
| C108 | 49(2) | 64(2) | 39.1(17) | 1.6(15) | 10.9(14) | -9.1(17) |
| C109 | 49(2) | 66(2) | 46.7(19) | -0.3(17) | 9.2(15) | -3.7(17) |
| C110 | 58(2) | 73(3) | 45.8(19) | -2.9(18) | 10.5(17) | -18(2) |
| C111 | 53(2) | 71(2) | 48(2) | 9.0(18) | 8.5(16) | -16.8(19) |
| C112 | 44(2) | 70(2) | 54(2) | 3.5(18) | 6.8(16) | -9.3(18) |
| C113 | 48(2) | 63(2) | 52(2) | 5.2(17) | 4.2(16) | -1.5(17) |
| C114 | 47(2) | 58(2) | 62(2) | 12.0(19) | 13.7(17) | -9.2(17) |
| C115 | 61(3) | 58(2) | 72(3) | 8(2) | 30(2) | -5.6(19) |
| C116 | 68(3) | 61(2) | 57(2) | 3.9(18) | 15.4(19) | -15(2) |
| C117 | 90(3) | 90(3) | 75(3) | -8(3) | 25(3) | -27(3) |
| C118 | 45(2) | 89(3) | 70(3) | -11(2) | 10.6(18) | -7(2) |
| C119 | 31.0(16) | 48.1(18) | 44.8(17) | 0.2(14) | 1.9(13) | 2.4(13) |
| C120 | 33.7(16) | 54.1(19) | 46.3(18) | -6.9(15) | 0.4(13) | 0.0(14) |
| C121 | 28.4(16) | 65(2) | 51.0(19) | -9.4(16) | 2.1(14) | 5.0(15) |
| C122 | 30.1(17) | 70(2) | 55(2) | -21.9(18) | -6.9(14) | 14.3(16) |
| C123 | 36.7(19) | 54(2) | 73(2) | -17.5(18) | -7.1(16) | 8.1(15) |
| C124 | 36.5(17) | 45.7(18) | 59(2) | -4.5(15) | -0.3(15) | 1.8(14) |
| C125 | 43(2) | 56(2) | 87(3) | -7(2) | 3.1(18) | -10.5(17) |
| C126 | 42.1(19) | 78(3) | 67(2) | -21(2) | 10.6(17) | -16.0(18) |
| C127 | 32.5(18) | 115(3) | 67.5(18) | -42.6(15) | -14.6(16) | 25.7(17) |
| C128 | 38.8(19) | 146(3) | 56.0(19) | -28.8(16) | -10.8(16) | 40(2) |
| C129 | 57(3) | 201(4) | 62.4(19) | -43(2) | -8.7(19) | 56(2) |
| C130 | 63(3) | 235(5) | 90(2) | -93(2) | -35(2) | 70(3) |
| C131 | 59(3) | 191(4) | 120(3) | -108(2) | -30(2) | 48(3) |
| C132 | 40(2) | 145(3) | 113(3) | -86(2) | -23.9(19) | 29.5(19) |
| C133 | 29.5(18) | 89(3) | 56(2) | -26(2) | -7.6(15) | 17.9(19) |
| C134 | 43(2) | 87(3) | 56(2) | -25(2) | -11.1(17) | 16(2) |
| C135 | 35.0(19) | 96(3) | 58(2) | -28(2) | -4.4(16) | 6(2) |
| C136 | 36(2) | 97(3) | 48(2) | -21(2) | -8.7(16) | 19(2) |
| C137 | 46(2) | 99(3) | 52(2) | -11(2) | -4.0(17) | 18(2) |
| C138 | 36(2) | 94(3) | 71(3) | -14(2) | 0.2(17) | 10(2) |
| C139 | 54(3) | 113(4) | 122(4) | -42(3) | -21(3) | 6(3) |
| C140 | 72(3) | 112(4) | 86(3) | 7(3) | -12(3) | 10(3) |
| C141 | 45(2) | 99(3) | 54(2) | -22(2) | -9.8(17) | 20(2) |
| C143 | 48(2) | 146(5) | 68(3) | -27(3) | -8(2) | 26(3) |
| C144 | 38(2) | 211(7) | 90(4) | -34(4) | -10(2) | 32(3) |
| C145 | 49(3) | 207(7) | 80(4) | -43(4) | -19(2) | 42(3) |
| C146 | 44(2) | 192(6) | 73(3) | -60(4) | -18(2) | 50(3) |
| C147 | 56(3) | 264(9) | 75(3) | -75(5) | -18(3) | 52(4) |
| C148 | 60(3) | 171(3) | 50(2) | -34(2) | -8.3(16) | 31(2) |
| C149 | 106(4) | 185(4) | 93(3) | -78(3) | 18(3) | -26(3) |
| C150 | 100(3) | 138(3) | 84(3) | -53(2) | 26(2) | -33(3) |
| C151 | 57(2) | 81(2) | 41.5(16) | -4.3(13) | -4.2(13) | 14.7(17) |
| C152 | 92(3) | 74(2) | 53.8(17) | 11.3(14) | 16.1(18) | 28.1(18) |
| C153 | 87(3) | 111(3) | 46.0(16) | 16.3(15) | 9.1(17) | 49(2) |
| C154 | 176(6) | 135(4) | 104(4) | 43(3) | 82(4) | 58(3) |
| C156 | 58(2) | 56(2) | 36.7(17) | 1.1(15) | 0.2(15) | 3.1(17) |
| C157 | 65(2) | 54(2) | 48(2) | -0.6(16) | -7.5(17) | 4.8(18) |
| C158 | 87(3) | 66(3) | 51(2) | -4.4(19) | -9(2) | -7(2) |
| C159 | 112(4) | 72(3) | 39(2) | -0.1(18) | 0(2) | -20(3) |
| C160 | 92(3) | 60(2) | 46(2) | 3.1(17) | 7(2) | -23(2) |
| C161 | 79(3) | 52(2) | 49(2) | -0.7(16) | -1.8(18) | -5.7(19) |
| C162 | 55(2) | 55(2) | 43.8(18) | 8.9(15) | 9.5(15) | 0.5(17) |
| C163 | 57(2) | 64(2) | 36.9(17) | 8.5(15) | 8.3(15) | -3.3(18) |
| C164 | 57(2) | 64(2) | 40.2(18) | 5.5(16) | 11.2(16) | 11.9(19) |
| C165 | 68(3) | 54(2) | 54(2) | 15.8(17) | 23.9(18) | 4.8(19) |
| C166 | 68(3) | 57(2) | 78(3) | 16(2) | 32(2) | 0(2) |
| C167 | 57(2) | 56(2) | 70(3) | 5.7(19) | 17.5(19) | 6.6(19) |
| C168 | 83(3) | 64(3) | 133(5) | 13(3) | 39(3) | -13(3) |
| C169 | 57(2) | 78(3) | 56(2) | 2.8(19) | 0.6(18) | 10(2) |
| C170 | 54(2) | 50(2) | 51(2) | 5.5(16) | 1.4(16) | 10.5(17) |
| C171 | 66(2) | 58(2) | 50(2) | 2.7(17) | -0.1(18) | 11.3(19) |
| C172 | 125(4) | 60(3) | 62(3) | -8(2) | -6(3) | 25(3) |
| C173 | 176(6) | 71(3) | 60(3) | -9(2) | -15(3) | 20(3) |
| C174 | 123(4) | 74(3) | 49(2) | -1(2) | -4(2) | 17(3) |
| C175 | 71(3) | 61(2) | 48(2) | 7.4(18) | 10.3(18) | 14(2) |
| C176 | 59(2) | 52(2) | 48(2) | 2.1(16) | 8.0(16) | 8.5(17) |
| C177 | 85(3) | 65(3) | 48(2) | 15.8(18) | 21(2) | 16(2) |
| S600 | 56.4(6) | 54.4(5) | 70.4(6) | 7.0(4) | 1.2(5) | 0.9(4) |
| S200 | 182(2) | 230(3) | 160(2) | -70(2) | 22.4(19) | -79(2) |
| S800 | 54.4(9) | 51.4(8) | 64.2(9) | -9.0(6) | 19.3(6) | -12.0(6) |
| S500 | 64.6(8) | 208.6(19) | 54.7(7) | -34.0(9) | 7.6(5) | 12.9(9) |
| S007 | 152.4(18) | 120.5(14) | 144.8(17) | -42.5(13) | -55.7(14) | 30.9(13) |
| S300 | 57.7(7) | 105.2(11) | 176.3(16) | 39.5(11) | 1.5(9) | -21.1(7) |
| S700 | 120.6(14) | 126.0(14) | 121.5(13) | -16.0(11) | -7.3(11) | 12.3(11) |
| C200 | 100(5) | 199(6) | 145(6) | 87(5) | 11(4) | 2(5) |
| C301 | 61(3) | 93(4) | 156(6) | 9(4) | 26(3) | -3(3) |
| C701 | 196(10) | 241(12) | 156(8) | -63(8) | -48(7) | 89(9) |
| C1AA | 125(5) | 144(5) | 51(3) | -4(3) | 22(3) | -44(4) |
| C501 | 149(7) | 216(9) | 69(4) | -8(5) | 14(4) | 65(6) |
| C801 | 72(5) | 71(4) | 63(3) | -9(3) | 17(2) | 5(3) |
| C600 | 61(2) | 68(3) | 68(3) | 15(2) | -0.8(19) | -5(2) |
| C800 | 67(4) | 59(2) | 44(3) | -11(2) | 7(3) | -11(3) |
| O800 | 59(3) | 58(2) | 72(2) | 0.2(18) | 28.1(17) | 1(2) |
| C201 | 111(5) | 86(4) | 141(5) | 15(4) | -10(4) | -16(3) |
| O05I | 223(6) | 206(6) | 160(4) | 57(4) | -133(4) | -102(5) |
| C500 | 69(4) | 406(18) | 165(8) | -129(10) | 43(5) | -42(7) |
| C601 | 170(7) | 146(6) | 92(4) | -36(4) | -5(4) | -46(5) |
| O600 | 63.2(18) | 59.3(17) | 97(2) | 20.7(15) | 22.5(15) | 1.6(14) |
| O200 | 218(7) | 258(8) | 195(6) | -75(6) | 88(6) | -18(6) |
| C0AA | 203(8) | 100(5) | 150(6) | -38(4) | -63(6) | 12(5) |
| O300 | 67(3) | 272(7) | 173(5) | 35(5) | 51(3) | 29(4) |
| O700 | 146(5) | 95(3) | 260(7) | -35(4) | -39(5) | 12(3) |
| O500 | 62.0(19) | 162(4) | 60.1(18) | -27(2) | -10.4(14) | 15(2) |
| C071 | 168(6) | 153(7) | 136(6) | -25(5) | -42(5) | 40(5) |
| C300 | 73(3) | 87(4) | 156(6) | 1(4) | 4(3) | -15(3) |
| C155 | 231(9) | 252(8) | 179(7) | -153(6) | 43(6) | -85(7) |
| C5AA | 45(2) | 113(4) | 66(3) | -27(2) | -14.0(19) | 33(2) |
| S50 | 119.2(16) | 194(2) | 177(2) | -31.1(19) | 0.7(15) | -5.1(15) |
| O50A | 108(4) | 263(5) | 225(6) | -66(5) | -13(4) | 21(3) |
| C2AA | 188(7) | 209(9) | 163(7) | 9(6) | -40(5) | -8(6) |
| C4AA | 200(10) | 289(7) | 199(10) | -25(8) | 42(7) | -83(6) |
| C37A | 49(11) | 49(8) | 124(13) | 21(8) | 23(8) | 0(7) |
| C38A | 41(6) | 87(8) | 50(7) | -1(6) | 10(5) | -27(5) |
| C39A | 62(7) | 114(10) | 46(6) | 2(6) | 5(5) | -44(7) |
| C40A | 104(10) | 123(12) | 57(7) | -4(7) | 8(7) | -77(9) |
| C41A | 99(9) | 105(10) | 51(6) | 1(6) | 10(6) | -64(8) |
| C42A | 79(8) | 57(7) | 40(6) | 19(6) | -4(6) | -23(6) |
| S8 | 57(2) | 76(2) | 62.0(19) | -12.4(15) | 11.0(14) | -7.5(16) |
| C75 | 93(12) | 81(10) | 64(10) | 3(8) | -11(9) | -9(9) |
| C1A | 92(10) | 132(14) | 93(14) | -1(13) | -2(12) | 16(10) |
| O13 | 112(8) | 77(6) | 81(7) | -12(5) | 45(6) | -4(6) |

Table 4 Bond Lengths for exp\_2788.

| Atom | Atom | Length/Å |  | Atom | Atom | Length/Å |
| --- | --- | --- | --- | --- | --- | --- |
| O1 | C141 | 1.222(5) |  | C84 | C85 | 1.390(5) |
| O2 | C147 | 1.223(6) |  | C84 | C89 | 1.535(4) |
| O3 | C51 | 1.235(5) |  | C85 | C86 | 1.401(4) |
| O4 | C57 | 1.227(4) |  | C86 | C87 | 1.505(5) |
| O5 | C6 | 1.234(4) |  | C89 | C90 | 1.550(5) |
| O6 | C80 | 1.227(4) |  | C89 | C94 | 1.553(5) |
| O7 | C170 | 1.229(4) |  | C89 | C95 | 1.525(5) |
| O8 | C177 | 1.228(5) |  | C90 | C91 | 1.499(6) |
| O9 | C103 | 1.230(5) |  | C91 | C92 | 1.500(6) |
| O10 | C110 | 1.247(5) |  | C92 | C93 | 1.512(6) |
| O11 | C36 | 1.229(4) |  | C93 | C94 | 1.513(5) |
| O12 | C29 | 1.218(5) |  | C95 | C96 | 1.392(5) |
| N1 | C146 | 1.335(5) |  | C95 | C100 | 1.393(5) |
| N1 | C5AA | 1.329(6) |  | C96 | C97 | 1.376(5) |
| N2 | C147 | 1.347(7) |  | C97 | C98 | 1.398(5) |
| N2 | C148 | 1.423(6) |  | C97 | C101 | 1.514(5) |
| N3 | C136 | 1.437(5) |  | C98 | C99 | 1.400(5) |
| N3 | C141 | 1.327(5) |  | C99 | C100 | 1.379(5) |
| N4 | C52 | 1.337(4) |  | C99 | C102 | 1.494(5) |
| N4 | C56 | 1.344(4) |  | C103 | C104 | 1.485(6) |
| N5 | C46 | 1.417(5) |  | C104 | C105 | 1.398(5) |
| N5 | C51 | 1.338(6) |  | C104 | C109 | 1.387(5) |
| N6 | C57 | 1.331(4) |  | C105 | C106 | 1.384(6) |
| N6 | C58 | 1.432(4) |  | C106 | C107 | 1.384(6) |
| N7 | C24 | 1.422(5) |  | C107 | C108 | 1.380(5) |
| N7 | C29 | 1.336(5) |  | C108 | C109 | 1.385(5) |
| N8 | C6 | 1.332(5) |  | C108 | C110 | 1.492(5) |
| N8 | C7 | 1.426(5) |  | C111 | C112 | 1.366(6) |
| N9 | C1 | 1.339(4) |  | C111 | C116 | 1.395(6) |
| N9 | C5 | 1.342(4) |  | C112 | C113 | 1.382(6) |
| N10 | C80 | 1.354(4) |  | C112 | C118 | 1.509(5) |
| N10 | C81 | 1.441(4) |  | C113 | C114 | 1.389(5) |
| N11 | C165 | 1.427(5) |  | C114 | C115 | 1.384(6) |
| N11 | C177 | 1.327(5) |  | C114 | C37A | 1.543(13) |
| N12 | C8AA | 1.428(4) |  | C115 | C116 | 1.383(6) |
| N12 | C170 | 1.329(5) |  | C116 | C117 | 1.499(7) |
| N13 | C98 | 1.430(4) |  | C119 | C120 | 1.385(5) |
| N13 | C103 | 1.329(5) |  | C119 | C124 | 1.395(5) |
| N14 | C110 | 1.335(5) |  | C120 | C121 | 1.396(5) |
| N14 | C111 | 1.434(5) |  | C120 | C126 | 1.512(5) |
| N15 | C36 | 1.351(4) |  | C121 | C122 | 1.377(5) |
| N15 | C119 | 1.435(4) |  | C122 | C123 | 1.389(6) |
| C1 | C2 | 1.396(5) |  | C122 | C127 | 1.539(5) |
| C1 | C80 | 1.490(5) |  | C123 | C124 | 1.389(5) |
| C2 | C3 | 1.370(5) |  | C124 | C125 | 1.496(5) |
| C3 | C4 | 1.382(6) |  | C127 | C128 | 1.512(8) |
| C4 | C5 | 1.386(5) |  | C127 | C132 | 1.579(7) |
| C5 | C6 | 1.493(5) |  | C127 | C133 | 1.540(5) |
| C7 | C8 | 1.398(5) |  | C128 | C129 | 1.550(6) |
| C7 | C12 | 1.388(5) |  | C129 | C130 | 1.527(11) |
| C8 | C9 | 1.391(5) |  | C130 | C131 | 1.446(11) |
| C8 | C14 | 1.498(5) |  | C131 | C132 | 1.542(7) |
| C9 | C10 | 1.382(5) |  | C133 | C134 | 1.384(6) |
| C10 | C11 | 1.399(6) |  | C133 | C138 | 1.373(6) |
| C10 | C15 | 1.542(5) |  | C134 | C135 | 1.403(5) |
| C11 | C12 | 1.402(6) |  | C135 | C136 | 1.348(7) |
| C12 | C13 | 1.513(6) |  | C135 | C139 | 1.520(7) |
| C15 | C16 | 1.540(5) |  | C136 | C137 | 1.394(7) |
| C15 | C20 | 1.542(5) |  | C137 | C138 | 1.400(5) |
| C15 | C21 | 1.544(5) |  | C137 | C140 | 1.505(7) |
| C16 | C17 | 1.489(7) |  | C141 | C5AA | 1.519(5) |
| C17 | C18 | 1.495(9) |  | C143 | C144 | 1.384(7) |
| C18 | C19 | 1.527(10) |  | C143 | C5AA | 1.383(7) |
| C19 | C20 | 1.465(7) |  | C144 | C145 | 1.358(8) |
| C21 | C22 | 1.367(6) |  | C145 | C146 | 1.408(7) |
| C21 | C26 | 1.382(5) |  | C146 | C147 | 1.488(8) |
| C22 | C23 | 1.394(6) |  | C148 | C149 | 1.267(10) |
| C23 | C24 | 1.399(6) |  | C148 | C153 | 1.330(9) |
| C23 | C28 | 1.493(7) |  | C149 | C150 | 1.415(7) |
| C24 | C25 | 1.400(6) |  | C149 | C155 | 1.510(8) |
| C25 | C26 | 1.395(6) |  | C150 | C151 | 1.306(7) |
| C25 | C27 | 1.491(7) |  | C151 | C152 | 1.405(7) |
| C29 | C30 | 1.519(5) |  | C151 | C156 | 1.552(5) |
| C30 | C31 | 1.387(5) |  | C152 | C153 | 1.377(7) |
| C30 | C34 | 1.387(5) |  | C153 | C154 | 1.571(9) |
| C31 | C32 | 1.367(6) |  | C156 | C157 | 1.554(5) |
| C32 | C33 | 1.375(5) |  | C156 | C161 | 1.524(6) |
| C33 | C35 | 1.368(5) |  | C156 | C162 | 1.530(5) |
| C34 | C35 | 1.388(5) |  | C157 | C158 | 1.521(6) |
| C35 | C36 | 1.498(5) |  | C158 | C159 | 1.507(7) |
| C37 | C38 | 1.531(9) |  | C159 | C160 | 1.520(7) |
| C37 | C42 | 1.547(9) |  | C160 | C161 | 1.555(5) |
| C37 | C43 | 1.545(9) |  | C162 | C163 | 1.401(5) |
| C37 | C114 | 1.547(9) |  | C162 | C167 | 1.373(6) |
| C38 | C39 | 1.505(8) |  | C163 | C164 | 1.403(5) |
| C39 | C40 | 1.498(8) |  | C164 | C165 | 1.374(6) |
| C40 | C41 | 1.508(8) |  | C164 | C169 | 1.520(6) |
| C41 | C42 | 1.495(8) |  | C165 | C166 | 1.390(6) |
| C43 | C44 | 1.369(7) |  | C166 | C167 | 1.410(5) |
| C43 | C48 | 1.419(5) |  | C166 | C168 | 1.508(7) |
| C43 | C37A | 1.595(13) |  | C170 | C171 | 1.493(5) |
| C44 | C45 | 1.387(6) |  | C171 | C172 | 1.380(6) |
| C45 | C46 | 1.391(5) |  | C171 | C176 | 1.405(5) |
| C45 | C50 | 1.514(7) |  | C172 | C173 | 1.377(7) |
| C46 | C47 | 1.418(6) |  | C173 | C174 | 1.377(7) |
| C47 | C48 | 1.373(6) |  | C174 | C175 | 1.369(7) |
| C47 | C49 | 1.513(5) |  | C175 | C176 | 1.379(5) |
| C51 | C52 | 1.500(5) |  | C175 | C177 | 1.507(5) |
| C52 | C53 | 1.397(6) |  | S600 | C600 | 1.759(5) |
| C53 | C54 | 1.381(6) |  | S600 | C601 | 1.811(7) |
| C54 | C55 | 1.368(5) |  | S600 | O600 | 1.499(3) |
| C55 | C56 | 1.398(5) |  | S200 | C200 | 1.753(10) |
| C56 | C57 | 1.494(5) |  | S200 | C201 | 1.754(8) |
| C58 | C59 | 1.384(5) |  | S200 | O200 | 1.541(8) |
| C58 | C63 | 1.415(4) |  | S800 | C801 | 1.784(9) |
| C59 | C60 | 1.387(5) |  | S800 | C800 | 1.788(8) |
| C59 | C65 | 1.512(4) |  | S800 | O800 | 1.498(4) |
| C60 | C61 | 1.392(4) |  | S500 | C501 | 1.771(10) |
| C61 | C62 | 1.401(5) |  | S500 | C500 | 1.771(9) |
| C61 | C66 | 1.531(5) |  | S500 | O500 | 1.480(4) |
| C62 | C63 | 1.384(5) |  | S007 | O05I | 1.311(7) |
| C63 | C64 | 1.506(5) |  | S007 | C0AA | 1.715(8) |
| C66 | C67 | 1.542(5) |  | S007 | C071 | 1.856(10) |
| C66 | C71 | 1.543(4) |  | S300 | C301 | 1.736(6) |
| C66 | C72 | 1.547(4) |  | S300 | O300 | 1.491(6) |
| C67 | C68 | 1.536(5) |  | S300 | C300 | 1.726(6) |
| C68 | C69 | 1.533(6) |  | S700 | C701 | 1.712(10) |
| C69 | C70 | 1.520(6) |  | S700 | C1AA | 1.588(7) |
| C70 | C71 | 1.529(5) |  | S700 | O700 | 1.429(5) |
| C72 | C73 | 1.405(5) |  | S50 | O50A | 1.444(7) |
| C72 | C77 | 1.390(5) |  | S50 | C2AA | 1.670(11) |
| C73 | C74 | 1.392(5) |  | S50 | C4AA | 1.542(13) |
| C74 | C8AA | 1.373(5) |  | C37A | C38A | 1.555(12) |
| C74 | C79 | 1.507(5) |  | C37A | C42A | 1.562(11) |
| C8AA | C76 | 1.415(5) |  | C38A | C39A | 1.524(11) |
| C76 | C77 | 1.379(5) |  | C39A | C40A | 1.527(11) |
| C76 | C78 | 1.501(5) |  | C40A | C41A | 1.507(11) |
| C81 | C82 | 1.410(5) |  | C41A | C42A | 1.499(12) |
| C81 | C86 | 1.364(5) |  | S8 | C75 | 1.742(15) |
| C82 | C83 | 1.385(5) |  | S8 | C1A | 1.74(3) |
| C82 | C88 | 1.504(5) |  | S8 | O13 | 1.424(9) |
| C83 | C84 | 1.384(5) |  |  |  |  |

Table 5 Bond Angles for exp\_2788.

| Atom | Atom | Atom | Angle/˚ |  | Atom | Atom | Atom | Angle/˚ |
| --- | --- | --- | --- | --- | --- | --- | --- | --- |
| C5AA | N1 | C146 | 117.4(4) |  | C96 | C95 | C100 | 117.1(3) |
| C147 | N2 | C148 | 122.5(4) |  | C100 | C95 | C89 | 118.8(3) |
| C141 | N3 | C136 | 125.4(3) |  | C97 | C96 | C95 | 122.6(3) |
| C52 | N4 | C56 | 117.8(3) |  | C96 | C97 | C98 | 118.4(3) |
| C51 | N5 | C46 | 126.1(3) |  | C96 | C97 | C101 | 120.7(4) |
| C57 | N6 | C58 | 123.1(3) |  | C98 | C97 | C101 | 120.8(4) |
| C29 | N7 | C24 | 122.0(3) |  | C97 | C98 | N13 | 119.5(3) |
| C6 | N8 | C7 | 123.3(3) |  | C97 | C98 | C99 | 121.1(3) |
| C1 | N9 | C5 | 117.8(3) |  | C99 | C98 | N13 | 119.1(3) |
| C80 | N10 | C81 | 120.7(3) |  | C98 | C99 | C102 | 121.3(3) |
| C177 | N11 | C165 | 124.1(3) |  | C100 | C99 | C98 | 117.9(3) |
| C170 | N12 | C8AA | 123.5(3) |  | C100 | C99 | C102 | 120.7(3) |
| C103 | N13 | C98 | 125.2(3) |  | C99 | C100 | C95 | 122.8(3) |
| C110 | N14 | C111 | 122.9(3) |  | O9 | C103 | N13 | 122.4(4) |
| C36 | N15 | C119 | 123.6(3) |  | O9 | C103 | C104 | 121.6(3) |
| N9 | C1 | C2 | 122.8(3) |  | N13 | C103 | C104 | 115.9(3) |
| N9 | C1 | C80 | 118.7(3) |  | C105 | C104 | C103 | 121.7(3) |
| C2 | C1 | C80 | 118.5(3) |  | C109 | C104 | C103 | 118.8(3) |
| C3 | C2 | C1 | 118.3(3) |  | C109 | C104 | C105 | 119.3(4) |
| C2 | C3 | C4 | 119.9(3) |  | C106 | C105 | C104 | 119.6(4) |
| C3 | C4 | C5 | 118.3(3) |  | C107 | C106 | C105 | 120.1(4) |
| N9 | C5 | C4 | 123.0(3) |  | C108 | C107 | C106 | 120.9(4) |
| N9 | C5 | C6 | 117.0(3) |  | C107 | C108 | C109 | 119.0(4) |
| C4 | C5 | C6 | 119.9(3) |  | C107 | C108 | C110 | 121.9(4) |
| O5 | C6 | N8 | 123.0(4) |  | C109 | C108 | C110 | 119.0(3) |
| O5 | C6 | C5 | 121.0(3) |  | C108 | C109 | C104 | 121.0(3) |
| N8 | C6 | C5 | 116.0(3) |  | O10 | C110 | N14 | 123.3(4) |
| C8 | C7 | N8 | 118.2(3) |  | O10 | C110 | C108 | 120.1(3) |
| C12 | C7 | N8 | 120.3(4) |  | N14 | C110 | C108 | 116.6(3) |
| C12 | C7 | C8 | 121.5(3) |  | C112 | C111 | N14 | 119.1(3) |
| C7 | C8 | C14 | 120.7(3) |  | C112 | C111 | C116 | 121.3(4) |
| C9 | C8 | C7 | 117.7(3) |  | C116 | C111 | N14 | 119.6(4) |
| C9 | C8 | C14 | 121.6(4) |  | C111 | C112 | C113 | 118.8(4) |
| C10 | C9 | C8 | 123.0(4) |  | C111 | C112 | C118 | 121.5(4) |
| C9 | C10 | C11 | 117.9(3) |  | C113 | C112 | C118 | 119.7(4) |
| C9 | C10 | C15 | 119.4(4) |  | C112 | C113 | C114 | 122.5(4) |
| C11 | C10 | C15 | 122.8(3) |  | C113 | C114 | C37 | 116.7(5) |
| C10 | C11 | C12 | 121.1(4) |  | C113 | C114 | C37A | 124.3(7) |
| C7 | C12 | C11 | 118.8(4) |  | C115 | C114 | C37 | 126.4(5) |
| C7 | C12 | C13 | 121.6(4) |  | C115 | C114 | C113 | 116.9(4) |
| C11 | C12 | C13 | 119.6(4) |  | C115 | C114 | C37A | 118.7(6) |
| C10 | C15 | C20 | 113.6(3) |  | C116 | C115 | C114 | 122.5(4) |
| C10 | C15 | C21 | 106.0(3) |  | C111 | C116 | C117 | 120.2(4) |
| C16 | C15 | C10 | 108.3(3) |  | C115 | C116 | C111 | 118.2(4) |
| C16 | C15 | C20 | 107.9(3) |  | C115 | C116 | C117 | 121.6(4) |
| C16 | C15 | C21 | 112.8(4) |  | C120 | C119 | N15 | 119.4(3) |
| C20 | C15 | C21 | 108.4(3) |  | C120 | C119 | C124 | 121.1(3) |
| C17 | C16 | C15 | 115.3(4) |  | C124 | C119 | N15 | 119.5(3) |
| C16 | C17 | C18 | 114.6(6) |  | C119 | C120 | C121 | 118.5(3) |
| C17 | C18 | C19 | 108.8(5) |  | C119 | C120 | C126 | 121.2(3) |
| C20 | C19 | C18 | 110.5(5) |  | C121 | C120 | C126 | 120.3(3) |
| C19 | C20 | C15 | 115.7(4) |  | C122 | C121 | C120 | 122.2(3) |
| C22 | C21 | C15 | 123.5(3) |  | C121 | C122 | C123 | 117.5(3) |
| C22 | C21 | C26 | 118.1(4) |  | C121 | C122 | C127 | 119.6(4) |
| C26 | C21 | C15 | 118.3(4) |  | C123 | C122 | C127 | 122.8(4) |
| C21 | C22 | C23 | 122.1(4) |  | C124 | C123 | C122 | 122.4(4) |
| C22 | C23 | C24 | 119.0(4) |  | C119 | C124 | C125 | 121.2(3) |
| C22 | C23 | C28 | 119.4(4) |  | C123 | C124 | C119 | 118.1(3) |
| C24 | C23 | C28 | 121.6(4) |  | C123 | C124 | C125 | 120.7(4) |
| C23 | C24 | N7 | 121.3(4) |  | C122 | C127 | C132 | 110.2(4) |
| C23 | C24 | C25 | 120.2(4) |  | C122 | C127 | C133 | 107.8(3) |
| C25 | C24 | N7 | 118.5(4) |  | C128 | C127 | C122 | 107.8(4) |
| C24 | C25 | C27 | 121.8(4) |  | C128 | C127 | C132 | 108.3(4) |
| C26 | C25 | C24 | 118.0(4) |  | C128 | C127 | C133 | 114.1(4) |
| C26 | C25 | C27 | 120.2(5) |  | C133 | C127 | C132 | 108.6(4) |
| C21 | C26 | C25 | 122.6(4) |  | C127 | C128 | C129 | 112.8(5) |
| O12 | C29 | N7 | 122.7(4) |  | C130 | C129 | C128 | 112.6(6) |
| O12 | C29 | C30 | 119.7(3) |  | C131 | C130 | C129 | 110.4(5) |
| N7 | C29 | C30 | 117.5(3) |  | C130 | C131 | C132 | 113.5(5) |
| C31 | C30 | C29 | 123.9(3) |  | C131 | C132 | C127 | 109.0(5) |
| C34 | C30 | C29 | 116.7(3) |  | C134 | C133 | C127 | 119.5(4) |
| C34 | C30 | C31 | 119.3(3) |  | C138 | C133 | C127 | 123.0(4) |
| C32 | C31 | C30 | 119.7(4) |  | C138 | C133 | C134 | 117.3(3) |
| C31 | C32 | C33 | 121.2(3) |  | C133 | C134 | C135 | 121.2(4) |
| C35 | C33 | C32 | 119.6(4) |  | C134 | C135 | C139 | 117.9(4) |
| C30 | C34 | C35 | 120.0(3) |  | C136 | C135 | C134 | 119.8(5) |
| C33 | C35 | C34 | 120.0(3) |  | C136 | C135 | C139 | 122.2(4) |
| C33 | C35 | C36 | 122.1(3) |  | C135 | C136 | N3 | 121.1(5) |
| C34 | C35 | C36 | 117.9(3) |  | C135 | C136 | C137 | 121.2(4) |
| O11 | C36 | N15 | 123.5(3) |  | C137 | C136 | N3 | 117.5(4) |
| O11 | C36 | C35 | 122.5(3) |  | C136 | C137 | C138 | 117.5(4) |
| N15 | C36 | C35 | 114.0(3) |  | C136 | C137 | C140 | 121.6(4) |
| C38 | C37 | C42 | 108.1(6) |  | C138 | C137 | C140 | 120.9(5) |
| C38 | C37 | C43 | 101.6(6) |  | C133 | C138 | C137 | 122.9(5) |
| C38 | C37 | C114 | 118.0(7) |  | O1 | C141 | N3 | 125.5(4) |
| C43 | C37 | C42 | 107.0(6) |  | O1 | C141 | C5AA | 121.0(4) |
| C43 | C37 | C114 | 105.0(5) |  | N3 | C141 | C5AA | 113.5(4) |
| C114 | C37 | C42 | 115.6(7) |  | C5AA | C143 | C144 | 117.9(5) |
| C39 | C38 | C37 | 110.1(6) |  | C145 | C144 | C143 | 120.4(5) |
| C40 | C39 | C38 | 113.0(6) |  | C144 | C145 | C146 | 117.3(4) |
| C39 | C40 | C41 | 110.9(6) |  | N1 | C146 | C145 | 123.4(5) |
| C42 | C41 | C40 | 110.2(6) |  | N1 | C146 | C147 | 117.5(4) |
| C41 | C42 | C37 | 110.4(7) |  | C145 | C146 | C147 | 119.0(4) |
| C44 | C43 | C37 | 121.7(5) |  | O2 | C147 | N2 | 122.3(5) |
| C44 | C43 | C48 | 117.3(4) |  | O2 | C147 | C146 | 121.2(5) |
| C44 | C43 | C37A | 129.2(6) |  | N2 | C147 | C146 | 115.9(4) |
| C48 | C43 | C37 | 120.4(5) |  | C149 | C148 | N2 | 125.2(7) |
| C48 | C43 | C37A | 113.1(6) |  | C149 | C148 | C153 | 116.6(5) |
| C43 | C44 | C45 | 123.8(4) |  | C153 | C148 | N2 | 117.9(7) |
| C44 | C45 | C46 | 117.8(5) |  | C148 | C149 | C150 | 123.9(7) |
| C44 | C45 | C50 | 121.4(4) |  | C148 | C149 | C155 | 117.3(6) |
| C46 | C45 | C50 | 120.8(4) |  | C150 | C149 | C155 | 118.8(7) |
| N5 | C46 | C47 | 116.6(3) |  | C151 | C150 | C149 | 121.4(6) |
| C45 | C46 | N5 | 122.6(4) |  | C150 | C151 | C152 | 114.8(4) |
| C45 | C46 | C47 | 120.6(4) |  | C150 | C151 | C156 | 125.5(5) |
| C46 | C47 | C49 | 120.5(4) |  | C152 | C151 | C156 | 119.8(4) |
| C48 | C47 | C46 | 119.1(4) |  | C153 | C152 | C151 | 120.9(6) |
| C48 | C47 | C49 | 120.5(4) |  | C148 | C153 | C152 | 121.9(6) |
| C47 | C48 | C43 | 121.2(5) |  | C148 | C153 | C154 | 121.0(5) |
| O3 | C51 | N5 | 124.9(4) |  | C152 | C153 | C154 | 117.1(7) |
| O3 | C51 | C52 | 120.8(4) |  | C151 | C156 | C157 | 106.6(3) |
| N5 | C51 | C52 | 114.3(3) |  | C161 | C156 | C151 | 110.7(4) |
| N4 | C52 | C51 | 116.4(4) |  | C161 | C156 | C157 | 106.3(3) |
| N4 | C52 | C53 | 123.3(3) |  | C161 | C156 | C162 | 112.2(3) |
| C53 | C52 | C51 | 120.3(3) |  | C162 | C156 | C151 | 109.4(3) |
| C54 | C53 | C52 | 118.2(4) |  | C162 | C156 | C157 | 111.4(3) |
| C55 | C54 | C53 | 119.1(4) |  | C158 | C157 | C156 | 112.9(3) |
| C54 | C55 | C56 | 119.6(3) |  | C159 | C158 | C157 | 111.9(4) |
| N4 | C56 | C55 | 122.0(3) |  | C158 | C159 | C160 | 111.3(3) |
| N4 | C56 | C57 | 117.6(3) |  | C159 | C160 | C161 | 110.8(4) |
| C55 | C56 | C57 | 120.4(3) |  | C156 | C161 | C160 | 112.1(3) |
| O4 | C57 | N6 | 123.7(3) |  | C163 | C162 | C156 | 121.7(4) |
| O4 | C57 | C56 | 119.4(3) |  | C167 | C162 | C156 | 121.3(3) |
| N6 | C57 | C56 | 116.9(3) |  | C167 | C162 | C163 | 116.9(3) |
| C59 | C58 | N6 | 120.0(3) |  | C162 | C163 | C164 | 121.7(4) |
| C59 | C58 | C63 | 120.4(3) |  | C163 | C164 | C169 | 117.8(4) |
| C63 | C58 | N6 | 119.6(3) |  | C165 | C164 | C163 | 119.4(4) |
| C58 | C59 | C60 | 119.2(3) |  | C165 | C164 | C169 | 122.8(4) |
| C58 | C59 | C65 | 120.7(3) |  | C164 | C165 | N11 | 120.7(4) |
| C60 | C59 | C65 | 120.1(3) |  | C164 | C165 | C166 | 120.9(4) |
| C59 | C60 | C61 | 122.9(3) |  | C166 | C165 | N11 | 118.3(4) |
| C60 | C61 | C62 | 116.2(3) |  | C165 | C166 | C167 | 118.0(4) |
| C60 | C61 | C66 | 122.7(3) |  | C165 | C166 | C168 | 122.5(4) |
| C62 | C61 | C66 | 121.0(3) |  | C167 | C166 | C168 | 119.5(4) |
| C63 | C62 | C61 | 123.2(3) |  | C162 | C167 | C166 | 123.1(4) |
| C58 | C63 | C64 | 121.1(3) |  | O7 | C170 | N12 | 122.5(3) |
| C62 | C63 | C58 | 118.0(3) |  | O7 | C170 | C171 | 120.0(4) |
| C62 | C63 | C64 | 120.8(3) |  | N12 | C170 | C171 | 117.5(3) |
| C61 | C66 | C67 | 110.7(3) |  | C172 | C171 | C170 | 119.7(4) |
| C61 | C66 | C71 | 111.4(3) |  | C172 | C171 | C176 | 118.3(4) |
| C61 | C66 | C72 | 109.3(2) |  | C176 | C171 | C170 | 122.1(4) |
| C67 | C66 | C71 | 106.4(3) |  | C173 | C172 | C171 | 120.5(4) |
| C67 | C66 | C72 | 111.1(3) |  | C172 | C173 | C174 | 120.5(5) |
| C71 | C66 | C72 | 107.9(3) |  | C175 | C174 | C173 | 120.1(4) |
| C68 | C67 | C66 | 112.4(3) |  | C174 | C175 | C176 | 119.8(4) |
| C69 | C68 | C67 | 111.1(3) |  | C174 | C175 | C177 | 118.4(4) |
| C70 | C69 | C68 | 112.1(3) |  | C176 | C175 | C177 | 121.9(4) |
| C69 | C70 | C71 | 111.2(3) |  | C175 | C176 | C171 | 120.7(4) |
| C70 | C71 | C66 | 114.3(3) |  | O8 | C177 | N11 | 122.8(4) |
| C73 | C72 | C66 | 120.8(3) |  | O8 | C177 | C175 | 120.5(4) |
| C77 | C72 | C66 | 122.4(3) |  | N11 | C177 | C175 | 116.4(3) |
| C77 | C72 | C73 | 116.7(3) |  | C600 | S600 | C601 | 94.9(3) |
| C74 | C73 | C72 | 122.0(3) |  | O600 | S600 | C600 | 107.5(2) |
| C73 | C74 | C79 | 120.9(3) |  | O600 | S600 | C601 | 107.2(3) |
| C8AA | C74 | C73 | 119.2(3) |  | C200 | S200 | C201 | 97.5(4) |
| C8AA | C74 | C79 | 119.9(3) |  | O200 | S200 | C200 | 106.7(5) |
| C74 | C8AA | N12 | 120.2(3) |  | O200 | S200 | C201 | 104.6(5) |
| C74 | C8AA | C76 | 120.9(3) |  | C801 | S800 | C800 | 95.6(5) |
| C76 | C8AA | N12 | 118.8(3) |  | O800 | S800 | C801 | 104.9(4) |
| C8AA | C76 | C78 | 120.8(3) |  | O800 | S800 | C800 | 105.7(3) |
| C77 | C76 | C8AA | 117.9(3) |  | C500 | S500 | C501 | 96.8(6) |
| C77 | C76 | C78 | 121.3(4) |  | O500 | S500 | C501 | 106.9(3) |
| C76 | C77 | C72 | 123.2(3) |  | O500 | S500 | C500 | 105.4(4) |
| O6 | C80 | N10 | 122.9(3) |  | O05I | S007 | C0AA | 101.9(5) |
| O6 | C80 | C1 | 120.5(3) |  | O05I | S007 | C071 | 103.6(4) |
| N10 | C80 | C1 | 116.6(3) |  | C0AA | S007 | C071 | 100.4(5) |
| C82 | C81 | N10 | 118.0(3) |  | O300 | S300 | C301 | 104.6(3) |
| C86 | C81 | N10 | 120.9(3) |  | O300 | S300 | C300 | 106.3(4) |
| C86 | C81 | C82 | 121.1(3) |  | C300 | S300 | C301 | 98.8(3) |
| C81 | C82 | C88 | 121.2(3) |  | C1AA | S700 | C701 | 101.5(6) |
| C83 | C82 | C81 | 117.7(3) |  | O700 | S700 | C701 | 106.5(5) |
| C83 | C82 | C88 | 121.1(3) |  | O700 | S700 | C1AA | 91.9(4) |
| C84 | C83 | C82 | 122.8(3) |  | N1 | C5AA | C141 | 116.2(4) |
| C83 | C84 | C85 | 117.6(3) |  | N1 | C5AA | C143 | 123.5(4) |
| C83 | C84 | C89 | 119.5(3) |  | C143 | C5AA | C141 | 120.2(4) |
| C85 | C84 | C89 | 122.8(3) |  | O50A | S50 | C2AA | 106.4(5) |
| C84 | C85 | C86 | 121.3(3) |  | O50A | S50 | C4AA | 99.1(7) |
| C81 | C86 | C85 | 119.4(3) |  | C4AA | S50 | C2AA | 96.5(7) |
| C81 | C86 | C87 | 121.5(3) |  | C114 | C37A | C43 | 102.8(7) |
| C85 | C86 | C87 | 119.1(3) |  | C114 | C37A | C38A | 107.2(9) |
| C84 | C89 | C90 | 109.7(3) |  | C114 | C37A | C42A | 97.5(8) |
| C84 | C89 | C94 | 114.0(3) |  | C38A | C37A | C43 | 125.6(9) |
| C90 | C89 | C94 | 106.4(3) |  | C38A | C37A | C42A | 102.5(9) |
| C95 | C89 | C84 | 107.3(2) |  | C42A | C37A | C43 | 117.4(10) |
| C95 | C89 | C90 | 113.0(3) |  | C39A | C38A | C37A | 107.0(9) |
| C95 | C89 | C94 | 106.6(3) |  | C38A | C39A | C40A | 109.9(9) |
| C91 | C90 | C89 | 112.5(3) |  | C41A | C40A | C39A | 111.7(9) |
| C90 | C91 | C92 | 113.1(4) |  | C42A | C41A | C40A | 108.0(9) |
| C91 | C92 | C93 | 110.5(3) |  | C41A | C42A | C37A | 109.9(10) |
| C92 | C93 | C94 | 112.5(3) |  | C75 | S8 | C1A | 123.3(13) |
| C93 | C94 | C89 | 116.0(3) |  | O13 | S8 | C75 | 115.4(10) |
| C96 | C95 | C89 | 123.9(3) |  | O13 | S8 | C1A | 118.3(9) |

Table 6 Torsion Angles for exp\_2788.

| A | B | C | D | Angle/˚ |  | A | B | C | D | Angle/˚ |
| --- | --- | --- | --- | --- | --- | --- | --- | --- | --- | --- |
| O1 | C141 | C5AA | N1 | -173.7(5) |  | C8AA | N12 | C170 | O7 | -2.8(7) |
| O1 | C141 | C5AA | C143 | 10.6(9) |  | C8AA | N12 | C170 | C171 | 176.4(4) |
| O3 | C51 | C52 | N4 | 176.4(4) |  | C8AA | C76 | C77 | C72 | 1.5(5) |
| O3 | C51 | C52 | C53 | -2.8(6) |  | C77 | C72 | C73 | C74 | -0.7(5) |
| O7 | C170 | C171 | C172 | -19.7(7) |  | C78 | C76 | C77 | C72 | -177.7(4) |
| O7 | C170 | C171 | C176 | 159.8(4) |  | C79 | C74 | C8AA | N12 | 0.9(5) |
| O9 | C103 | C104 | C105 | -142.0(5) |  | C79 | C74 | C8AA | C76 | -176.8(3) |
| O9 | C103 | C104 | C109 | 42.1(6) |  | C80 | N10 | C81 | C82 | 108.0(4) |
| O12 | C29 | C30 | C31 | 168.0(5) |  | C80 | N10 | C81 | C86 | -68.6(5) |
| O12 | C29 | C30 | C34 | -8.3(7) |  | C80 | C1 | C2 | C3 | -178.1(4) |
| N1 | C146 | C147 | O2 | -179.1(9) |  | C81 | N10 | C80 | O6 | 3.1(6) |
| N1 | C146 | C147 | N2 | -7.6(12) |  | C81 | N10 | C80 | C1 | -175.5(3) |
| N2 | C148 | C149 | C150 | 177.3(5) |  | C81 | C82 | C83 | C84 | 0.0(5) |
| N2 | C148 | C149 | C155 | -4.1(11) |  | C82 | C81 | C86 | C85 | -1.9(5) |
| N2 | C148 | C153 | C152 | -177.0(4) |  | C82 | C81 | C86 | C87 | 178.7(3) |
| N2 | C148 | C153 | C154 | 3.4(8) |  | C82 | C83 | C84 | C85 | -2.4(5) |
| N3 | C136 | C137 | C138 | 171.3(4) |  | C82 | C83 | C84 | C89 | -178.9(3) |
| N3 | C136 | C137 | C140 | -6.2(6) |  | C83 | C84 | C85 | C86 | 2.8(5) |
| N3 | C141 | C5AA | N1 | 5.9(7) |  | C83 | C84 | C89 | C90 | -52.3(4) |
| N3 | C141 | C5AA | C143 | -169.9(5) |  | C83 | C84 | C89 | C94 | -171.5(3) |
| N4 | C52 | C53 | C54 | -1.3(6) |  | C83 | C84 | C89 | C95 | 70.8(4) |
| N4 | C56 | C57 | O4 | -177.3(3) |  | C84 | C85 | C86 | C81 | -0.7(5) |
| N4 | C56 | C57 | N6 | 4.2(4) |  | C84 | C85 | C86 | C87 | 178.8(3) |
| N5 | C46 | C47 | C48 | -171.5(3) |  | C84 | C89 | C90 | C91 | -69.9(4) |
| N5 | C46 | C47 | C49 | 8.9(5) |  | C84 | C89 | C94 | C93 | 70.1(4) |
| N5 | C51 | C52 | N4 | -5.0(5) |  | C84 | C89 | C95 | C96 | -127.1(3) |
| N5 | C51 | C52 | C53 | 175.8(3) |  | C84 | C89 | C95 | C100 | 58.0(4) |
| N6 | C58 | C59 | C60 | -179.9(3) |  | C85 | C84 | C89 | C90 | 131.5(3) |
| N6 | C58 | C59 | C65 | 1.3(5) |  | C85 | C84 | C89 | C94 | 12.3(4) |
| N6 | C58 | C63 | C62 | 180.0(3) |  | C85 | C84 | C89 | C95 | -105.5(3) |
| N6 | C58 | C63 | C64 | 1.6(4) |  | C86 | C81 | C82 | C83 | 2.2(5) |
| N7 | C24 | C25 | C26 | -178.1(4) |  | C86 | C81 | C82 | C88 | -177.7(3) |
| N7 | C24 | C25 | C27 | 2.7(6) |  | C88 | C82 | C83 | C84 | 180.0(3) |
| N7 | C29 | C30 | C31 | -8.2(7) |  | C89 | C84 | C85 | C86 | 179.1(3) |
| N7 | C29 | C30 | C34 | 175.5(4) |  | C89 | C90 | C91 | C92 | -59.6(4) |
| N8 | C7 | C8 | C9 | -179.4(3) |  | C89 | C95 | C96 | C97 | -174.4(3) |
| N8 | C7 | C8 | C14 | 0.5(5) |  | C89 | C95 | C100 | C99 | 174.8(3) |
| N8 | C7 | C12 | C11 | 180.0(3) |  | C90 | C89 | C94 | C93 | -50.9(4) |
| N8 | C7 | C12 | C13 | 1.3(6) |  | C90 | C89 | C95 | C96 | -6.1(5) |
| N9 | C1 | C2 | C3 | 0.6(6) |  | C90 | C89 | C95 | C100 | 179.0(3) |
| N9 | C1 | C80 | O6 | 174.7(4) |  | C90 | C91 | C92 | C93 | 55.6(5) |
| N9 | C1 | C80 | N10 | -6.6(5) |  | C91 | C92 | C93 | C94 | -50.3(5) |
| N9 | C5 | C6 | O5 | -175.0(4) |  | C92 | C93 | C94 | C89 | 51.2(4) |
| N9 | C5 | C6 | N8 | 5.8(5) |  | C94 | C89 | C90 | C91 | 53.9(4) |
| N10 | C81 | C82 | C83 | -174.4(3) |  | C94 | C89 | C95 | C96 | 110.4(4) |
| N10 | C81 | C82 | C88 | 5.7(5) |  | C94 | C89 | C95 | C100 | -64.5(4) |
| N10 | C81 | C86 | C85 | 174.6(3) |  | C95 | C89 | C90 | C91 | 170.5(3) |
| N10 | C81 | C86 | C87 | -4.8(5) |  | C95 | C89 | C94 | C93 | -171.7(3) |
| N11 | C165 | C166 | C167 | 179.9(4) |  | C95 | C96 | C97 | C98 | -0.4(6) |
| N11 | C165 | C166 | C168 | -0.1(7) |  | C95 | C96 | C97 | C101 | 178.0(4) |
| N12 | C8AA | C76 | C77 | 179.6(3) |  | C96 | C95 | C100 | C99 | -0.4(5) |
| N12 | C8AA | C76 | C78 | -1.1(5) |  | C96 | C97 | C98 | N13 | 173.8(3) |
| N12 | C170 | C171 | C172 | 161.0(5) |  | C96 | C97 | C98 | C99 | -0.1(5) |
| N12 | C170 | C171 | C176 | -19.4(6) |  | C97 | C98 | C99 | C100 | 0.3(5) |
| N13 | C98 | C99 | C100 | -173.7(3) |  | C97 | C98 | C99 | C102 | 177.4(3) |
| N13 | C98 | C99 | C102 | 3.5(5) |  | C98 | N13 | C103 | O9 | -10.1(7) |
| N13 | C103 | C104 | C105 | 41.8(6) |  | C98 | N13 | C103 | C104 | 166.2(4) |
| N13 | C103 | C104 | C109 | -134.1(4) |  | C98 | C99 | C100 | C95 | 0.0(5) |
| N14 | C111 | C112 | C113 | 177.8(3) |  | C100 | C95 | C96 | C97 | 0.6(5) |
| N14 | C111 | C112 | C118 | -1.8(5) |  | C101 | C97 | C98 | N13 | -4.5(5) |
| N14 | C111 | C116 | C115 | -177.0(3) |  | C101 | C97 | C98 | C99 | -178.4(4) |
| N14 | C111 | C116 | C117 | 4.5(6) |  | C102 | C99 | C100 | C95 | -177.2(3) |
| N15 | C119 | C120 | C121 | -175.5(3) |  | C103 | N13 | C98 | C97 | 112.0(4) |
| N15 | C119 | C120 | C126 | 1.5(5) |  | C103 | N13 | C98 | C99 | -74.0(5) |
| N15 | C119 | C124 | C123 | 174.9(3) |  | C103 | C104 | C105 | C106 | -177.3(4) |
| N15 | C119 | C124 | C125 | -5.4(5) |  | C103 | C104 | C109 | C108 | 177.1(3) |
| C1 | N9 | C5 | C4 | -0.9(6) |  | C104 | C105 | C106 | C107 | 0.6(6) |
| C1 | N9 | C5 | C6 | 175.8(3) |  | C105 | C104 | C109 | C108 | 1.1(6) |
| C1 | C2 | C3 | C4 | -1.6(6) |  | C105 | C106 | C107 | C108 | 0.5(6) |
| C2 | C1 | C80 | O6 | -6.5(6) |  | C106 | C107 | C108 | C109 | -0.9(6) |
| C2 | C1 | C80 | N10 | 172.1(4) |  | C106 | C107 | C108 | C110 | 175.4(4) |
| C2 | C3 | C4 | C5 | 1.3(6) |  | C107 | C108 | C109 | C104 | 0.1(5) |
| C3 | C4 | C5 | N9 | 0.0(6) |  | C107 | C108 | C110 | O10 | -150.4(4) |
| C3 | C4 | C5 | C6 | -176.6(4) |  | C107 | C108 | C110 | N14 | 28.7(5) |
| C4 | C5 | C6 | O5 | 1.8(6) |  | C109 | C104 | C105 | C106 | -1.4(6) |
| C4 | C5 | C6 | N8 | -177.4(4) |  | C109 | C108 | C110 | O10 | 25.9(6) |
| C5 | N9 | C1 | C2 | 0.6(5) |  | C109 | C108 | C110 | N14 | -155.0(4) |
| C5 | N9 | C1 | C80 | 179.3(3) |  | C110 | N14 | C111 | C112 | 81.1(5) |
| C6 | N8 | C7 | C8 | 101.7(4) |  | C110 | N14 | C111 | C116 | -101.4(4) |
| C6 | N8 | C7 | C12 | -80.0(5) |  | C110 | C108 | C109 | C104 | -176.3(3) |
| C7 | N8 | C6 | O5 | 7.8(7) |  | C111 | N14 | C110 | O10 | 7.8(6) |
| C7 | N8 | C6 | C5 | -173.1(4) |  | C111 | N14 | C110 | C108 | -171.2(4) |
| C7 | C8 | C9 | C10 | -1.2(5) |  | C111 | C112 | C113 | C114 | -0.9(6) |
| C8 | C7 | C12 | C11 | -1.8(6) |  | C112 | C111 | C116 | C115 | 0.5(6) |
| C8 | C7 | C12 | C13 | 179.6(4) |  | C112 | C111 | C116 | C117 | -178.0(4) |
| C8 | C9 | C10 | C11 | -0.4(5) |  | C112 | C113 | C114 | C37 | 179.0(5) |
| C8 | C9 | C10 | C15 | 179.6(3) |  | C112 | C113 | C114 | C115 | 0.6(5) |
| C9 | C10 | C11 | C12 | 1.0(6) |  | C112 | C113 | C114 | C37A | 176.1(6) |
| C9 | C10 | C15 | C16 | -65.4(4) |  | C113 | C114 | C115 | C116 | 0.3(6) |
| C9 | C10 | C15 | C20 | 174.8(3) |  | C113 | C114 | C37A | C43 | -53.1(9) |
| C9 | C10 | C15 | C21 | 55.9(4) |  | C113 | C114 | C37A | C38A | 172.9(7) |
| C10 | C11 | C12 | C7 | 0.1(6) |  | C113 | C114 | C37A | C42A | 67.3(9) |
| C10 | C11 | C12 | C13 | 178.8(4) |  | C114 | C37 | C38 | C39 | -76.4(9) |
| C10 | C15 | C16 | C17 | -167.6(4) |  | C114 | C37 | C42 | C41 | 74.3(8) |
| C10 | C15 | C20 | C19 | 170.2(4) |  | C114 | C37 | C43 | C44 | 102.6(6) |
| C10 | C15 | C21 | C22 | -112.7(4) |  | C114 | C37 | C43 | C48 | -68.2(7) |
| C10 | C15 | C21 | C26 | 63.5(4) |  | C114 | C115 | C116 | C111 | -0.8(6) |
| C11 | C10 | C15 | C16 | 114.6(4) |  | C114 | C115 | C116 | C117 | 177.7(4) |
| C11 | C10 | C15 | C20 | -5.2(5) |  | C114 | C37A | C38A | C39A | -169.2(9) |
| C11 | C10 | C15 | C21 | -124.1(4) |  | C114 | C37A | C42A | C41A | 178.9(9) |
| C12 | C7 | C8 | C9 | 2.3(5) |  | C115 | C114 | C37A | C43 | 122.3(7) |
| C12 | C7 | C8 | C14 | -177.8(3) |  | C115 | C114 | C37A | C38A | -11.7(10) |
| C14 | C8 | C9 | C10 | 178.9(3) |  | C115 | C114 | C37A | C42A | -117.3(7) |
| C15 | C10 | C11 | C12 | -179.0(4) |  | C116 | C111 | C112 | C113 | 0.3(6) |
| C15 | C16 | C17 | C18 | 50.0(7) |  | C116 | C111 | C112 | C118 | -179.3(4) |
| C15 | C21 | C22 | C23 | 177.9(4) |  | C118 | C112 | C113 | C114 | 178.7(4) |
| C15 | C21 | C26 | C25 | -177.1(4) |  | C119 | N15 | C36 | O11 | 4.7(5) |
| C16 | C15 | C20 | C19 | 50.1(6) |  | C119 | N15 | C36 | C35 | -175.6(3) |
| C16 | C15 | C21 | C22 | 5.7(5) |  | C119 | C120 | C121 | C122 | 1.8(5) |
| C16 | C15 | C21 | C26 | -178.1(3) |  | C120 | C119 | C124 | C123 | -3.0(5) |
| C16 | C17 | C18 | C19 | -54.2(8) |  | C120 | C119 | C124 | C125 | 176.7(3) |
| C17 | C18 | C19 | C20 | 57.5(8) |  | C120 | C121 | C122 | C123 | -5.2(5) |
| C18 | C19 | C20 | C15 | -58.7(7) |  | C120 | C121 | C122 | C127 | 171.8(3) |
| C20 | C15 | C16 | C17 | -44.3(6) |  | C121 | C122 | C123 | C124 | 4.6(5) |
| C20 | C15 | C21 | C22 | 125.1(4) |  | C121 | C122 | C127 | C128 | -62.3(4) |
| C20 | C15 | C21 | C26 | -58.8(4) |  | C121 | C122 | C127 | C132 | 179.7(4) |
| C21 | C15 | C16 | C17 | 75.4(5) |  | C121 | C122 | C127 | C133 | 61.2(5) |
| C21 | C15 | C20 | C19 | -72.2(5) |  | C122 | C123 | C124 | C119 | -0.6(5) |
| C21 | C22 | C23 | C24 | -0.6(6) |  | C122 | C123 | C124 | C125 | 179.7(3) |
| C21 | C22 | C23 | C28 | -178.5(5) |  | C122 | C127 | C128 | C129 | -173.5(4) |
| C22 | C21 | C26 | C25 | -0.7(6) |  | C122 | C127 | C132 | C131 | 174.1(4) |
| C22 | C23 | C24 | N7 | 179.1(4) |  | C122 | C127 | C133 | C134 | -92.2(5) |
| C22 | C23 | C24 | C25 | -1.6(6) |  | C122 | C127 | C133 | C138 | 83.3(5) |
| C23 | C24 | C25 | C26 | 2.6(6) |  | C123 | C122 | C127 | C128 | 114.5(5) |
| C23 | C24 | C25 | C27 | -176.6(5) |  | C123 | C122 | C127 | C132 | -3.5(5) |
| C24 | N7 | C29 | O12 | -4.2(8) |  | C123 | C122 | C127 | C133 | -121.9(4) |
| C24 | N7 | C29 | C30 | 171.9(4) |  | C124 | C119 | C120 | C121 | 2.4(5) |
| C24 | C25 | C26 | C21 | -1.4(6) |  | C124 | C119 | C120 | C126 | 179.5(3) |
| C26 | C21 | C22 | C23 | 1.7(6) |  | C126 | C120 | C121 | C122 | -175.2(3) |
| C27 | C25 | C26 | C21 | 177.8(5) |  | C127 | C122 | C123 | C124 | -172.3(3) |
| C28 | C23 | C24 | N7 | -3.0(7) |  | C127 | C128 | C129 | C130 | 53.0(5) |
| C28 | C23 | C24 | C25 | 176.3(5) |  | C127 | C133 | C134 | C135 | 174.1(3) |
| C29 | N7 | C24 | C23 | 75.1(6) |  | C127 | C133 | C138 | C137 | -174.1(4) |
| C29 | N7 | C24 | C25 | -104.2(5) |  | C128 | C127 | C132 | C131 | 56.4(5) |
| C29 | C30 | C31 | C32 | -174.7(4) |  | C128 | C127 | C133 | C134 | 27.4(5) |
| C29 | C30 | C34 | C35 | 172.8(4) |  | C128 | C127 | C133 | C138 | -157.1(4) |
| C30 | C31 | C32 | C33 | 1.9(7) |  | C128 | C129 | C130 | C131 | -52.5(5) |
| C30 | C34 | C35 | C33 | 2.4(6) |  | C129 | C130 | C131 | C132 | 57.5(6) |
| C30 | C34 | C35 | C36 | -178.5(3) |  | C130 | C131 | C132 | C127 | -60.4(6) |
| C31 | C30 | C34 | C35 | -3.7(6) |  | C132 | C127 | C128 | C129 | -54.3(5) |
| C31 | C32 | C33 | C35 | -3.2(7) |  | C132 | C127 | C133 | C134 | 148.3(4) |
| C32 | C33 | C35 | C34 | 1.0(6) |  | C132 | C127 | C133 | C138 | -36.2(5) |
| C32 | C33 | C35 | C36 | -178.0(4) |  | C133 | C127 | C128 | C129 | 66.8(6) |
| C33 | C35 | C36 | O11 | 129.2(4) |  | C133 | C127 | C132 | C131 | -68.0(5) |
| C33 | C35 | C36 | N15 | -50.5(5) |  | C133 | C134 | C135 | C136 | -1.0(6) |
| C34 | C30 | C31 | C32 | 1.6(7) |  | C133 | C134 | C135 | C139 | -177.8(4) |
| C34 | C35 | C36 | O11 | -49.8(5) |  | C134 | C133 | C138 | C137 | 1.5(6) |
| C34 | C35 | C36 | N15 | 130.5(3) |  | C134 | C135 | C136 | N3 | -171.3(3) |
| C36 | N15 | C119 | C120 | 112.9(4) |  | C134 | C135 | C136 | C137 | 4.0(6) |
| C36 | N15 | C119 | C124 | -65.0(4) |  | C135 | C136 | C137 | C138 | -4.1(6) |
| C37 | C38 | C39 | C40 | -55.8(9) |  | C135 | C136 | C137 | C140 | 178.4(4) |
| C37 | C43 | C44 | C45 | -168.4(5) |  | C136 | N3 | C141 | O1 | -8.3(9) |
| C37 | C43 | C48 | C47 | 170.2(4) |  | C136 | N3 | C141 | C5AA | 172.1(5) |
| C37 | C114 | C115 | C116 | -177.9(5) |  | C136 | C137 | C138 | C133 | 1.3(6) |
| C38 | C37 | C42 | C41 | -60.5(9) |  | C138 | C133 | C134 | C135 | -1.7(6) |
| C38 | C37 | C43 | C44 | -133.9(6) |  | C139 | C135 | C136 | N3 | 5.4(6) |
| C38 | C37 | C43 | C48 | 55.3(7) |  | C139 | C135 | C136 | C137 | -179.3(4) |
| C38 | C37 | C114 | C113 | -176.6(5) |  | C140 | C137 | C138 | C133 | 178.8(4) |
| C38 | C37 | C114 | C115 | 1.5(9) |  | C141 | N3 | C136 | C135 | -66.5(6) |
| C38 | C39 | C40 | C41 | 54.6(8) |  | C141 | N3 | C136 | C137 | 118.1(5) |
| C39 | C40 | C41 | C42 | -56.2(8) |  | C143 | C144 | C145 | C146 | 3.3(13) |
| C40 | C41 | C42 | C37 | 60.1(9) |  | C144 | C143 | C5AA | N1 | 4.8(10) |
| C42 | C37 | C38 | C39 | 57.1(9) |  | C144 | C143 | C5AA | C141 | -179.7(6) |
| C42 | C37 | C43 | C44 | -20.7(7) |  | C144 | C145 | C146 | N1 | -1.1(13) |
| C42 | C37 | C43 | C48 | 168.5(5) |  | C144 | C145 | C146 | C147 | -178.4(8) |
| C42 | C37 | C114 | C113 | 53.2(7) |  | C145 | C146 | C147 | O2 | -1.6(14) |
| C42 | C37 | C114 | C115 | -128.6(6) |  | C145 | C146 | C147 | N2 | 169.9(8) |
| C43 | C37 | C38 | C39 | 169.5(7) |  | C146 | N1 | C5AA | C141 | -178.2(6) |
| C43 | C37 | C42 | C41 | -169.2(6) |  | C146 | N1 | C5AA | C143 | -2.6(9) |
| C43 | C37 | C114 | C113 | -64.4(7) |  | C147 | N2 | C148 | C149 | -109.9(10) |
| C43 | C37 | C114 | C115 | 113.8(6) |  | C147 | N2 | C148 | C153 | 76.4(9) |
| C43 | C44 | C45 | C46 | -0.4(6) |  | C148 | N2 | C147 | O2 | 9.1(15) |
| C43 | C44 | C45 | C50 | 177.1(4) |  | C148 | N2 | C147 | C146 | -162.3(8) |
| C43 | C37A | C38A | C39A | 70.3(13) |  | C148 | C149 | C150 | C151 | 4.8(11) |
| C43 | C37A | C42A | C41A | -72.4(11) |  | C149 | C148 | C153 | C152 | 8.8(8) |
| C44 | C43 | C48 | C47 | -0.9(5) |  | C149 | C148 | C153 | C154 | -170.8(6) |
| C44 | C43 | C37A | C114 | 93.7(8) |  | C149 | C150 | C151 | C152 | 0.2(8) |
| C44 | C43 | C37A | C38A | -144.0(9) |  | C149 | C150 | C151 | C156 | 179.9(5) |
| C44 | C43 | C37A | C42A | -12.0(12) |  | C150 | C151 | C152 | C153 | -0.2(7) |
| C44 | C45 | C46 | N5 | 172.8(3) |  | C150 | C151 | C156 | C157 | 104.5(5) |
| C44 | C45 | C46 | C47 | -3.5(5) |  | C150 | C151 | C156 | C161 | -10.7(6) |
| C45 | C46 | C47 | C48 | 5.0(5) |  | C150 | C151 | C156 | C162 | -134.8(5) |
| C45 | C46 | C47 | C49 | -174.6(3) |  | C151 | C152 | C153 | C148 | -4.4(8) |
| C46 | N5 | C51 | O3 | -6.3(7) |  | C151 | C152 | C153 | C154 | 175.2(5) |
| C46 | N5 | C51 | C52 | 175.2(4) |  | C151 | C156 | C157 | C158 | -175.6(4) |
| C46 | C47 | C48 | C43 | -2.8(5) |  | C151 | C156 | C161 | C160 | 173.5(3) |
| C48 | C43 | C44 | C45 | 2.6(6) |  | C151 | C156 | C162 | C163 | 93.6(4) |
| C48 | C43 | C37A | C114 | -79.3(8) |  | C151 | C156 | C162 | C167 | -82.0(5) |
| C48 | C43 | C37A | C38A | 43.0(12) |  | C152 | C151 | C156 | C157 | -75.7(4) |
| C48 | C43 | C37A | C42A | 175.0(7) |  | C152 | C151 | C156 | C161 | 169.1(4) |
| C49 | C47 | C48 | C43 | 176.8(3) |  | C152 | C151 | C156 | C162 | 44.9(5) |
| C50 | C45 | C46 | N5 | -4.6(6) |  | C153 | C148 | C149 | C150 | -9.0(10) |
| C50 | C45 | C46 | C47 | 179.0(3) |  | C153 | C148 | C149 | C155 | 169.7(7) |
| C51 | N5 | C46 | C45 | 67.6(5) |  | C156 | C151 | C152 | C153 | -180.0(4) |
| C51 | N5 | C46 | C47 | -116.0(4) |  | C156 | C157 | C158 | C159 | 56.6(5) |
| C51 | C52 | C53 | C54 | 177.9(3) |  | C156 | C162 | C163 | C164 | -175.5(3) |
| C52 | N4 | C56 | C55 | -0.4(5) |  | C156 | C162 | C167 | C166 | 173.7(4) |
| C52 | N4 | C56 | C57 | -179.0(3) |  | C157 | C156 | C161 | C160 | 58.1(4) |
| C52 | C53 | C54 | C55 | 2.0(5) |  | C157 | C156 | C162 | C163 | -148.7(3) |
| C53 | C54 | C55 | C56 | -1.9(5) |  | C157 | C156 | C162 | C167 | 35.7(5) |
| C54 | C55 | C56 | N4 | 1.1(5) |  | C157 | C158 | C159 | C160 | -53.5(5) |
| C54 | C55 | C56 | C57 | 179.6(3) |  | C158 | C159 | C160 | C161 | 53.8(5) |
| C55 | C56 | C57 | O4 | 4.1(5) |  | C159 | C160 | C161 | C156 | -58.4(4) |
| C55 | C56 | C57 | N6 | -174.4(3) |  | C161 | C156 | C157 | C158 | -57.5(4) |
| C56 | N4 | C52 | C51 | -178.7(3) |  | C161 | C156 | C162 | C163 | -29.6(5) |
| C56 | N4 | C52 | C53 | 0.5(5) |  | C161 | C156 | C162 | C167 | 154.8(4) |
| C57 | N6 | C58 | C59 | 69.0(4) |  | C162 | C156 | C157 | C158 | 65.1(4) |
| C57 | N6 | C58 | C63 | -111.3(3) |  | C162 | C156 | C161 | C160 | -64.0(4) |
| C58 | N6 | C57 | O4 | 8.8(5) |  | C162 | C163 | C164 | C165 | 0.9(5) |
| C58 | N6 | C57 | C56 | -172.8(3) |  | C162 | C163 | C164 | C169 | -179.6(3) |
| C58 | C59 | C60 | C61 | 1.3(5) |  | C163 | C162 | C167 | C166 | -2.2(6) |
| C59 | C58 | C63 | C62 | -0.3(5) |  | C163 | C164 | C165 | N11 | 178.4(3) |
| C59 | C58 | C63 | C64 | -178.6(3) |  | C163 | C164 | C165 | C166 | -0.4(6) |
| C59 | C60 | C61 | C62 | -2.9(5) |  | C164 | C165 | C166 | C167 | -1.3(6) |
| C59 | C60 | C61 | C66 | -178.3(3) |  | C164 | C165 | C166 | C168 | 178.7(5) |
| C60 | C61 | C62 | C63 | 3.0(5) |  | C165 | N11 | C177 | O8 | -5.2(9) |
| C60 | C61 | C66 | C67 | -142.5(3) |  | C165 | N11 | C177 | C175 | -179.3(5) |
| C60 | C61 | C66 | C71 | -24.3(4) |  | C165 | C166 | C167 | C162 | 2.7(7) |
| C60 | C61 | C66 | C72 | 94.8(3) |  | C167 | C162 | C163 | C164 | 0.3(5) |
| C61 | C62 | C63 | C58 | -1.5(5) |  | C168 | C166 | C167 | C162 | -177.4(5) |
| C61 | C62 | C63 | C64 | 176.9(3) |  | C169 | C164 | C165 | N11 | -1.1(6) |
| C61 | C66 | C67 | C68 | 63.5(4) |  | C169 | C164 | C165 | C166 | -179.8(4) |
| C61 | C66 | C71 | C70 | -63.8(4) |  | C170 | N12 | C8AA | C74 | 96.2(4) |
| C61 | C66 | C72 | C73 | 165.0(3) |  | C170 | N12 | C8AA | C76 | -86.0(5) |
| C61 | C66 | C72 | C77 | -18.8(4) |  | C170 | C171 | C172 | C173 | 178.2(6) |
| C62 | C61 | C66 | C67 | 42.4(4) |  | C170 | C171 | C176 | C175 | -177.4(4) |
| C62 | C61 | C66 | C71 | 160.6(3) |  | C171 | C172 | C173 | C174 | 0.4(11) |
| C62 | C61 | C66 | C72 | -80.3(4) |  | C172 | C171 | C176 | C175 | 2.2(7) |
| C63 | C58 | C59 | C60 | 0.3(5) |  | C172 | C173 | C174 | C175 | -0.2(11) |
| C63 | C58 | C59 | C65 | -178.4(3) |  | C173 | C174 | C175 | C176 | 1.0(9) |
| C65 | C59 | C60 | C61 | -179.9(3) |  | C173 | C174 | C175 | C177 | -179.0(6) |
| C66 | C61 | C62 | C63 | 178.4(3) |  | C174 | C175 | C176 | C171 | -2.0(7) |
| C66 | C67 | C68 | C69 | 57.8(4) |  | C174 | C175 | C177 | O8 | 38.4(8) |
| C66 | C72 | C73 | C74 | 175.7(3) |  | C174 | C175 | C177 | N11 | -147.3(5) |
| C66 | C72 | C77 | C76 | -176.2(3) |  | C176 | C171 | C172 | C173 | -1.4(9) |
| C67 | C66 | C71 | C70 | 56.9(4) |  | C176 | C175 | C177 | O8 | -141.6(5) |
| C67 | C66 | C72 | C73 | 42.5(4) |  | C176 | C175 | C177 | N11 | 32.6(7) |
| C67 | C66 | C72 | C77 | -141.3(3) |  | C177 | N11 | C165 | C164 | 68.5(7) |
| C67 | C68 | C69 | C70 | -53.1(4) |  | C177 | N11 | C165 | C166 | -112.7(5) |
| C68 | C69 | C70 | C71 | 51.3(4) |  | C177 | C175 | C176 | C171 | 178.0(4) |
| C69 | C70 | C71 | C66 | -55.1(4) |  | C155 | C149 | C150 | C151 | -173.9(8) |
| C71 | C66 | C67 | C68 | -57.7(4) |  | C5AA | N1 | C146 | C145 | 0.7(11) |
| C71 | C66 | C72 | C73 | -73.7(4) |  | C5AA | N1 | C146 | C147 | 178.0(7) |
| C71 | C66 | C72 | C77 | 102.5(4) |  | C5AA | C143 | C144 | C145 | -5.1(12) |
| C72 | C66 | C67 | C68 | -174.9(3) |  | C37A | C43 | C44 | C45 | -170.1(6) |
| C72 | C66 | C71 | C70 | 176.2(3) |  | C37A | C43 | C48 | C47 | 172.9(5) |
| C72 | C73 | C74 | C8AA | -0.3(5) |  | C37A | C114 | C115 | C116 | -175.5(6) |
| C72 | C73 | C74 | C79 | 178.5(3) |  | C37A | C38A | C39A | C40A | 63.1(12) |
| C73 | C72 | C77 | C76 | 0.1(5) |  | C38A | C37A | C42A | C41A | 69.4(12) |
| C73 | C74 | C8AA | N12 | 179.7(3) |  | C38A | C39A | C40A | C41A | -56.6(13) |
| C73 | C74 | C8AA | C76 | 2.1(5) |  | C39A | C40A | C41A | C42A | 55.6(13) |
| C74 | C8AA | C76 | C77 | -2.7(5) |  | C40A | C41A | C42A | C37A | -63.7(12) |
| C74 | C8AA | C76 | C78 | 176.6(4) |  | C42A | C37A | C38A | C39A | -67.2(12) |

Table 7 Hydrogen Atom Coordinates (Å×104) and Isotropic Displacement Parameters (Å2×103) for exp\_2788.

| Atom | *x* | *y* | *z* | U(eq) |
| --- | --- | --- | --- | --- |
| H2 | 9741.21 | -1887.63 | 7887.38 | 172 |
| H3 | 9334.07 | -1588.13 | 6866.75 | 99 |
| H5 | 2294.63 | 984.15 | 6393.24 | 77 |
| H6 | 3038.7 | 1828.78 | 5897.84 | 56 |
| H7 | 1904.56 | 3237.44 | 7989.14 | 84 |
| H8 | 4647.06 | 5898.85 | 6567.82 | 72 |
| H10 | 6252.19 | 5155.27 | 6295.73 | 65 |
| H11 | 7308.62 | 1315.54 | 9176.65 | 95 |
| H12 | 6288.51 | 2447.24 | 8153.7 | 66 |
| H13 | 8538.14 | 866.01 | 7777.88 | 70 |
| H14 | 5683.44 | -318.13 | 8866.1 | 80 |
| H15 | 4619.89 | 761.2 | 7398.6 | 59 |
| H2A | 6758.67 | 6568.02 | 5302.83 | 67 |
| H3A | 5823.25 | 7466.15 | 5275.54 | 75 |
| H4 | 4677.44 | 7529.79 | 5701.8 | 74 |
| H9 | 2181.97 | 5584.95 | 6634.74 | 62 |
| H11A | 2457.77 | 6358.21 | 7690.8 | 78 |
| H13A | 3689.23 | 6987.45 | 7211.09 | 137 |
| H13B | 4281.48 | 6380.81 | 7447.98 | 137 |
| H13C | 3528.08 | 6665.79 | 7728.88 | 137 |
| H14A | 3932.35 | 5331.36 | 6155.2 | 94 |
| H14B | 3434.73 | 5930.33 | 5841.66 | 94 |
| H14C | 3068.66 | 5427.53 | 6005.95 | 94 |
| H16A | 815.55 | 6623.81 | 6958.87 | 86 |
| H16B | 816.55 | 6061.17 | 6783.92 | 86 |
| H17A | -116.56 | 5932.85 | 7305.78 | 133 |
| H17B | -402.52 | 6587.3 | 7035.87 | 133 |
| H18A | -656.19 | 6489.06 | 7866.73 | 162 |
| H18B | -123.54 | 6885.15 | 7704.26 | 162 |
| H19A | 418.87 | 6200.59 | 8407.21 | 132 |
| H19B | 439.57 | 5686.63 | 8151.03 | 132 |
| H20A | 1643.53 | 5791.22 | 8145.39 | 89 |
| H20B | 1302.09 | 6443.7 | 7878.88 | 89 |
| H22 | 790.14 | 5233.27 | 7018.37 | 83 |
| H26 | 2427.71 | 5000.24 | 7982.53 | 79 |
| H27A | 3384.07 | 3632.77 | 8041.89 | 174 |
| H27B | 2787.55 | 3607.62 | 8466.26 | 174 |
| H27C | 3183.34 | 4097.78 | 8375.72 | 174 |
| H28A | 1233.24 | 3839.68 | 6889.73 | 162 |
| H28B | 621.28 | 4466.03 | 6746.22 | 162 |
| H28C | 496.24 | 4033.61 | 7212.93 | 162 |
| H31 | 2362.29 | 2367.45 | 8265.49 | 81 |
| H32 | 2834.07 | 1373.88 | 8462.29 | 87 |
| H33 | 3507.8 | 840.64 | 7907.73 | 73 |
| H34 | 3074.24 | 2300.16 | 6902.34 | 60 |
| H38A | 1706.48 | 204.18 | 8706.71 | 76 |
| H38B | 1290.31 | -201.45 | 8549.82 | 76 |
| H39A | 2265.21 | -498.19 | 9373.19 | 86 |
| H39B | 1358.84 | -378.51 | 9386.52 | 86 |
| H40A | 1496.84 | -1217.79 | 9116.13 | 88 |
| H40B | 2045 | -1374.69 | 9573.8 | 88 |
| H41A | 3120.43 | -1413.97 | 9082.39 | 81 |
| H41B | 2705.59 | -1833.85 | 8949.94 | 81 |
| H42A | 2082.62 | -1122.52 | 8291.47 | 73 |
| H42B | 2994.03 | -1275.49 | 8243.99 | 73 |
| H44 | 2468.2 | -817.41 | 7585.73 | 84 |
| H48 | 1865.7 | 688.13 | 8038.06 | 80 |
| H49A | 1024.82 | 1562.13 | 7038.11 | 109 |
| H49B | 1881.43 | 1588.42 | 7009.64 | 109 |
| H49C | 1443.77 | 1577.66 | 7507.79 | 109 |
| H50A | 1902.78 | -458.49 | 6502.55 | 120 |
| H50B | 2697.49 | -863.78 | 6756.88 | 120 |
| H50C | 2667.31 | -291.53 | 6380.87 | 120 |
| H53 | 744.39 | 1107.99 | 5258.18 | 75 |
| H54 | 817.75 | 1797.56 | 4575.75 | 71 |
| H55 | 1678.72 | 2304.07 | 4581.21 | 64 |
| H60 | 3491.8 | 3667.38 | 6008.19 | 55 |
| H62 | 5358.67 | 2326.68 | 5852.13 | 54 |
| H64A | 4684.28 | 1606.43 | 5310.83 | 83 |
| H64B | 4439.27 | 1334.74 | 5825.46 | 83 |
| H64C | 5254.67 | 1435.23 | 5758.48 | 83 |
| H65A | 2142.67 | 3387.9 | 5552.79 | 88 |
| H65B | 2240.47 | 3607.21 | 6024.83 | 88 |
| H65C | 2134.75 | 2993.59 | 6066.58 | 88 |
| H67A | 6114.53 | 3473.48 | 5790.19 | 61 |
| H67B | 6035.44 | 2872.37 | 5743.58 | 61 |
| H68A | 5998.28 | 3461.34 | 4968.09 | 70 |
| H68B | 5207.91 | 3324.34 | 5064.94 | 70 |
| H69A | 5435.25 | 4387.66 | 5128.11 | 74 |
| H69B | 4901.07 | 4310.74 | 4741.65 | 74 |
| H70A | 3999.82 | 4172.97 | 5318.17 | 66 |
| H70B | 4172.69 | 4740.25 | 5374.33 | 66 |
| H71A | 4176.1 | 4131.72 | 6136.25 | 59 |
| H71B | 4983.52 | 4237.79 | 6036.72 | 59 |
| H73 | 6220.44 | 3500.75 | 6510.02 | 56 |
| H77 | 4486.01 | 2832.09 | 6812.07 | 63 |
| H78A | 4320.55 | 2336.02 | 7584.07 | 115 |
| H78B | 4371.34 | 2708.73 | 7964.14 | 115 |
| H78C | 5034.73 | 2116.94 | 7946.93 | 115 |
| H79A | 7043.5 | 3508.47 | 7114.23 | 84 |
| H79B | 7218.35 | 2946.13 | 7525.65 | 84 |
| H79C | 6664.52 | 3546.51 | 7627.54 | 84 |
| H83 | 8592.92 | 3722.91 | 7003.81 | 58 |
| H85 | 8159.3 | 3458.18 | 5716.02 | 56 |
| H87A | 7162.84 | 4656.67 | 5183.2 | 92 |
| H87B | 6428.6 | 4579 | 5476.5 | 92 |
| H87C | 7015.78 | 4051.89 | 5279.41 | 92 |
| H88A | 6976.53 | 4745.52 | 7153.92 | 103 |
| H88B | 7521.37 | 5102.4 | 6910.41 | 103 |
| H88C | 7861.85 | 4534.53 | 7307.22 | 103 |
| H90A | 9768.1 | 3262.68 | 6890.32 | 67 |
| H90B | 10299.24 | 2676.01 | 6751.12 | 67 |
| H91A | 10693.33 | 3444.98 | 6346.1 | 81 |
| H91B | 9857.42 | 3761.96 | 6111.78 | 81 |
| H92A | 10911.13 | 2722.99 | 5905.05 | 82 |
| H92B | 10667.34 | 3331.22 | 5545.07 | 82 |
| H93A | 10000.1 | 2704.51 | 5383.83 | 75 |
| H93B | 9432.5 | 3311.38 | 5462.97 | 75 |
| H94A | 9767.84 | 2252.29 | 6134.34 | 64 |
| H94B | 8953.75 | 2616.28 | 5888.76 | 64 |
| H96 | 9757.77 | 2297.43 | 7349.05 | 64 |
| H100 | 7968.59 | 2585.33 | 6468.13 | 57 |
| H10A | 9958.45 | 1643.64 | 8085.84 | 122 |
| H10B | 9816.33 | 1070.8 | 8016.32 | 122 |
| H10C | 9230.4 | 1485.1 | 8321.83 | 122 |
| H10D | 6908.06 | 1937.26 | 7160.86 | 93 |
| H10E | 7460.86 | 1423.01 | 6932.66 | 93 |
| H10F | 7128.47 | 2049.79 | 6609.43 | 93 |
| H105 | 8513.87 | 186.01 | 8427.01 | 75 |
| H106 | 8162.31 | -642.15 | 8688.25 | 82 |
| H107 | 6874.92 | -591.36 | 8663.79 | 78 |
| H109 | 6272.17 | 1110.3 | 8116.06 | 71 |
| H113 | 3793.2 | -770.46 | 7988.08 | 73 |
| H115 | 2926.35 | 119.34 | 9026.61 | 86 |
| H11B | 4222.94 | 771.46 | 9031.8 | 132 |
| H11C | 4567.76 | 202.68 | 9426.96 | 132 |
| H11D | 3664.81 | 533.45 | 9400.75 | 132 |
| H11E | 5054.68 | -951.7 | 7738.82 | 109 |
| H11F | 5655.7 | -992.64 | 8138 | 109 |
| H11G | 5369.45 | -428.33 | 7730.73 | 109 |
| H121 | 6573.08 | 349.54 | 6215.7 | 64 |
| H123 | 5066.85 | -491.66 | 6158.59 | 71 |
| H12A | 3590.68 | 170.64 | 6573.33 | 96 |
| H12B | 3910.51 | 0.14 | 7110.46 | 96 |
| H12C | 4097.51 | -475 | 6786.88 | 96 |
| H12D | 5946.81 | 924.92 | 7175.1 | 93 |
| H12E | 5488.75 | 1406.7 | 6741.24 | 93 |
| H12F | 6371.25 | 1053.04 | 6683.11 | 93 |
| H12G | 6555.25 | 405.84 | 5361.11 | 115 |
| H12H | 5749.29 | 383.36 | 5202.22 | 115 |
| H12I | 6569.45 | 289.74 | 4539.29 | 154 |
| H12J | 7228.26 | -217.89 | 4853.78 | 154 |
| H13D | 5807.81 | -302.6 | 4559.68 | 178 |
| H13E | 6644.92 | -638.39 | 4391.36 | 178 |
| H13F | 6956.12 | -1165.79 | 5148.39 | 159 |
| H13G | 6193.56 | -1236.58 | 4968.18 | 159 |
| H13H | 6033.61 | -1107.38 | 5776.14 | 124 |
| H13I | 5429.83 | -573.67 | 5442.94 | 124 |
| H134 | 7612.7 | -33.54 | 5643.86 | 83 |
| H138 | 7016.18 | -1443.96 | 6156.61 | 90 |
| H13J | 9416.02 | -482.76 | 5718.41 | 151 |
| H13K | 9318.8 | -433.99 | 6268.38 | 151 |
| H13L | 8768.27 | 49.49 | 5871.58 | 151 |
| H14D | 8353.32 | -2159.9 | 6949.82 | 155 |
| H14E | 8807.95 | -2388.3 | 6500.62 | 155 |
| H14F | 7914.28 | -2313.42 | 6555.57 | 155 |
| H143 | 11595.66 | -1871.85 | 6264.86 | 120 |
| H144 | 12567 | -2222.5 | 6859.42 | 156 |
| H150 | 8904.79 | -2199.75 | 9525.15 | 126 |
| H152 | 9559.36 | -811.33 | 9177.6 | 109 |
| H15A | 9763.72 | -751.76 | 8004.53 | 267 |
| H15B | 10607.28 | -1056.4 | 8231.03 | 267 |
| H15C | 10024.09 | -532.15 | 8433.65 | 267 |
| H15D | 9926.7 | -1862.62 | 10270.96 | 75 |
| H15E | 9945.5 | -1222.65 | 10093.15 | 75 |
| H15F | 9920.03 | -1453.15 | 10942.14 | 88 |
| H15G | 9181.66 | -923.19 | 10738.57 | 88 |
| H15H | 8770.52 | -1549.7 | 11324.75 | 94 |
| H15I | 9203.33 | -2080.24 | 11075.89 | 94 |
| H16C | 7872.89 | -1140.83 | 10692.79 | 82 |
| H16D | 7893.69 | -1790.47 | 10877.78 | 82 |
| H16E | 8703.19 | -2080.9 | 10227.22 | 78 |
| H16F | 7933.46 | -1587.1 | 10017.74 | 78 |
| H163 | 7355.74 | -715.76 | 9824.04 | 72 |
| H167 | 9354.46 | -383.54 | 9828.35 | 85 |
| H16G | 8736.07 | 1027.22 | 9263.63 | 154 |
| H16H | 9439.86 | 540.47 | 9557.99 | 154 |
| H16I | 8802.38 | 968.01 | 9828.2 | 154 |
| H16J | 6110.96 | 473.08 | 9894.14 | 111 |
| H16K | 6198.82 | -2.29 | 9581.14 | 111 |
| H16L | 6183.14 | 629.07 | 9326.74 | 111 |
| H172 | 5454.72 | 3651.49 | 9080.58 | 116 |
| H173 | 5784.45 | 3310.08 | 9887.14 | 140 |
| H174 | 6372.59 | 2332.94 | 10177.88 | 114 |
| H176 | 6319.68 | 2022.73 | 8850.78 | 74 |
| H20C | 1469.15 | 4846.32 | 3684.44 | 268 |
| H20D | 625.66 | 4873.25 | 3868.24 | 268 |
| H20E | 812.87 | 4914.52 | 3312.19 | 268 |
| H30A | 968.39 | 5717.94 | 4896.98 | 172 |
| H30B | 562.15 | 5335.15 | 4697.25 | 172 |
| H30C | 1477.18 | 5159.54 | 4705.84 | 172 |
| H70C | 7046.96 | 1645.23 | 4850.66 | 347 |
| H70D | 6493.36 | 2227.34 | 4986.17 | 347 |
| H70E | 6236.91 | 1665.99 | 5090.49 | 347 |
| H1AA | 7715.51 | 2198.22 | 5378.88 | 164 |
| H1AB | 8186.95 | 1560.4 | 5317.28 | 164 |
| H1AC | 8136.46 | 1728.52 | 5830.46 | 164 |
| H50D | 7807.66 | -351.55 | 6970.64 | 268 |
| H50E | 8388.25 | -275.05 | 7327.4 | 268 |
| H50F | 7485.56 | -60.59 | 7419.42 | 268 |
| H80A | 9028.6 | 540.23 | 6585.91 | 115 |
| H80B | 9706.02 | 772.59 | 6358.49 | 115 |
| H80C | 9069.49 | 1137.02 | 6673.86 | 115 |
| H60A | 4012.35 | 4813.24 | 7178.92 | 110 |
| H60B | 4401.85 | 4196.9 | 7506.82 | 110 |
| H60C | 4470.98 | 4281.49 | 6938.7 | 110 |
| H80D | 10591.06 | -225.35 | 6654.12 | 89 |
| H80E | 9951 | -456.85 | 6942.16 | 89 |
| H80F | 10747.71 | -555.94 | 7197.61 | 89 |
| H20F | 849.29 | 4016.97 | 4478.06 | 183 |
| H20G | 1737.38 | 3965.91 | 4449.25 | 183 |
| H20H | 1485.48 | 3412.64 | 4461.07 | 183 |
| H50G | 7126.49 | -934.44 | 7059.47 | 319 |
| H50H | 6716.4 | -976.96 | 7566.4 | 319 |
| H50I | 7276.22 | -1543.55 | 7409.3 | 319 |
| H60D | 4919.87 | 4746.56 | 8021.04 | 202 |
| H60E | 4505.84 | 5319.85 | 7645.86 | 202 |
| H60F | 5379.32 | 5178.37 | 7801.07 | 202 |
| H0AA | 7633.58 | 2883.11 | 4233.55 | 242 |
| H0AB | 8520.83 | 2527.33 | 4196.25 | 242 |
| H0AC | 8115.93 | 3060.36 | 3781.99 | 242 |
| H07A | 9613.98 | 2770.66 | 4223.54 | 261 |
| H07B | 9708.71 | 3100.56 | 4626.5 | 261 |
| H07C | 9568.38 | 3428.95 | 4081.77 | 261 |
| H30D | 1928.12 | 5267.6 | 5651.65 | 168 |
| H30E | 2319.37 | 4731.8 | 5401.78 | 168 |
| H30F | 2062.2 | 4630.68 | 5946.78 | 168 |
| H15J | 9663.43 | -2941.98 | 8840.03 | 306 |
| H15K | 9253.97 | -2527.67 | 8360.21 | 306 |
| H15L | 8759.81 | -2615.34 | 8829.27 | 306 |
| H2AA | 9230.19 | 1219.19 | 4904.37 | 309 |
| H2AB | 9771.17 | 574.84 | 4916.24 | 309 |
| H2AC | 8990.6 | 696.34 | 5209.58 | 309 |
| H4AA | 8212.64 | 415.46 | 5000.21 | 350 |
| H4AB | 8836.26 | -43.69 | 4749.77 | 350 |
| H4AC | 8024.89 | 312.16 | 4493.36 | 350 |
| H38C | 2217.61 | -346.95 | 9185.06 | 72 |
| H38D | 1807.62 | 254.9 | 8836.55 | 72 |
| H39C | 913.49 | -150.21 | 8578.46 | 88 |
| H39D | 846.37 | -162.77 | 9141.17 | 88 |
| H40C | 1609.65 | -1141.26 | 9308.84 | 104 |
| H40D | 900.67 | -1071.88 | 8976.59 | 104 |
| H41C | 2077.02 | -1666.39 | 8703.77 | 96 |
| H41D | 1689.14 | -1112.84 | 8299.08 | 96 |
| H42C | 3025.24 | -1253.68 | 8403.84 | 75 |
| H42D | 2865.17 | -1177.42 | 8946.34 | 75 |
| H75A | 8822.32 | 779.34 | 6321.34 | 129 |
| H75B | 9720.27 | 693.49 | 6282.27 | 129 |
| H75C | 9191.98 | 1118.78 | 6601.85 | 129 |
| H1AD | 10407 | -674.79 | 7104.89 | 183 |
| H1AE | 10478.56 | -316.97 | 7492.07 | 183 |
| H1AF | 10762.22 | -163.87 | 6958.44 | 183 |

Table 8 Atomic Occupancy for exp\_2788.

| Atom | *Occupancy* |  | Atom | *Occupancy* |  | Atom | *Occupancy* |
| --- | --- | --- | --- | --- | --- | --- | --- |
| C37 | 0.621(7) |  | C38 | 0.621(7) |  | H38A | 0.621(7) |
| H38B | 0.621(7) |  | C39 | 0.621(7) |  | H39A | 0.621(7) |
| H39B | 0.621(7) |  | C40 | 0.621(7) |  | H40A | 0.621(7) |
| H40B | 0.621(7) |  | C41 | 0.621(7) |  | H41A | 0.621(7) |
| H41B | 0.621(7) |  | C42 | 0.621(7) |  | H42A | 0.621(7) |
| H42B | 0.621(7) |  | S800 | 0.676(4) |  | C801 | 0.676(4) |
| H80A | 0.676(4) |  | H80B | 0.676(4) |  | H80C | 0.676(4) |
| C800 | 0.676(4) |  | H80D | 0.676(4) |  | H80E | 0.676(4) |
| H80F | 0.676(4) |  | O800 | 0.676(4) |  | C37A | 0.379(7) |
| C38A | 0.379(7) |  | H38C | 0.379(7) |  | H38D | 0.379(7) |
| C39A | 0.379(7) |  | H39C | 0.379(7) |  | H39D | 0.379(7) |
| C40A | 0.379(7) |  | H40C | 0.379(7) |  | H40D | 0.379(7) |
| C41A | 0.379(7) |  | H41C | 0.379(7) |  | H41D | 0.379(7) |
| C42A | 0.379(7) |  | H42C | 0.379(7) |  | H42D | 0.379(7) |
| S8 | 0.324(4) |  | C75 | 0.324(4) |  | H75A | 0.324(4) |
| H75B | 0.324(4) |  | H75C | 0.324(4) |  | C1A | 0.324(4) |
| H1AD | 0.324(4) |  | H1AE | 0.324(4) |  | H1AF | 0.324(4) |
| O13 | 0.324(4) |  |  |  |  |  |

Table 9 Solvent masks information for exp\_2788.

| Number | X | Y | Z | Volume | Electron count | Content |
| --- | --- | --- | --- | --- | --- | --- |
| 1 | 0.042 | -0.037 | 0.133 | 7.2 | 0.0 | ? |
| 2 | -0.042 | 0.037 | 0.867 | 7.2 | 0.0 | ? |
| 3 | -0.058 | 0.200 | 0.520 | 11.2 | 0.0 | ? |
| 4 | -0.822 | 0.500 | 1.000 | 3679.2 | 166.0 | 4 C2H6SO |
| 5 | 0.058 | 0.800 | 0.480 | 11.2 | 0.0 | ? |
| 6 | 0.296 | 0.442 | 0.671 | 18.9 | 0.0 | ? |
| 7 | 0.704 | 0.558 | 0.329 | 18.9 | 0.0 | ? |

Experimental

Single crystals of C197H248N15O22S10
[exp\_2788]
were
[].
A suitable crystal was selected and
[]
on a
XtaLAB AFC12 (RINC): Kappa dual home/near
diffractometer. The crystal was kept at 236(90) K during data collection.
Using Olex2 [1], the structure was solved with the
SHELXT
[2] structure solution program using
Intrinsic Phasing
and refined with the
SHELXL
[3] refinement package using
Least Squares
minimisation.

1. Dolomanov, O.V., Bourhis, L.J., Gildea, R.J, Howard, J.A.K. & Puschmann, H.
   (2009), J. Appl. Cryst. 42, 339-341.
2. Sheldrick, G.M. (2015). Acta Cryst. A71, 3-8.
3. Sheldrick, G.M. (2015). Acta Cryst. C71, 3-8.

Crystal structure determination of
[exp\_2788]

**Crystal Data**
for C197H248N15O22S10 (*M*=3498.69 g/mol):
triclinic, space group P-1 (no. 2),
*a* = 18.2626(3) Å, *b* = 24.9409(4) Å, *c* = 28.3395(3) Å, *α* = 78.2060(10)°, *β* = 86.9020(10)°, *γ* = 71.0290(10)°,
*V*= 11948.3(3) Å3,
*Z* = 2,
*T* = 236(90) K,
μ(CuKα) = 1.286 mm-1,
*Dcalc* = 0.972 g/cm3,
131463 reflections measured (7.716° ≤ 2Θ ≤ 134.158°),
42378 unique (*R*int = 0.1143, Rsigma = 0.0920) which were used in all calculations.
The final *R*1 was 0.1061
(I > 2σ(I)) and *wR*2 was 0.2727 (all data).

Refinement model description

Number of restraints - 376,
number of constraints - unknown.

Details:

```
1. Fixed Uiso
```

This report has been created with Olex2, compiled on
2023.08.24 svn.re1ec1418 for OlexSys. Please
let us know
if there are any errors or if you would like to have additional features.
